# Engineered flavonoid disrupts mitochondrial AIF/CHCHD4 complex for targeted cancer therapy

**DOI:** 10.1016/j.apsb.2026.06.022

**Published:** 2026-06-15

**Authors:** Hong Toan Lai, Daniela Dias-Pedroso, Maria Eugénia Marques Da Costa, Romain Fernandes, Tran Ngoc Anh Nguyen, Olivia Bawa, Pierre Khneisser, João Gabriel Silva, Yassine Khalij, Michelangelo Campanella, John Griffith Jones, Svetlana Dokudovskaya, Guido Kroemer, Antonin Marchais, Nathalie Gaspar, Birgit Geoerger, Oumar Diane, Liuba Mazzanti, Tâp Ha Duong, Guy Lewin, Laurent Ferrié, Bruno Figadère, Catherine Brenner

**Affiliations:** a CNRS, Institut Gustave Roussy, Biologie du cancer et métabolisme, Université Paris-Saclay, Villejuif 94805, France; b INSERM UMR 1015, Gustave Roussy Cancer Campus, Villejuif 94805, France; c INSERM US23, CNRS UAR 3655, AMMICa, Experimental and Translational Pathology Platform, Gustave Roussy, Villejuif 94805, France; d Department of Pathology, Gustave Roussy Cancer Campus, Villejuif 94805, France; e Université Paris-Saclay, Institut Gustave Roussy, Inserm, Immunologie anti-tumorale et immunothérapie des cancers, Villejuif 94805, France; f Centre for Innovative Biomedicine and Biotechnology, and III-UC-Institute of Interdisciplinary Research, University of Coimbra, Coimbra 3004-504, Portugal; g Centre de Recherche des Cordeliers, Equipe labellisée par la Ligue contre le cancer, Université Paris Cité, Sorbonne Université, Inserm U1138, Institut Universitaire de France, Paris 75006, France; h Metabolomics and Cell Biology Platforms, Institut Gustave Roussy, Villejuif 94805, France; i Institut du Cancer Paris CARPEM, Department of Biology, Hôpital Européen Georges Pompidou, AP-HP, Paris 75015, France; j Department of Oncology for child and adolescent, Gustave Roussy, Villejuif 94805, France; k BioCIS, CNRS, Université Paris-Saclay, Orsay 91400, France

**Keywords:** Antineoplastics, Energy homeostasis, Mitochondrial protein import, Oncometabolism, Small molecule, Metabolism, Osteosarcoma, Flavonoid

## Abstract

Cancer metabolism depends on multifaceted mechanisms, including bidirectional inter-organelle communication between mitochondria and the nucleus, facilitating cellular adaptation at the transcriptomic, proteomic, and metabolomic levels. The mitochondrial protein complex composed of apoptosis-inducing factor (AIF) and coiled-coil-helix-coiled-coil-helix domain-containing protein 4 (CHCHD4) is essential for this mitochondrio-nuclear communication. The AIF/CHCHD4 complex mediates the mitochondrial import of cysteine-enriched nuclear gene-encoded proteins, thereby adapting the mitochondrial proteome to cellular energy demands. Here, we report the discovery of M30-E05, a compound that binds to the NADH pocket of AIF, preventing its dimerization and disrupting the AIF/CHCHD4 complex, as demonstrated by molecular docking and gel electrophoresis analysis of mitochondrial AIF/CHCHD4 substrate expression. In cancer cells, M30-E05 reduces the expression of nuclear gene-encoded mitochondrial proteins such as AIF, CHCHD4, cytochrome *c* oxidase copper chaperone (COX17), and Mitochondrial calcium uptake 1 (MICU1). In addition, M30-E05 fragments the mitochondrial network and impairs mitochondrial respiration, causing profound alterations, particularly in glucose, lipid, and amino acid metabolism, as revealed by kinetic measurements of oxygen consumption and mass spectrometric metabolomics. Importantly, M30-E05 significantly reduces the viability of a human adult and pediatric osteosarcoma cancer cell panel, including those from patient-derived xenografts (PDX) of osteosarcomas, and induces apoptosis. When orally administered for two weeks to immunodeficient NSG mice, M30-E05 inhibited tumor growth in a subcutaneous PDX xenograft model without apparent toxicity. We anticipate that M30-E05, as a first-in-class metabolic inhibitor, could serve as the lead compound for a new class of antineoplastic agents.

## Graphical abstract

M30-E05, as a first-in-class metabolic inhibitor, could serve as a lead compound for a new class of antineoplastic agents.

**Figure:**
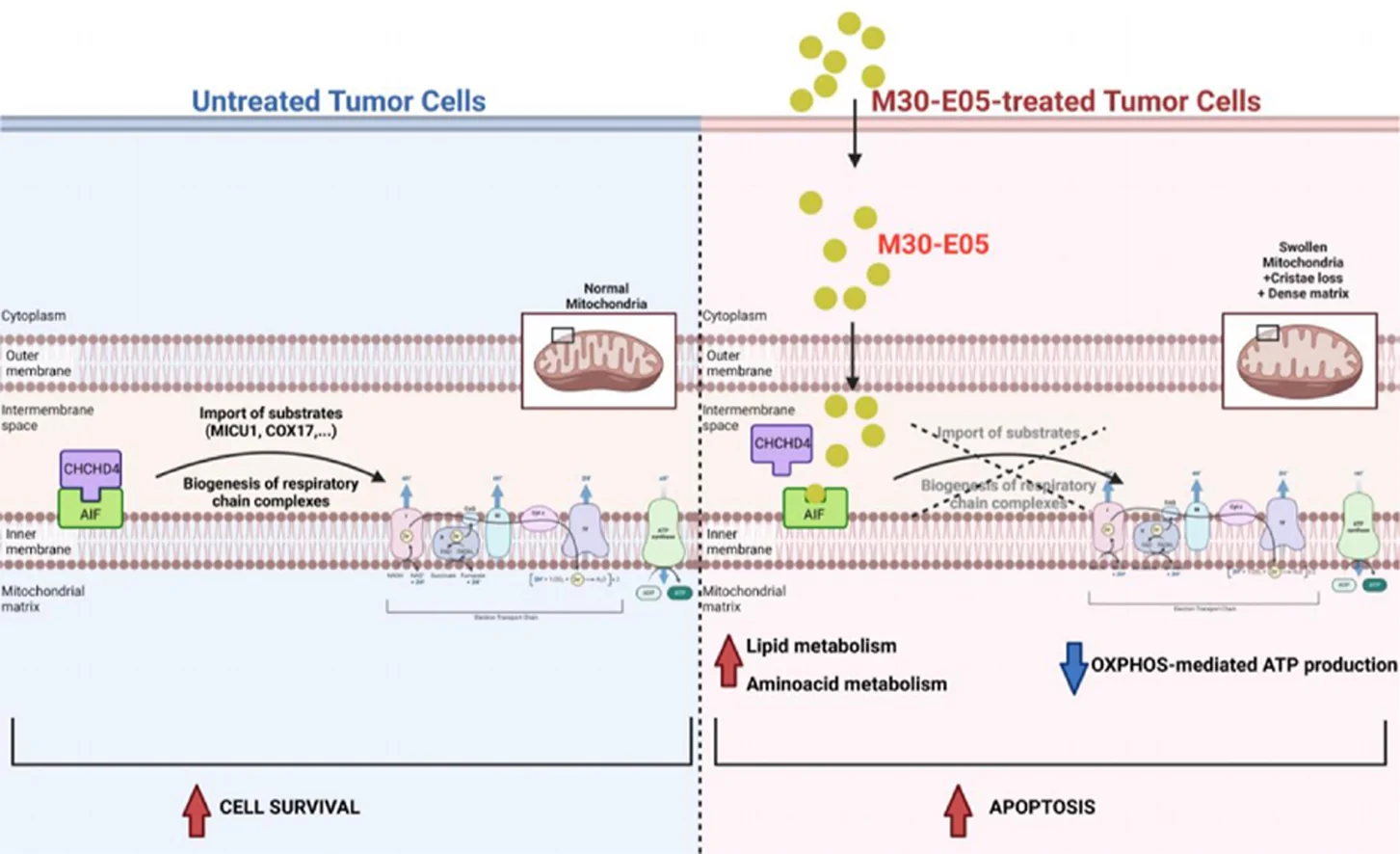

## 1 Introduction

The first theory of cancer metabolic reprogramming, proposed by Otto Warburg^1^, describes a shift in adenosine triphosphate (ATP) production from mitochondrial oxidative phosphorylation to glycolysis, which may contribute to the higher proliferative rate of cancer cells. Today, the concept of metabolic reprogramming has expanded beyond energy metabolism and includes explanations for the formidable capacity of cancer cells to engage in anabolic reactions^2^. Advances in our understanding of tumor biology and its interaction with the microenvironment have led to the recognition of dysregulated cellular metabolism as a new hallmark of cancer^3^^,^^4^^,^^5^. Indeed, in cancer patients, hypermetabolism, which can be detected by PET scan imaging, is typically associated with poor survival rates and can predict cachexia and relapse. Cancer cells exploit their intrinsic metabolic flexibility, enabling them to switch between different energy substrates and to equilibrate anabolism and catabolism to survive within the tumor microenvironment. Building on these observations, we hypothesized that small molecules that subvert this metabolic flexibility could be used to inhibit tumor progression^6^.

Cancer metabolism depends on bidirectional inter-organelle communication between mitochondria and the nucleus, which allows cellular adaptation at the transcriptomic, metabolomic, and proteomic levels, ultimately promoting cancer progression^7^^,^^8^. Most of the ∼1500 mitochondrial proteins are encoded by the nuclear genome and imported into the mitochondria^9^. To meet cancer cell-specific needs, several specialized protein import machineries have evolved, adapting the mitochondrial proteome accordingly. Within these import pathways, the disulfide relay machinery relies on the formation of a complex between apoptosis-inducing factor (AIF, encoded by the *AIFM1* gene) and coiled-coil-helix domain-containing protein 4 (CHCHD4) complex. This complex regulates the redox-sensitive import of small, cysteine-containing proteins, critical for cell survival and stress adaptation^10^^,^^7^. Monomeric, dimeric AIF and CHCHD4 contribute to mitochondrial bioenergetics, redox regulation, lipid and calcium homeostasis, and to the maintenance of mitochondrial ultrastructure^7^. Genetic alterations affecting AIF influence mitochondrial biology and cell fate through the biogenesis of the respiratory chain and the import of key nuclear-encoded mitochondrial proteins, such as mitochondrial calcium uptake 1 (MICU1), cytochrome *c* oxidase copper chaperone (COX17), chromosome 9 open reading frame 72 (C9orf72), NADH:ubiquinone oxidoreductase subunit S5 (NDUFS5) and TP53 regulated inhibitor of apoptosis 1 (TRIAP1)^11^, ^12^, ^13^, ^14^. Loss-of-function mutations in *AIFM1* are linked to a range of X-linked mitochondrial diseases^15^. In contrast, the potential oncogenic role of unmutated *AIFM1* has been demonstrated in a mouse model of *KRAS*-mutant lung cancer^16^. Similarly, CHCHD4 favors tumor proliferation and epithelial-to-mesenchymal transition-related phenotypes through respiratory chain-mediated metabolism^17^.

To test whether disrupting the AIF/CHCHD4 protein complex could reduce metabolic flexibility and promote cancer cell death, we designed an original, highly specific, high-throughput bioassay to identify inhibitors disrupting this complex^18^. Using this assay, we screened several libraries, including the Prestwick Library of FDA-approved drugs, a subset of the French National Chemical Library, and a BioCIS collection of new molecules with diverse chemical structures. Notably, this BioCIS collection included derivatives of flavonoids, chemically modified natural compounds, that show promise for therapeutic applications with minimal toxicity. As a result, the screening identified several potential AIF ligands (*i.e.*, “hits”) that were ranked based on their ability to disrupt the AIF/CHCHD4 complex. These hits were further evaluated for their pharmacological activity through *in silico* analysis, *in cellulo* models including both parental and drug-resistant cancer cell lines, secondary cultures of osteosarcoma patient-derived xenografts (PDX), as well as *in vivo* PDX models.

Our findings show that M30-E05 targets cancer metabolism and induces apoptosis by disrupting the AIF/CHCHD4 complex in a pediatric and adult cancer cell line panel, significantly reducing osteosarcoma tumor growth *in vivo*. Given that metabolic reprogramming is a universal feature of cancer, M30-E05 represents a promising lead for the development of a novel class of antineoplastic agents.

## 2 Materials and methods

### 2.1 Chemicals

M30-E05 has been synthesized in the BioCIS laboratory (details provided in Supporting Information). The compound was solubilized in dimethyl sulfoxide (DMSO) at 25 or 50 mmol/L stock solutions and stored at −20 °C. For *in vitro* experiments, M30-E05 was washed with a 1:1 chloroform: methanol solution, air-dried overnight using Speedvac (Thermo Scientific, Courtabœuf, France), before solubilization. For *in vivo* studies, the compound was solubilized in 0.5% methylcellulose (M7027, Merck, Saint-Quentin-Fallavier, France) freshly before use.

### 2.2 Screening on the inhibition of interaction between HIS-AIF_103-613_ and GST-CHCHD4

HIS-AIF_103-613_ and GST-CHCHD4 were produced in BL21+DE3(RIPL) bacteria during a 3 h-induction with 0.5 mmol/L IPTG at 37 °C, as previously described^19^. Assays were performed in white 384-well microplates (781075, Greiner, Courtabœuf, France) with a total volume of 25 μL per well. Proteins, peptide N27 (positive control)^19^, and beads were diluted in 0.1% bovine serum albumin (BSA)/phosphate-buffered saline (PBS), on ice.

A total of 1.280 off-patent small molecule library provided by Prestwick Chemicals; 2.901 compounds provided by the French National Library, and 99 compounds provided by the BioCIS library were screened at 10 μmol/L in 0.1% DMSO in monoplicate. Firstly, 5 μL of 10 μmol/L compound or 0.1% DMSO/3 μmol/L N27 or 0.1% DMSO/0.1% BSA PBS (negative control) was added. Secondly, 5 μL of His-AIF_103-613_ at 300 nmol/L were incubated with compounds for 20 min before adding 5 μL CHCHD4-Gst at 100 nmol/L for 2 h. Thirdly, 10 μL of a mixture of 5 μg/mL donor and acceptor beads were incubated for 3 h at 23 °C in the dark until reading on EnSpire® multimode plate reader (PerkinElmer, Villepinte, France)^18^. For confirmation, the best hits (including M30-E05) were validated in triplicate at concentrations of 10 and 30 μmol/L.

### 2.3 Cell lines and patient-derived xenografts (PDX)

Human pediatric osteosarcoma cell lines HOS parental (HOS), HOS-doxorubicin resistant (HOS-R/DOXO), HOS-methotrexate resistant (HOS-R/MTX), MG63, U2OS, 143B, SAOS2, and SAOS2-R/MTX were generously provided by Dr N. Gaspar^20^ and Dr B. Geoerger. Adult cancer cell lines tested, which include A549 (non-small cell lung cancer), IGROV-1 (human ovarian carcinoma), IGROV-1 CDDP^R^ (cisplatin-resistant IGROV-1), IGROV-1 VC^R^ (vincristine-resistant IGROV-1), Capan-1 (pancreatic adenocarcinoma), HL-60 (acute promyelocytic leukemia), Jurkat (T-cell leukemia precursor), and NAML6 (acute lymphoblastic leukemia) and the epithelial line of the kidney of a human embryo HEK293 and the proximal tubular cell line derived from normal kidney HK-2 (human kidney 2) were provided by Dr J. Wiels (CNRS UMR 9018, Gustave Roussy, Villejuif). All the cell lines were routinely cultured in Dulbecco's modified Eagle medium (DMEM, high glucose, GlutaMAX Supplement, Gibco, Courtabœuf, France) supplemented with 10% fetal bovine serum (FBS, Paraguay Origin, Sigma‒Aldrich, Saint-Quentin-Fallavier, France) and 1% Penicillin‒Streptomycin (Gibco, Courtabœuf, France) at 37 °C in a humidified atmosphere (5% CO_2_ and 95% air), under mycoplasma-free conditions. The PDXs GR-OS-9, GR-OS-10, GR-OS-11, GR-OS-12, GR-OS-15, GR-OS-18, and GR-OS-20 were established from relapsed or refractory osteosarcoma within the MAPPYACTS PDX project^21^ and kindly provided by Dr B. Geoerger and Dr ME Marques Da Costa. Of note, FBS concentration for the *in vitro* PDX secondary culture was 20%.

Human blood samples were obtained from the ‘Etablissement Français du Sang’, Hôpital Saint-Louis, Paris, France, and peripheral blood mononuclear cells (PBMC) were freshly prepared by density gradient centrifugation.

### 2.4 siRNA transfection

To knock down AIF expression for evaluating the specificity of M30-E05, HOS cells were seeded at densities of 12.500, 18.750, 25.000, 31.250 HOS cells/cm^3^ and transfected with ON-TARGETplus Human *AIFM1* siRNA (L-011912-00-0005, Horizon Discovery, Cambridge, UK) or ON-TARGETplus *GAPDH* Control Pool (D-001830-10-05, Horizon Discovery, Cambridge, UK) for 96, 72, 48, 24 h, using 0.2, 0.16, 0.2 and 0.08 μL of DharmaFECT 1 Transfection Reagent (T-2001-03, Horizon Discovery, Cambridge, UK), respectively, according to the manufacturer's instructions. For subsequent experiments, the 48-h time point was used. siRNA pool targeting AIF, comprised of 4 selected siRNA duplexes each, has a modification pattern that addresses off-target effects caused by both strands (SMART pool).

### 2.5 Transcriptomic analysis

Under the approval by the steering committee of the MAPPYACTS trial (NCT02613962, Dr B. Geoerger) for 42 refractory or relapsed OS patients and UNICANCER Transbone scientific committee of the OS2006 trial (NCT00470223–Dr N Gaspar) for 82 diagnostic OS patients, expression of AIF and CHCHD4 was assessed by RNA-sequencing. Mann‒Whitney U tests were used to analyze differential expression between diagnostic, non-resistant, and resistant OS tumor samples.

### 2.6 Data availability

Sequencing data and basic clinical annotations from all patients and PDX have been deposited in the BoOSTDataS health data host (https://boostdatasdb.org).

### 2.7 Gene set enrichment analysis

Cell line RNA-sequencing results were obtained by Dr N. Gaspar and A. Marchais's team. Gene set enrichment analysis was performed using the gseGO function in the clusterProfiler R package, applying the KEGG pathway enrichment to reveal the most significant dysregulated pathways.

### 2.8 Gene ontology (GO)

Total RNAs were isolated using the NucleoSpin® RNA (740955, Macherey-Nagel, Hoerdt, France) according to the manufacturer's instructions, stored at −80 °C. RNA quality control was performed on NanoDrop™ 2000 (Thermo Scientific, Courtabœuf, France). RNA-sequencing results were obtained by Novogene (Cambridge, UK). Differentially expressed genes (DEGs) between untreated HOS cells and the M30-E05 IC_50-_treated HOS cells were identified using the DESeq2 package (absolute log2 fold change >1 and adjusted *P*-value <0.05). Therefore, the DEGs were uploaded into DAVID bioinformatics tool (Database for Annotation, Visualization and Integrated Discovery) and analyzed for enriched GO terms in the biological process category.

### 2.9 Metabolomics

#### 2.9.1 Sample preparation

HOS, HOS-R/DOXO, HOS-R/MTX (300.000 cells per well) were cultured in 6 well plates and treated with IC_50_ of M30-E05 for 72 h. Cells were gently rinsed with cold PBS and lysed by adding 500 μL of cold methanol/water (9/1, *v*/*v*, −20 °C) with internal standards. After a quick centrifugation, supernatants were pooled, with two wells per condition in a single microtube. Supernatants in microtubes were vortexed at 800×*g* for 5 min and centrifuged at 15,000×*g* for 10 min at 4 °C. Supernatants were split into two parts: 150 μL were used for GC‒MS experiment in the injection vial, and 300 μL were used for UHPLC‒MS experimentation. LC‒MS aliquots were evaporated, and dried extracts were resuspended in 150 μL of MilliQ water. Samples were kept at −80 °C until injection or transferred to vials for direct analysis by UHPLC/MS.

After evaporation, the dry GC‒MS aliquot was spiked with 50 μL of methoxyamine (20 mg/mL in pyridine) and stored at room temperature in the dark overnight. Then, 80 μL of MSTFA was added, and final derivatization occurred at 40 °C for 30 min. Samples were directly injected into GC‒MS.

#### 2.9.2 Targeted analysis of nucleotides and cofactors by ion pairing ultra-high performance liquid chromatography (UHPLC) coupled to a triple quadrupole mass spectrometer

Targeted analysis was performed on a RRLC 1290 system (Agilent Technologies) coupled to a Triple Quadrupole 6470 (Agilent Technologies) equipped with an electrospray source. 10 μL of sample was injected on a Column Zorbax Eclipse XDB-C18 (100 mm × 2.1 mm particle size 1.8 μm) from Agilent Technologies. The gradient mobile phase consisted of water with 2 mmol/L of dibutylamine acetate concentrate (phase A) and acetonitrile (phase B). Flow rate was set to 0.4 mL/min, and gradient was performed as follows: initial condition was 90% phase A and 20% phase B, maintained for 3 min. Molecules were then eluted using a gradient from 10% to 95% phase B over 1 min. The column was washed using 95% mobile phase B for 2 min and equilibrated using 10% mobile phase B for 1 min. The scan mode used was the MRM for biological samples. Peak detection and integration of the analytes were performed using the Agilent Mass Hunter quantitative software (B.10.1).

#### 2.9.3 Widely-targeted analysis of intracellular metabolites by gas chromatography (GC) coupled to a triple quadrupole mass spectrometer

GC‒MS/MS method was performed on a coupling gas chromatography/triple quadrupole 7890B/7000C (Agilent Technologies, Waldbronn, Germany). The scan mode used was the MRM for biological samples. Peak detection and integration of analytes were performed using the Agilent Mass Hunter quantitative software (B.07.01), exported as tables, and processed with R software (version 4.0.3) and the GRMeta package (Github/kroemerlab).

### 2.10 Western blot

Immunoblotting was performed on cell lysates in NETN buffer supplemented with antiprotease cocktail (A32963, ThermoFisher, Courtabœuf, France). Briefly, the cell lysates were separated on a 10%–15% Tris-glycine SDS–PAGE gel (NP0322, Invitrogen, Courtabœuf, France) and transferred onto a PVDF membrane. Membranes were blocked in 5% milk in TBS with 0.1% TWEEN-20 (TBST) for 1 h at room temperature, followed by overnight incubation with indicated primary antibodies at 1:1000 [MICU1 (HPA037479, ATLAS Antibodies, Stockholm, Sweden), MICU2 (HPA045511, ATLAS Antibodies, Stockholm, Sweden), MCU (AMAB91189, ATLAS Antibodies, Stockholm, Sweden), AIF (5318S, Cell Signaling, Leiden, Netherlands), CHCHD4 (21090-1-AP, Proteintech, Planegg, Germany), Vinculin (ab219649, Abcam, Amsterdam, Netherlands), GAPDH (2118, Cell Signaling, Leiden, Netherlands), Caspase-3 (9662, Cell Signaling, Leiden, Netherlands), Cleaved-Caspase 3 (9661, Cell Signaling, Leiden, Netherlands), Caspase-9 (9502, Cell Signaling, Leiden, Netherlands), Cleaved-Caspase 9 (9505, Cell Signaling, Leiden, Netherlands)] diluted in TBST containing 5% BSA. Membranes were washed and incubated with secondary antibody at 1:5000 dilution (Horseradish peroxidase (HRP)-labeled rabbit anti-mouse (315-035-003, Jackson ImmunoResearch, Cambridge, UK) and goat anti-rabbit antibodies (111-035-144, Jackson ImmunoResearch, Cambridge, UK) for 2 h at room temperature. Bands were visualized using the Substrat HRP Immobilon Western (WBKLS0500, Millipore, Saint-Quentin-Fallavier, France) and imaged with Amersham™ ImageQuant™ 800 (Cytiva, Saint-Germain-en-Laye, France). Equal loading was verified by immunoblotting with Vinculin and GAPDH, then normalization of results was performed using free ImageJ software.

### 2.11 Co-immunoprecipitation

Eight million HOS cells were plated in 145 cm^2^ Petri dishes (168381, Thermo Fisher, Courtabœuf, France) one day before treatment. Cells were treated with 8.3 μmol/L M30-E05 or 0.002% DMSO for the indicated times (1, 2, 4, and 6 h). All cells were collected using a cell scraper, and lysates were prepared in 1% NP-40 lysis buffer (50 mmol/L Tris, pH 8.0, 150 mmol/L NaCl, 1 mmol/L MgCl_2_, 1 mmol/L CaCl_2_, 10% glycerol, phosphatase inhibitor (Roche, Saint-Quentin-Fallavier, France), protease inhibitor cocktail (Roche, Saint-Quentin-Fallavier, France). Lysates were incubated on ice for 30 min and centrifuged at 4 °C at maximum speed for 10 min. For immunoprecipitation, magnetic Dynabeads M-280 (Invitrogen, Courtabœuf, France) were saturated with 1% BSA for 1 h to reduce nonspecific binding. Two micrograms of primary antibodies (anti-rabbit IgG control, #30000-0-AP, Proteintech, Planegg, Germany; anti-CHCHD4, 1:1000, 21090-1-AP, Proteintech, Planegg, Germany) diluted in PBS with 0.5% Tween-20 (PBS-T) were added to the beads and incubated at room temperature with rotation for 20 min. Antibody-bead complexes were washed once with PBS-T, and protein samples were added, followed by overnight incubation at 4 °C with rotation. Twenty-five micrograms of protein lysates were reserved as input. Antigen‒antibody‒bead complexes were washed twice with PBS-T, denatured, and reduced by boiling for 10 min in NuPAGE LDS sample buffer 4X (Thermo Scientific, Courtabœuf, France) containing DTT. Western blot analysis was performed as described, using antibodies against CHCHD4 (1:1000, 21090-1-AP, Proteintech, Planegg, Germany), AIF (1:1000, 5318S, Cell Signaling, Leiden, Netherlands), and goat anti-rabbit secondary antibodies (1:5000, 111-035-144, Jackson ImmunoResearch, Cambridge, UK). Bands were visualized using Substrat HRP Immobilon Western (WBKLS0500, Millipore, Saint-Quentin-Fallavier, France) and imaged with an Amersham™ ImageQuant™ 800 (Cytiva, Saint-Germain-en-Laye, France).

### 2.12 Lactate dehydrogenase assay (LDH)

The half maximal inhibitory concentration (IC_50_) at 72 h of M30-E05, cisplatin (CIS), and Etoposide for all cell lines was determined by CytoTox 96® non-radioactive cytotoxicity assay, following the manufacturer's protocol (G1781, Promega, Charbonnières-les-Bains, France). This assay measures the conversion of a tetrazolium (INT) salt to a formazan product, which presents a reddish color. The intensity of color produced is proportional to the number of cells lysed. Absorbance was measured using a 96-well microplate reader at 490 nm wavelength (TECAN Infinite M200, Lyon, France). The half-maximal inhibitory concentration (IC_50_) was determined using log(agonist) *vs*. response – variable slope equation (*Y*=Bottom + (Top-Bottom)/(1 + 10 ˆ ((LogEC_50_‒*X*)∗HillSlope))) by GraphPad Prism 10.0 software (GraphPad Software Inc., CA, USA).

### 2.13 Cell death analysis

Cells were seeded in 6-well plates at a density of 60.000 cells/well for HOS, and 150.000 cells/wells for all *in vitro* PDX secondary cultures (except 200.000 cells/wells for GR-OS-10 SC and GR-OS-15 SC) and treated with M30-E05 at IC_50_ for 24, 48, 72 h. Culture medium and cells were collected and centrifuged. Supernatant was discarded, and cells were stained with APC Annexin V at 12 μg/mL and propidium iodide (PI) at 0.5 mg/mL (640932, BioLegend, Amsterdam, Netherlands). Data acquisition was performed using the BD Accuri™ C6 Plus Flow Cytometer (San Jose, CA, USA). At least 10.000 events were acquired for each sample, and all data were analyzed using FlowJo™ Software (BD Life Sciences).

### 2.14 SynergyFinder

Evaluation of drug interactions between M30-E05 and conventional chemotherapeutic agents (CIS, Doxorubicin (DOXO), and Methotrexate (MTX) was conducted on parental (HOS) and DOXO- or MTX-resistant (HOS-R/DOXO, HOS-R/MTX) models. HOS (2000 cells/well), HOS-R/DOXO (7000 cells/well), and HOS-R/MTX (5000 cells/well) were seeded in 96-well plates and treated with a combination of M30-E05 and one chemotherapy, in the form of a matrix, for 72 h. Cell viability was measured using the LDH assay, and data were uploaded on the SynergyFinder platform (https://synergyfinder.fimm.fi/). Using the zero-interaction potency (ZIP) model, an overall synergy score was calculated for each matrix, with values below 10 indicating antagonism, between −10 and 10 as additivity, and above 10 as synergism. 2D and 3D interaction maps were also provided, in which the colors green, white, and red demonstrate antagonism, additivity, and synergism, respectively. Experiments were repeated at least 3 times.

### 2.15 Clonogenic assay

HOS cells were seeded at a density of 500 cells/well in 6-well plates. After 24 h, cells were treated with IC_50_ of M30-E05 and kept in the incubator for 10 days under standard conditions. At day 10 after the treatment, colonies were washed twice with PBS and incubated with Crystal Violet (HT90132, Sigma‒Aldrich, Saint-Quentin-Fallavier, France) for 30 min. Images were taken using Amersham™ ImageQuant™ 800 imaging system (Cytiva, Saint-Germain-en-Laye, France).

### 2.16 ATP assay

ATPlite 1-step luminescence assay system from PerkinElmer (6016736, Villepinte, France) was used to evaluate the total ATP contents that reflect the catabolic/anabolic status of the cells by measuring total ATP concentration based on the reaction between ATP with added luciferase and d-luciferine to produce Oxiluciferine and detectable light. Cells were cultured on white 96-well microplates, provided with the kit (PerkinElmer, Villepinte, France), and total ATP content was measured 24 h later with the TECAN Infinite 200 Pro microplate reader.

### 2.17 Bioenergetic profiling using Seahorse XFe96 assay

To determine the metabolic changes in ATP production, parameters such as the rate of cellular mitochondrial respiration and the rate of extracellular acidification, represented respectively by oxygen consumption rate (OCR) and glycolytic efflux rate (GlycoPER), were measured using the Seahorse XFe96 Analyzer (Agilent Technologies, Les Ulis, France) in real-time in 96-well plates. For the untreated conditions, 2.000, 4.000, and 3.000 cells/well were plated 72 h before assay for parental HOS, HOS-R/DOXO and HOS-R/MTX respectively. In the treated conditions, 10.000, 20.000, and 15.000 cells/well were treated during 72 h before assay for parental HOS, HOS-R/DOXO and HOS-R/MTX, respectively. Three different protocols were used to evaluate together or separately OCR and GlycoPER values by injecting different treatments, following manufacturer protocols [Seahorse XF Real-Time ATP Rate Assay (103592-100, Agilent Technologies, Les Ulis, France); Seahorse XF Cell Mito Stress Test (103015-100, Agilent Technologies, Les Ulis, France); Seahorse XF Glycolytic Rate Test (103344-100, Agilent Technologies, Les Ulis, France)]. To count the cells and normalize the Seahorse analysis, cells were incubated with 2.5 μg/mL of Hoechst dye in 150 μL of PBS solution for 15 min at 37 °C and visualized and counted using Cytation 1 imaging system and Gen5 software (Agilent Technologies, Les Ulis, France). Images were captured by DAPI overlay channels or phase contrast. Raw data analysis and normalization were made using Seahorse analytics (Agilent Technologies, Les Ulis, France).

### 2.18 ^13^C NMR tracing and mass-isotopomeric flux analysis

HOS, HOS-R/DOXO and HOS-R/MTX cells were seeded in 56.5 cm^2^ petri dishes (##351029, Falcon) in DMEM, high glucose, GlutaMAX (Gibco, Courtabœuf, France) supplemented with 10% fetal bovine serum (FBS, Paraguay Origin, Sigma‒Aldrich, Saint-Quentin-Fallavier, France) and 1% penicillin‒streptomycin (Gibco, Courtabœuf, France). Seeding density was calculated to give 10 million cells per sample at 72 h. On the following day, cells were treated with IC_50_ of M30-E05 (HOS: 8.3 μmol/L; HOS-R/DOXO: 10.8 μmol/L; HOS-R/MTX: 7.1 μmol/L) in culture media without penicillin‒streptomycin. After 48 h, culture media were replaced with DMEM GlutaMAX, without glucose, supplemented with 10% fetal bovine serum (FBS, Paraguay Origin, Sigma‒Aldrich, Saint-Quentin-Fallavier, France) and 25 mmol/L of [U-^13^C] glucose (#CLM-1396, Cambridge Isotopes *via* Tracer Tecnologías Analíticas, S.L.). Medium samples were collected at several time points until the end of the protocol (1, 12, 24 h) and stored at −80 °C. Cell metabolites were extracted by gently scraping the Petri dishes twice with 750 μL of ice-cold 8:1 methanol: water solution. Samples were centrifuged at 4 °C for 5 min at 5800×*g*, and the aqueous supernatant was collected. The solvents were then evaporated in a Speedvac (AES1010, Savant) overnight at room temperature (without infrared light and with a low drying rate). The dry cell extracts were suspended in 0.55 mL of 99.8% D2O (Eurisotop, Saint-Aubin, France) with pyrazine added as an internal concentration standard. Proton-decoupled ^13^C NMR spectra at 150.8 MHz were acquired with a 14.1 T Magnex Scientific magnet and a Bruker Avance NEO console, and a Bruker 5 mm QCI-F CryoProbe. A pulse width of 90° and 4 s acquisition time corresponding to 285,714 data points were used with a ^13^C spectral width of 237 ppm. The number of scans ranged from 4000 to 16,000. Spectra were processed with 0.5 Hz line-broadening, and multiplets of glutamate and lactate were analyzed by peak deconvolution (ACD Labs, Toronto, Canada). The fractional ^13^C-enrichment of lactate was quantified from ^1^H NMR spectra acquired with the same instrument. These were acquired with 32 scans and acquisition time of 4 s acquisition time corresponding to 76,992 data points over a 16-ppm spectral width, with suppression of the residual water signal. Glutamate multiplet ratios and lactate fractional ^13^C-isotopomer enrichments were applied as inputs to tcaCALC, with the most appropriate metabolic model and relative flux values being identified from the best fit of experimental and modeled data. This was defined by the sum of squares residual error in conjunction with Akaike Information Criterion to guard against overfitting of the data^22^.

### 2.19 Confocal microscopy

In a 12 well-plate, glass coverslips were coated with 100 μg/mL poly-d-lysine at 37 °C, overnight. Each coverslip was washed twice with PBS before cell seeding. For the colocalization experiments, HOS cells were seeded at 55.000 cells/3.5 cm^2^ well on top of the coated coverslips and allowed to adhere for 24 h. Cells were then exposed to IC_50_ (8.29 μmol/L) M30-E05 for 24 or 48 h, and subsequently, cells were fixed with 4% PFA for 20 min, and permeabilized with 2% Triton X-100 in PBS. After blocking for 40 min with 5% BSA in PBS at room temperature, cells were incubated with AIF antibody (1:500, 5318, Cell Signaling, Leiden, Netherlands) and MTC antibody (1:200, NBP2-32980, Novus Biologicals) in 5% BSA for 45 min at room temperature. Cells were then incubated with secondary antibodies Star Orange (1:100, Abberior, Göttingen, Germany), Star Red (1:100, Abberior, Göttingen, Germany), and Hoechst (1:10,000, 62249, Thermofisher, Courtabœuf, France) in 5% BSA for 1 h at room temperature, protected from light. Abberior (Göttingen, Germany) mounting media was used to mount the coverslips. For that, the mounting media was warmed in a water bath at 70 °C until it liquefied and was cooled off to room temperature before being used.

To evaluate the mitochondrial network at basal levels, HOS cells were seeded 110.000 cells/3.5 cm^2^, HOS-R/DOXO cells were seeded 190.000 cells/3.5 cm^2^, HOS-R/MTX cells were seeded 131.000 cells/3.5 cm^2^ and allowed to adhere for 48 h. Fixation and permeabilization were performed as described above. Samples were blocked using 0.5% BSA in PBS for 40 min at room temperature. Cells were incubated with Tom20 (1:500, 42406, Cell Signaling, Leiden, Netherlands) in blocking solution, at 4 °C, overnight, followed by incubation for 1 h with the secondary antibody goat anti-rabbit IgG (H + L) Alexa Fluor™ 555 (1:200) (A-21428, Thermofisher Scientific, Courtabœuf, France) in blocking solution at room temperature. Coverslips were mounted using Fluoroshield™ with DAPI (F6057, Sigma, Sigma‒Aldrich, Saint-Quentin-Fallavier, France). 12-bit numerical images were acquired using a confocal Leica SPE DM4000B microscope (pinhole set at 1.0 Airy unit) equipped with a HC PL APO CS2 63x/1.40 oil-immersion objective, using a 405, 552 and 638 nm lasers. Images were acquired with zoom adjusted to 1.5 and image size 2048 × 2048. Z-stacks, with optimal intervals between slides, were acquired for each sample. Mitochondrial network was accessed in Z-stack projection, using Fiji software^23^. To evaluate the colocalization of AIF in mitochondria or in the nucleus, the Z-stacks of each image were analyzed using the JaCoP plugin in Fiji software^23^^,^^24^. Pearson coefficient, and Manders’ coefficients M1 and M2, after threshold application, were calculated. Fluorescence intensity profiles of AIF, MTC, and Hoechst, following a defined line in x, were evaluated in Z-stack projections of each image. The line was designed to contain at least three nuclei signals.

### 2.20 Transmission electron microscopy

Or ultrastructural studies, cells were fixed in 2% glutaraldehyde in 0.1 mol/L Sörensen phosphate buffer (pH 7.3) for 1 h at 4 °C, post-fixed with 2% osmium tetroxide for 1 h at room temperature, and stained en bloc in 2% uranyl acetate in 30% methanol for 1 h. Following dehydration through a graded ethanol series, cells were embedded in Epon™ 812. Polymerization was complete after 48 h at 60 °C. Ultrathin sections were stained with standard uranyl acetate and lead citrate and observed with FEI Tecnai 12 electron microscope. Digital images were taken with a SIS MegaviewIII CCD camera. Mitochondria length and width were measured using Fiji software^23^. Ratio's length/width closer to 1 indicates that mitochondria are round.

### 2.21 Mitochondrial membrane potential

To analyze the mitochondrial membrane potential, HOS, HOS-R/DOXO and HOS-R/MTX cells were seeded at 110.000 cells/3.5 cm^2^, 190.000 cells/3.5 cm^2^, and 131.000 cells/3.5 cm^2^, respectively, and allowed to adhere for 48 h. Cells were then trypsinized and incubated with 100 nmol/L tetramethylrhodamine methyl ester (TMRM) for 10 min at 37 °C, in the dark. Data acquisition was performed using the BD Accuri™ C6 Plus Flow Cytometer (San Jose, CA, USA). At least 10.000 events were acquired for each sample, and all data were analyzed using FlowJo™ Software (BD Life Sciences).

### 2.22 Mice studies

All *in vivo* experiments were conducted following the approval of the CEEA26 Ethic Committee (approval number: APAFIS #37810-2022062308201583) agreed by the French Ministry of Research regulations (articles R.214-87 à R.214-126). Animals were purchased at Gustave Roussy (Villejuif, France) and conducted in the respective animal facilities following standard animal regulations, health and care, and ethical controls.

#### 2.22.1 *In vivo* dose escalation

For the safety investigation, 25 female NOD.Cg-PrkdcscidIL2rgtm1Wjl/SzJ (NSG) mice were divided blindly and randomly into 5 groups (*n* = 5 per group), each receiving different oral doses of the M30-E05 compound: Control (0 mg/kg); 10, 25, 50, and 100 mg/kg. M30-E05 was freshly prepared and dissolved in 0.5% methyl cellulose prior to each treatment day. Mice were treated for 4 weeks (5 days per week). Behavior, physical appearance, and body weight were monitored and measured to assess any visible potential adverse effects of M30-E05 every day. At the experiment endpoint, blood and different organs (*e.g.*, liver, lung, kidney, spleen, brain and heart) as well as leg bone were collected at the experimental endpoint, fixed in 4% PFA, and processed for histological analysis. Blood was used for plasma collection.

#### 2.22.2 Subcutaneous PDX

2–5 mm soft frozen tumor fragments from GR-OS-18 PDX were first xeno-transplanted for amplification and subsequently transplanted subcutaneously into 35 immunocompromised NSG mice (21 females and 14 males) under anesthesia (3% induction, 2% maintenance isoflurane, and 1.5 L/min air). 35 xenografted mice were randomized into two groups using a blocked randomization approach based on tumor size measurement. Mice were ranked by tumor size and grouped into blocks of two with similar tumor sizes, ensuring each block contained mice of the same sex where possible to balance sex distribution. Within each block, mice were blindly assigned to either the control group or 100 mg/kg M30-E05-treated group. Post-randomization, the mean and standard deviation of tumor sizes were verified and adjusted to be comparable between groups (Control group:mean = 81.66 ± 25.31; M30-E05-treated group: mean = 81.84 ± 29.60). Finally, 17 mice were assigned to the control group, receiving the vehicle only for 5 days per week for two weeks, while 18 mice were assigned to the treatment group, receiving 100 mg/kg M30-E05 on the same schedule. Clinical observations included monitoring skin changes, behavior, posture, responses to handling, and abnormal movements. Body weight and tumor volume were measured twice weekly. Tumor volume was calculated according to the equation: *V* (mm^3^) = width^2^ (mm^2^) × length (mm)/2. The experiments lasted until tumors reached specific endpoints detailed in the ethical projects. Upon euthanasia, blood, tumors, and various organs (bone, liver, lung, kidney, spleen, and brain) were collected for mass spectrometry and Western blot (snap-frozen in liquid nitrogen) and histology analysis. Tissues fixed in 4% PFA and included in FFPE blocs were sectioned and stained with hematoxylin and eosin and analyzed by an experienced histopathologist.

### 2.23 ELISA

Blood from 25 treated mice and control (vehicle-treated) mice used for the *in vivo* efficiency was centrifuged at 400×*g*, 5 min at 4 °C for plasma collection. The mouse cardiac troponin-I (TNNI3) levels in plasma were measured using an ELISA Kit (EEL112, Invitrogen, Courtabœuf, France), following the manufacturer's instructions.

### 2.24 Histology and immunohistochemistry (IHC)

Organs were fixed in 4% PFA and embedded in paraffin; 4 μm sections were stained with hematoxylin-eosin-safranin for morphology. Paraffin sections were processed for heat-induced antigen retrieval (ER2 corresponding EDTA buffer pH 9) for 20 min at 100 °C. Slides were incubated with a mouse monoclonal anti-human Ki67 antibody (1:20, Agilent Dako, clone MIB1) for 1 h at room temperature. The nuclear signal was revealed with the Klear mouse kit (GBI labs). A single representative whole tissue section from each animal was digitized using a slide scanner NanoZoomer 2.0-HT (Hamamatsu Photonics, C9600-13).

### 2.25 Multiplex immunofluorescence analysis

The immunofluorescence multiplex panel was performed using sequential indirect immunolabeling with tyramid signal amplification technique. After tissue dewaxing at 72 °C for 30 s, an antigen retrieval step at pH 9, at 100 °C for 20 min was done with an epitope retrieval solution (ER2). Next, endogenous peroxidases and non-specific binding sites were blocked with hydrogen peroxide (10 min at room temperature) and PKI blocking solution (5 min at room temperature, without wash) prior to the application of the primary antibody for 30 min at RT. This one was then recognized by an anti-rabbit HRP-polymer conjugated secondary antibody or by an ultrapolymer HRP donkey anti-goat incubated for 30 min at RT. The formation of a highly reactive fluorochrome-tyramid complex (Akoya Opal®), able to bind the tyrosin of tissue in the vicinity of the antigen‒antibody complex, was then catalyzed by HRP during an incubation step of 20 min at RT. The antibodies complex (but not the tyramids) were thereafter removed during a stripping step by incubation in ER solution at pH 6, at 97 °C for 20 min, before the next immuno-labeling cycle. Slides were counterstained spectral DAPI (Akoya Biosciences) was added to the slides for 5 min and coverslips were mounted with mounting medium ProLongTM Diamond Antifade Mountant (Invitrogen™, ThermoFisher Scientific).

Multiplex labeling was performed on an automated staining system Leica BOND RX (Leica Biosystems, Nussloch, Germany). The primary antibodies are anti-MTC (Novus Biologicals, NBP2-32980, clone 113-1), anti-AIF (Abcam, ab288370, clone 4B2); anti-CHCHD4 (Proteintech, 21090-1-AP, polyclonal); anti-MICU1 (Abcam, ab190114, goat polyclonal). Tissue sections were sequentially incubated with the following reagents and conditions (dilutions, OPAL) in this order: anti-MTC 1:200, Opal® 620 1:100; anti-AIF 1:500, Opal® 520 1:400; anti-CHCHD4 1:1000, Opal® 520 1:200. Slides were scanned with a Vectra Polaris for analysis. The multiplex immunofluorescence analysis of PDX models was performed as a single experiment without replicates with internal validation controls.

### 2.26 Computational study

The human AIF monomer three-dimensional structure (PDB ID: 1M6I co-crystallized with one flavin adenine dinucleotide was used^25^. Missing residues of the monomer structure (except those at terminal ends) were first added using the SWISS-MODEL webserver^26^. A molecular dynamics (MD) simulation of AIF structure was previously performed in explicit solvent (with 150 mmol/L NaCl) in a cubic box with side 9.3 nm, using GROMACS software^27^ with AMBER99SB-ILDN^28^ and TIP3P^29^ all-atom force fields. A 2-fs integration timestep, and LINCS constraints for h-bonds, were used. Short range cutoff for Coulomb and van der Waals was set to 1.0 nm and particle mesh Ewald was used for long range electrostatics. The system was first equilibrated in the NVT ensemble at 300 K using the V-rescale thermostat (with time constant 0.1 ps) during 1 ns, then in the NPT ensemble at 1 bar using the Parinello-Raman barostat (with time constant 2.0 ps and compressibility 4.5 × 10^−5^ bar^−1^) during another 1 ns. Then, the system was submitted to a 500 ns production run. As can be seen in Supporting Information Fig. S1A, the root-mean-square deviation (RMSD) of the protein as a function of time rapidly reaches a plateau, indicating a stable relaxed AIF three-dimensional structure. Finally, the 5 most populated clusters of the protein conformational ensemble were extracted from the MD trajectory and used as receptors in subsequent docking calculations.

Each studied ligand was blindly docked 100 times on the whole surface of each of the AIF clusters (without specifying any preferred binding site), using AutoDock Vina^30^. Each docking calculation generated 10 binding modes, a total of 5.000 binding modes of each ligand was obtained on the AIF protein. In addition, the docking results of M30-E05 on human AIF were compared to those obtained on murine AIF (PDB ID: 1GV4)^31^ using a similar protocol. The best binding mode of M30-E05 on human AIF was considered as an initial structure of the complex and subsequently submitted to a 1 μs MD simulation to assess its stability. We used the same simulation conditions and parameters as for the unbound protein, to which we added the GAFF force field for the M30-E05 ligand^32^.

### 2.27 Statistics

Statistical analysis was performed using GraphPad Prism 10.0. Mann-Whitney U test was applied for RNA-Seq analysis, and IHC analysis of AIF, Ki-67 expression in tumor: *P* < 0.033 (∗), *P* < 0.002 (∗∗), *P* < 0.001 (∗∗∗). One-way ANOVA test was used for statistical analysis of biological experiments: *P* < 0.05 (∗), *P* < 0.01 (∗∗), *P* < 0.001 (∗∗∗), *P* < 0.0001 (∗∗∗∗). Two-way ANOVA (mixed model) was used for tumor volume curves: *P* > 0.1234 (ns, not significant), *P* < 0.0332 (∗), *P* < 0.0021 (∗∗), *P* < 0.0002 (∗∗∗), *P* < 0.0001 (∗∗∗∗).

## 3 Results

### 3.1 Identification and synthesis of M30-E05 as an AIF/CHCHD4 inhibitor

**Figure 1:**
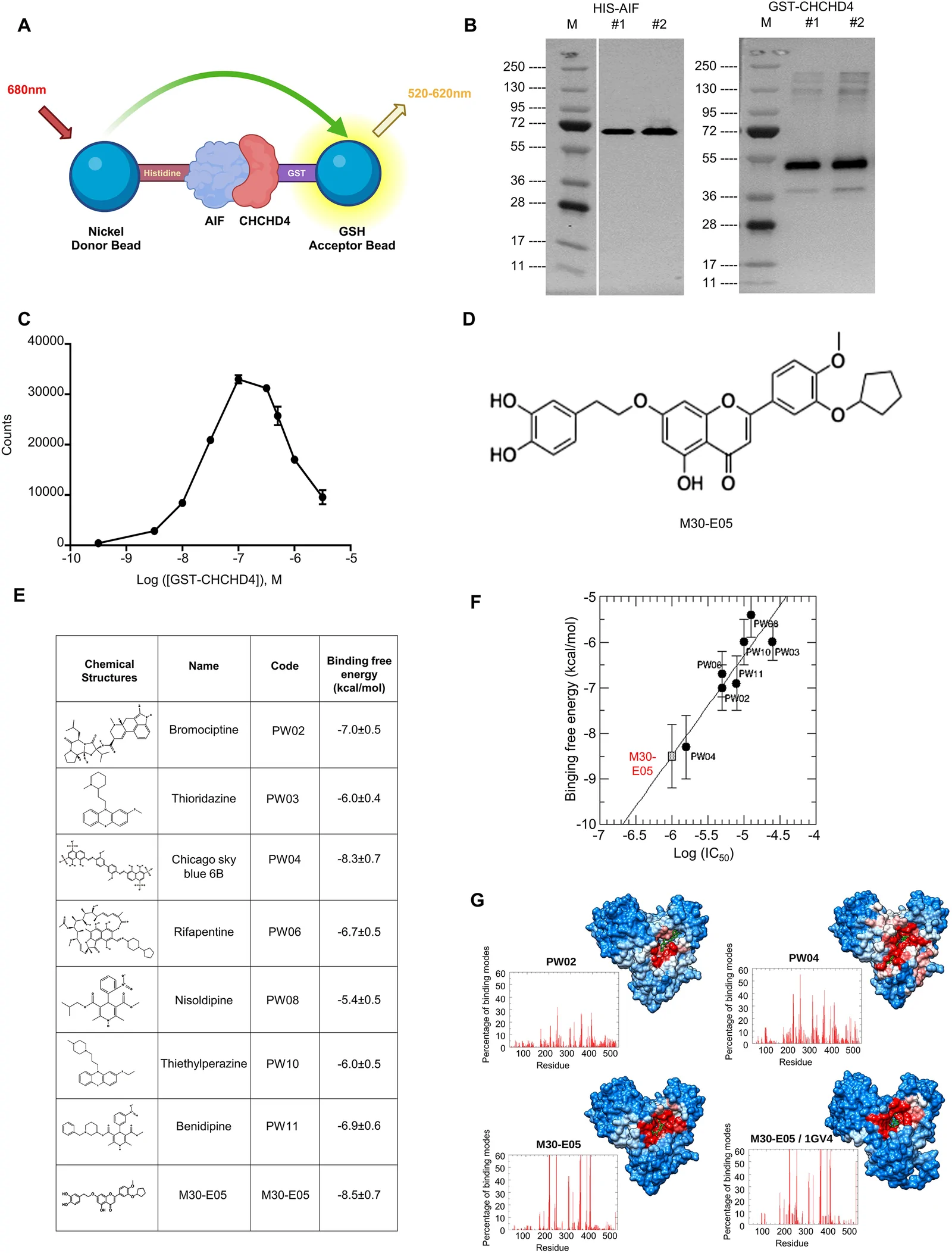
High throughput screening assay for the robotized screening of protein‒protein interaction inhibitor of apoptosis-inducing factor (AIF)-coiled-coil-helix-coiled-coil-helix Domain Containing 4 (CHCHD4) complex and M30-E05 synthesis. (A) Scheme of the *in vitro* Alpha screening assay to measure the interaction between HIS-AIF_103-613_ and GST-CHCHD4 coupled to donor and acceptor beads, adapted from manufacturer's protocol. (B) Human AIF_103-613_ and CHCHD4 were produced as recombinant proteins in *E. coli* and their purity was analyzed by SDS-PAGE and Western-blot. #, independent production and purification experiments. (C) Optimization of the ratio AIF/CHCHD4 coated on beads for the detection of their interaction. (D) Chemical formula of M30-E05. (E) Chemical structure of experimentally identified inhibitors of AIF/CHCHD4 interaction and binding free energy for human AIF estimated by Autodock Vina docking calculations. PW, Prestwick. (F) Correlation between AutoDock Vina binding free energies of 7 Prestwick compounds (black circle) on human AIF and their log(IC_50_) experimentally measured. The red square on the correlation line indicates M30-E05 binding free energy. (G) Contact frequency of AIF residues with PW02, PW04, and M30-E05 binding modes (graphs) and localization of AIF surface residues most frequently in contact (blue, white and red colors indicate residues that are contacted less than 10%, between 10% and 20%, and more than 20% out of all the ligand binding modes, respectively). The best binding mode of each ligand is highlighted in green. The bottom-right graph and structure are the results of M30-E05 docking on murine AIF.

AIF and CHCHD4 associate in a protein‒protein complex that is necessary for the biogenesis of respiratory chain complexes, different mitochondrial activities, and cell survival^19^. A robotized high-throughput screening assay to identify compounds interrupting the interaction between AIF and CHCHD4 has been developed using the Alphascreen technology^18^ (Fig. 1A). In this assay, we produced tagged HIS-AIF_103-613_, and GST-CHCHD4 recombinant proteins in *E. coli*^18^ and purified them to homogeneity as shown by Western blot (Fig. 1B). The proteins were subsequently coupled with donor and receptor beads using their tags, and the optimal concentrations of each protein were optimized for the following measurement of their interaction by light emission in 384-well plates (Revvity) (Fig. 1C). As a positive control of protein‒protein interaction inhibitor capable of fully interrupting the AIF/CHCHD4 interaction, a synthetic peptide, called N27, corresponding to the N-terminal 27 amino acids of CHCHD4 was used^19^. In a pilot experiment, the miniaturization and robotization of the assay was validated by a z’ factor ranging from 0.5 to 1. Subsequently, we conducted a screening campaign using the Prestwick library of 1.280 FDA-approved molecules, a library of 2.901 new compounds from the French National Chemical library, and 99 newly synthesized molecules from the BioCIS laboratory. Compounds (tested at 10 μmol/L, *n* = 1) were evaluated for their capacity to reduce the alpha signal, compared to N27 as a positive control. Consequently, we identified 11 hits coming from the Prestwick library^18^ and 4 from the BioCIS laboratories. Among a total of 131 screened flavonoids, M30-E05 interrupted the AIF/CHCHD4 complex with the best activity. Moreover, as it was easily synthesizable at the multigram scale, we decided to pursue the characterization of its bioactivity in an anti-cancer therapeutic perspective (Fig. 1D).

Next, M30-E05 was resynthesized through a 7-step strategy at multigram scale (Supporting Information, patent PCT/EP2025/077498). The synthesis started from diosmetin (**1**), which was selectively alkylated in 3 steps with a cyclopentyl group at C-3′ yielding flavone (**2**). A benzyl protection of the most reactive C-7 hydroxy group was necessary to achieve this synthesis. In parallel, the synthesis of iodide (**4**) was achieved by protection of catechol (**3**) as an orthoester, followed by a Finkelstein reaction. The alkylation of the most reactive C-7 hydroxy group of (**2**) with (**4**) yielded the expected coupling product. Subsequent hydrogenation with catalytic HCl, directly yielded M30-E05, by selectively reducing the aromatic ketone and deprotecting the orthoester function simultaneously.

### 3.2 In silico characterization of M30-E05 binding free energy and binding site on AIF

We first used the docking procedure described in Section 2 on 7 compounds from the Prestwick chemical library (Fig. 1E) for which an experimental IC_50_ of the AIF/CHCHD4 interactions could be measured by the Alphascreen technology^18^. As shown in Fig. 1F, ligand average free binding energies yielded by AutoDock Vina on AIF monomer could be fairly correlated with the experimental log(IC_50_), indicating that our computational method is a helpful tool for identifying potential efficient inhibitors of AIF/CHCHD4 interactions (Fig. 1F).

In a second step, the M30-E05 compound (Fig. 1E), which interrupts the AIF/CHCHD4 complex, was docked on the AIF monomer using the same protocol. Our calculations show that M30-E05 has a binding free energy estimation of −8.5 ± 0.7 kcal/mol, which is better than those of the 7 previous Prestwick compounds (Fig. 1F). From the best docking binding mode of M30-E05 to AIF, a 1 μs MD simulation of the complex was performed to verify its stability. As shown in Fig. S1A, the RMSD of the complex, the protein, and of the ligand in the binding pocket (with respect to the initial structure) have all reached a plateau, indicating a convergence of the AIF-M30-E05 quaternary structure toward an equilibrium. It could be noted that the rather low value of the protein RMSD plateau (0.3 nm) suggests that the ligand does not induce significant global conformational changes within a timescale of 1 μs (Fig. S1A).

To gain insight into the mechanism of action of these AIF/CHCHD4 complex inhibitors, we further investigate the preferred binding site of the two best hits from Prestwick, PW02, PW04, and M30-E05 molecules on the AIF monomer. Strikingly, as shown in Fig. 1G, these molecules preferentially bind the same surface area on the AIF protein, which corresponds to the NAD binding pocket. For the best docking binding mode of M30-E05 to AIF, amino acid residues that interact with M30-E05 are described (Fig. S1B). In comparison to the PW03 compound, which exhibits a lower binding affinity for AIF, the two M30-E05 terminal moieties establish additional interactions with the hydrophobic amino acids A374, I375, G376, and V378, as well as with the polar groups of residues P144 and R165. This is in addition to the AIF residues involved in binding both ligands (the apolar F190, L191, and I223, and the charged R331 and E333) (Fig. S1B).

Finally, we performed a similar docking procedure of M30-E05 on the murine AIF structure (PDB ID: 1GV4). The obtained average binding free energy is −9.0 ± 0.7 kcal/mol, which is slightly lower than that of the human protein. Furthermore, the preferential binding site of M30-E05 on murine AIF is also found to be the NAD binding pocket (Fig. 1G). Altogether, these data strongly suggest that M30-E05 might competitively inhibit NAD binding, which in turn would prevent AIF dimerization.

### 3.3 Cellular and molecular mechanisms of action of M30-E05

**Figure 2:**
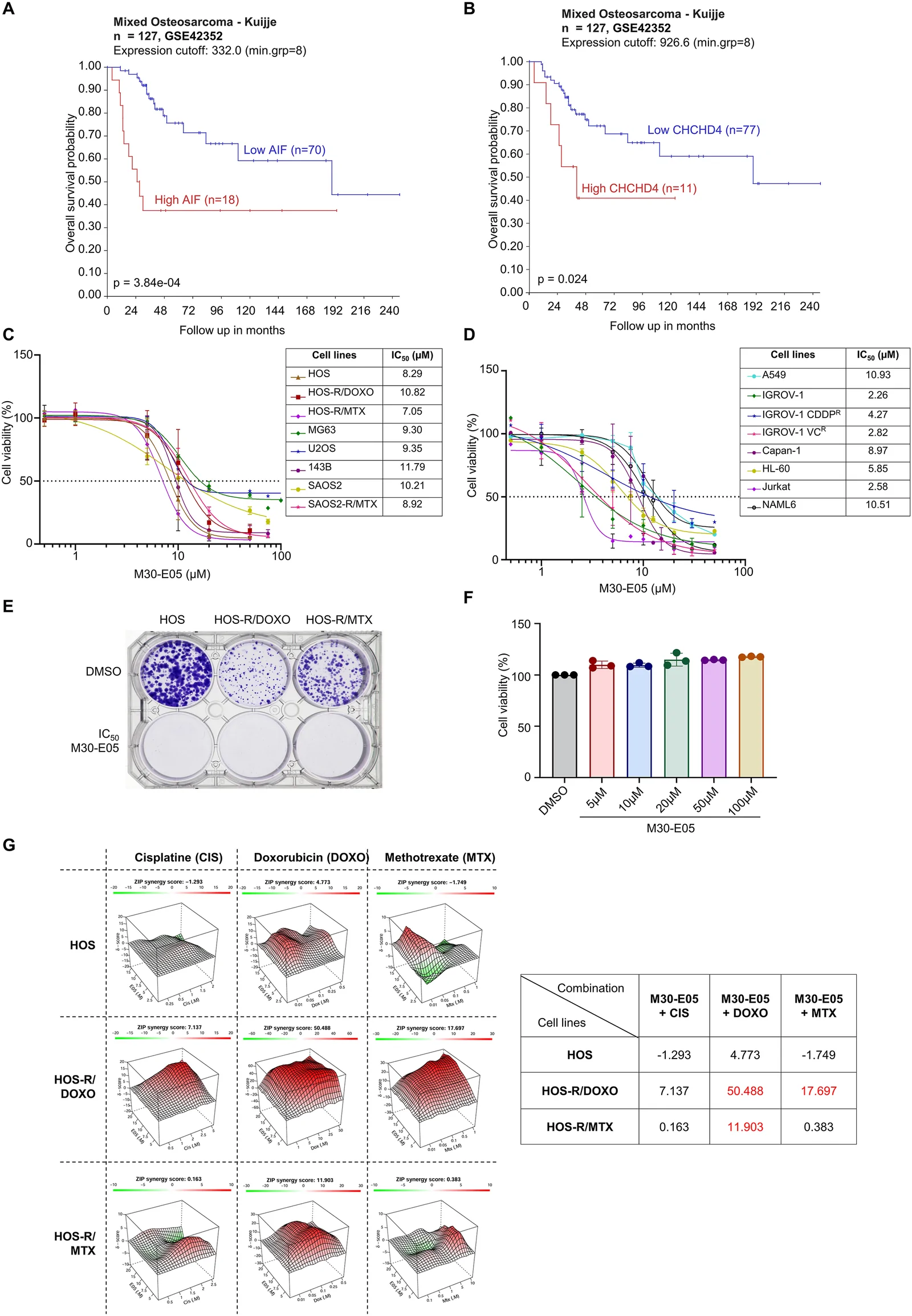
M30-E05 exhibits cytotoxicity on different cancer cell lines. (A, B) Kaplan‒Meier overall survival analysis for patients with osteosarcoma in the GSE42352 dataset. Kaplan‒Meier curves for (A) *AIFM1* gene and (B) *CHCHD4* gene of low-expression versus high-expression groups based on median *AIFM1* and *CHCHD4* expression, respectively. Kaplan-Meier curves were generated using R2: Genomics Analysis and Visualization Platform. (C) Cell viability of 8 pediatric osteosarcoma cell lines treated with M30-E05. Dose‒response curves represent cell viability measured using a lactate dehydrogenase (LDH) assay at increasing concentrations of M30-E05 after 72 h of treatment. Individual experiments are shown *(n* > 2). Data were shown as mean ± SD. IC_50_, inhibitory concentration yielding to 50% cell viability. 0.002% DMSO was used as the solvent of M30-E05 and as a control for calculation of percentage of viability. (D) Cell viability of 10 adult cancer cell lines treated with M30-E05. Dose-response curves represent cell viability measured using a LDH assay at increasing concentrations of M30-E05 after 72 h of treatment. Individual experiments are shown (*n* > 2). Data were shown as mean ± SD. 0.002% DMSO was used as the solvent of M30-E05 and as a control for calculation of percentage of viability. (E) Image of a 6-well plate showing fixed colonies subjected to crystal violet staining of HOS parental (HOS), HOS-doxorubicin resistant (HOS-R/DOXO), and HOS-methotrexate resistant (HOS-R/MTX) cells after 10 days of culture, treated with either 0.002% DMSO as a negative control or IC_50_ M30-E05. (F) Cell viability of peripheral blood mononuclear cells (PBMC) isolated from human donors treated with either 0.002% DMSO as a negative control or with M30-E05 at various concentrations (5, 10, 20, 50, and 100 μmol/L), assessed *via* LDH assay after 72 h. Individual experiments are shown (*n* = 3). Data were shown as mean ± SD. (G) Illustrative 3D synergy map of M30-E05 in combination with CIS, DOXO, MTX at depicted concentrations in HOS, HOS-R/DOXO, HOS-R/MTX cell lines obtained using SynergyFinder tool (https://synergyfinder.fimm.fi/).

To explore the role of AIF and CHCHD4 in osteosarcoma, we thus assessed their mRNA expression levels, using the GSE42352 dataset from the publicly available R2 Genomics Analysis and Visualization Platform (https://hgserver1.amc.nl/cgi-bin/r2/main.cgi) ^33^. Kaplan‒Meier analysis revealed that higher *AIFM1* and *CHCHD4* mRNA expressions correlate with decreased overall survival in osteosarcoma samples, suggesting an adverse prognostic impact (Fig. 2A and B). In the RNA-seq transcriptomic analysis of patient cohorts from the MAPPYACTS^34^ and OS 2006^35^ trials, including patients at relapse and diagnosis, significant levels of *AIFM1* and *CHCHD4* were observed (Supporting Information Fig. S2A and S2B), which aligned with the transcriptomic analysis of several corresponding osteosarcoma PDX models (Fig. S2C). In addition, none of the osteosarcoma patients harbored mutations of the *AIFM1* gene. Only 2 out of 628 patients of the MAPPYACTS cohort (one Ewing sarcoma patient and one brain central nervous system patient) presented somatic *AIFM1* gene mutations^34^. These findings confirmed osteosarcoma as a suitable model for evaluating the efficacy of the AIF/CHCHD4 complex inhibitor.

To assess the cytotoxicity of M30-E05, a panel of osteosarcoma cell lines, including both parental and drug-resistant cell lines (HOS, HOS-R/DOXO, HOS-R/MTX, MG63, U2OS, 143B, SAOS2, and SAOS-R/MTX), was treated with M30-E05 (8 doses, ranging from 1 to 100 μmol/L dissolved in 0.002% DMSO) for 72 h. Cell viability was then evaluated using a LDH assay, and the IC_50_ of M30-E05 was determined using GraphPad Prism software. Across all tested cell lines, the IC_50_ was approximately 10 μmol/L (HOS at 8.29 μmol/L), regardless of their resistance to doxorubicin (HOS-R/DOXO at 10.82 μmol/L) and methotrexate (HOS-R/MTX at 7.05 μmol/L) (Fig. 2C). M30-E05 treatment also resulted in reduced viability of several tumor cell lines derived from ovarian cancer (both parental and drug-resistant cell lines), lung cancer, pancreatic cancer, as well as leukemia (Fig. 2D). Notably, M30-E05 exhibited an IC_50_ of 2‒3 μmol/L in ovarian cancer and some leukemia cell lines.

To determine whether different cell line profiles influenced the effects of M30-E05, we characterized in detail three human osteosarcoma cell lines: HOS, the parental cell line, and two derived drug-resistant lines, HOS-R/DOXO and HOS-R/MTX, obtained by culture in sublethal doses of doxorubicin (DOXO) and methotrexate (MTX)^20^. In the HOS-R/DOXO cell line, the mechanism of resistance has been described as an increase in permeability-glycoprotein efflux activity, while HOS-R/MTX exhibited a decrease in reduced folate carrier (RFC)/solute carrier family 19 member 1 (SCL19A1) transporter activity and an up-regulation of dihydrofolate reductase (DHFR)^20^,^36^. Moreover, immunoblot analysis confirmed the expression of AIF and CHCHD4 protein in all three cell lines. Of note, AIF protein levels were reduced in HOS-R/DOXO, whereas CHCHD4 protein expression was elevated in HOS-R/MTX, compared to its parental cell line (Fig. S2D). Immunofluorescence and electron microscopy revealed a fragmented mitochondrial network in HOS-R/DOXO and HOS-R/MTX cells, compared to HOS (Supporting Information Fig. S3A and S3B). In addition, compared to HOS, HOS-R/DOXO cells exhibited a reduction in mitochondrial transmembrane potential, mitochondrial OCR, and total ATP content (Fig. S3C‒S3E). On the other hand, HOS-R/MTX displayed close-to-normal mitochondrial potential and respiration rate and showed a subnormal ATP content (Fig. S3C‒S3E). Both resistant cell lines manifested a shift in metabolite abundance towards lipid and amino acid metabolism compared to the parental HOS cell line (Fig. S3F and S3G). Although the three cell lines exhibited distinct resistance mechanisms and, cellular and molecular profiles, they shared similar IC_50_ values of M30-E05 (approximately 10 μmol/L). Moreover, in a clonogenic assay conducted on these three cell lines, all cells undistinguishably succumbed to treatment with their respective IC_50_ of M30-E05 within 10 days (Fig. 2E). Altogether, these observations suggest that M30-E05 could overcome DOXO and MTX resistance.

Moreover, in the parental model HOS, while combining M30-E05 with three conventional chemotherapeutics (CIS, DOXO, MTX) used in clinics for OS treatment, HOS cells only demonstrated the additivity effect. Interestingly, M30-E05 showed the highest ZIP synergy score (50.488) with DOXO when treated in HOS-R/DOX, which indicates that our molecule could resensitize HOS-R/DOXO to DOXO. In addition, synergism was found between M30-E05 and MTX in HOS-R/DOXO (17.697), and between M30-E05 and DOXO in HOS-R/MTX (11.903). These results highlight that M30-E05 is a valuable candidate for incorporation into the treatment regimen for resistant OS, showing synergy with chemotherapies.

Thus, we next performed silencing of AIF in HOS cells using a suitable siRNA to evaluate the specificity of M30-E05 in targeting AIF. The compound showed two times less efficacy in AIF knocked-down HOS (IC_50_ = 16.52 μmol/L), compared to its control (Fig. S2E). Importantly, M30-E05 treatment from 5 up to 100 μmol/L did not affect the viability of freshly isolated human PBMC (Fig. 2F), while the IC_50_ of M30-E05 for non-cancerous HEK293 cells was ∼62 μmol/L (Fig. S2E).

**Figure 3:**
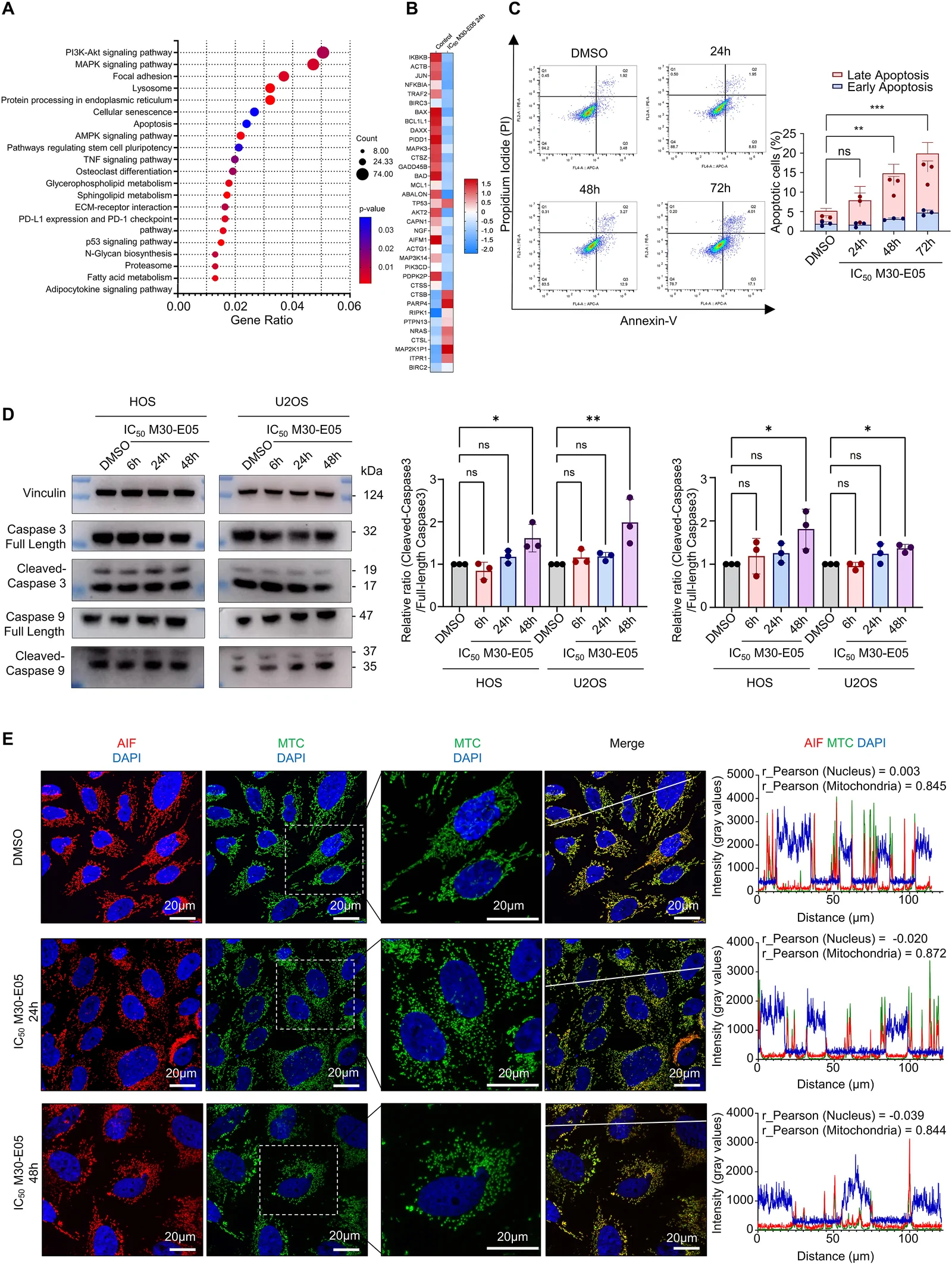
M30-E05 induces AIF-independent apoptosis and caspase activations in HOS cell line. (A) Scatter plot exhibiting the most enriched Gene Ontology (GO) biological process results from all differentially expressed genes. (B) Heat map of apoptosis-related genes that are differentially expressed based on z-score normalized pseudocounts. (C) Annexin V/PI double-staining assay of HOS cells showing flow cytometric analysis of IC_50_ (8.3 μmol/L) M30-E05-induced apoptosis at different time points (24, 48, and 72 h). 0.002% DMSO was used to treat HOS cells and considered as a negative control. Statistical graph of Annexin V-FITC/PI staining results, with data representing the mean of three independent experiments. Annexin V+/PI− cells are early apoptotic, and Annexin V+/PI + cells are late apoptotic. Data were shown as mean ± SD and statistical significance was assessed using one-way ANOVA with Šídák's multiple comparisons test. Significance levels are indicated as follows: not significant (ns), *P* < 0.0332 (∗), *P* < 0.0021 (∗∗), *P* < 0.0002 (∗∗∗), and *P* < 0.0001 (∗∗∗∗). (D) Western Blot analysis showing the expression levels of full-length Caspase 3 and Caspase 9 and their cleaved forms in response to either 0.002% DMSO as a negative control or IC_50_ (8.3 μmol/L) of M30-E05 treatment over time (6, 24, and 48 h). Vinculin was used as loading controls to normalize. Normalized data were used to calculate cleaved-caspase 3/full-length caspase 3 and cleaved-caspase 9/full-length caspase 9 ratio. Data are presented as the mean ± SD from four independent experiments (*n* = 3). Statistical significance was assessed using ANOVA with Sidak's correction for multiple comparisons to compare between treated *versus* non-treated condition. Significance levels are indicated as follows: not significant (ns), *P* < 0.0332 (∗), *P* < 0.0021 (∗∗), *P* < 0.0002 (∗∗∗), and *P* < 0.0001 (∗∗∗∗). (E) Representative confocal images of HOS cells exposed to either 0.002% DMSO as a negative control or IC_50_ (8.3 μmol/L) M30-E05 for 0, 24 and 48 h. AIF colocalization in mitochondria labelled with MTC and in the nucleus stained with DAPI is evaluated. Intensity profiles of AIF, MTC and DAPI, following a defined line in X, and its superposition is analyzed in Z-stack projections of each condition and are represented in graphs on the right. The line was chosen to contain the signal of at least three nuclei. Four independent experiments were performed. The Pearson coefficients (r_Pearson) were calculated and represented as mean in each image, from four independent experiments, using the JaCoP plugin in Fiji Software.

To investigate in depth the impact of M30-E05 on the transcriptome, we performed bulk RNA-sequencing analysis of M30-E05-treated HOS cells at 24 h and the corresponding untreated condition. GO analysis of M30-E05-treated HOS cells revealed different enriched pathways, among which apoptosis was significantly represented (*P*-value = 0.0372) (Fig. 3A). In addition, a detailed heatmap of deregulated genes involved in the apoptosis gene set showed increases in some pro-apoptotic genes, including tumor suppressor TP53, PARP4 and ITPR1, as well as decreases in some anti-apoptotic genes such as *BCL1L1* (BCL-XL), *MCL1* (MCL1) (Fig. 3B). Based on previously mentioned transcriptomic changes in the apoptosis pathway, we thus measured apoptotic rate using Annexin V/PI staining and cytofluorometric analysis at different time points after treating HOS cells with its IC_50_ (8.29 μmol/L). Significant induction of apoptosis by M30-E05 in HOS cells was observed starting from 48 h with an increase in both early (Annexin V+/PI‒) and late (Annexin V+/PI+) apoptotic populations (Fig. 3C). Furthermore, to evaluate the activation of caspases during apoptosis, the ratios of cleaved-caspase 3/full-length caspase 3 and cleaved-caspase 9/full-length caspase 9 were determined in HOS and U2OS cells treated with M30-E05 at different time points (6, 24, 48 h) and compared with their corresponding untreated control. In the presence of M30-E05, at 48 h, both ratios exhibited a 1.5- to 2-fold increase relative to untreated cells, further supporting apoptosis activation (Fig. 3D). As AIF has been described to play dual roles in survival or cell death *via* nuclear translocation and DNA fragmentation, we next examined its subcellular location by confocal immunofluorescence microscopy. Although M30-E05 induced the fragmentation of the mitochondrial network and the relocalization of mitochondria towards the nuclear envelope without any chromatin condensation at 24 and 48 h (Fig. 3E), AIF remained localized at mitochondria (r_Pearson = 0.872 at 24 h and r_Pearson = 0.844 at 48 h post M30-E05 treatment) and failed to translocate to nuclei (r_Pearson = −0.020 at 24 h and r_Pearson = −0.039 at 48 h post M30-E05 treatment) (Fig. 3E). Altogether, our findings revealed that caspase activation-mediated, AIF-independent apoptosis contributes to M30-E05 mechanism of action in OS cells.

**Figure 4:**
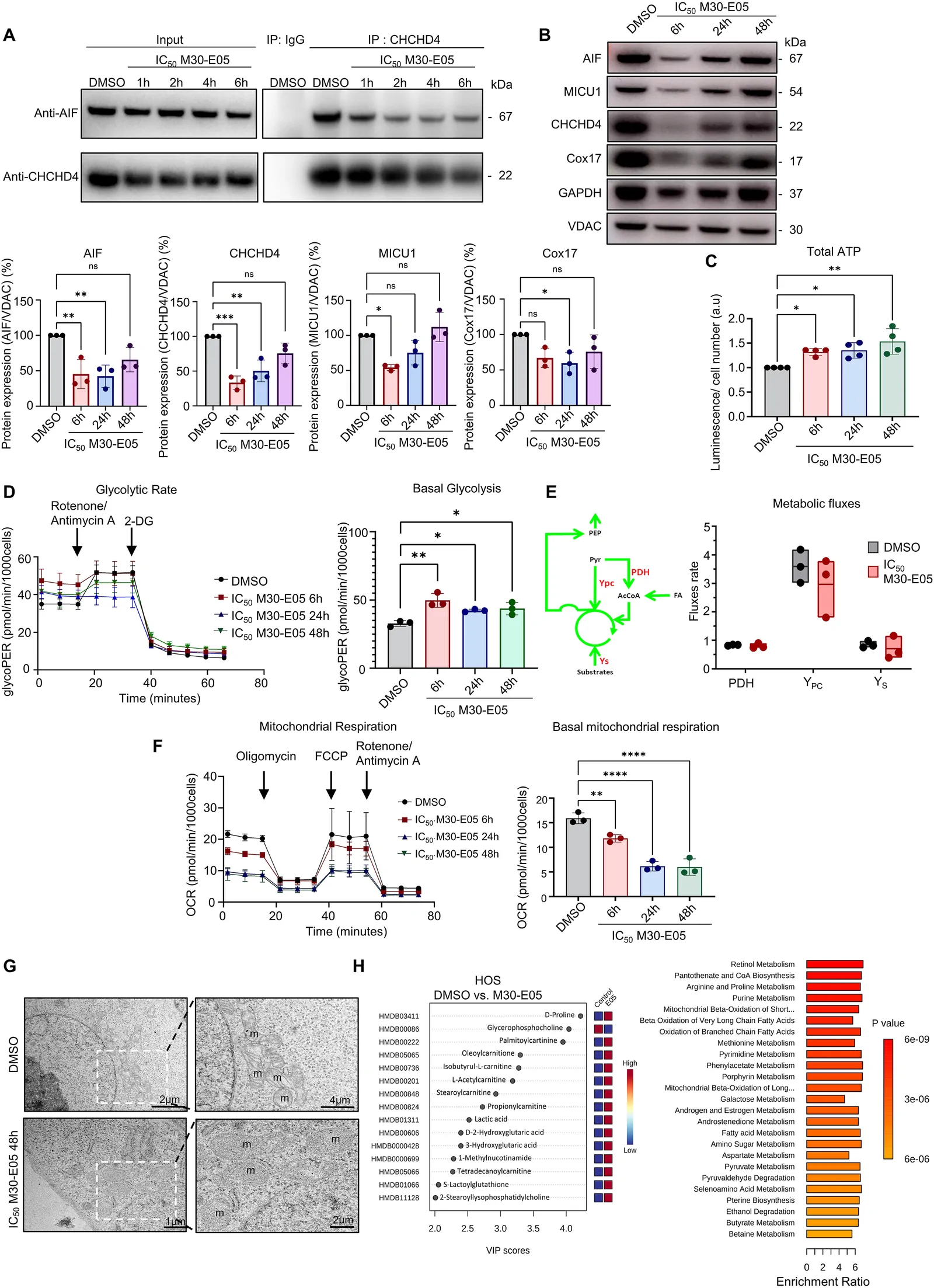
Effects of M30-E05 on energetic metabolism, mitochondria and metabolic reprogramming in osteosarcoma cell lines. (A) AIF/CHCHD4 co-immunoprecipitation using CHCHD4 antibody to immunoprecipitate whole protein fraction from HOS cells treated with DMSO or with M30-E05 during 1, 2, 4, 6 h. Immunoprecipitation was followed by Western blotting with AIF and CHCHD4 immunostaining. IgG immunoprecipitated sample was used as negative control. (B) Western Blot analysis showing the expression levels of AIF, CHCHD4 and their substrates Cox17 and MICU1 in response to either 0.002% DMSO as a negative control or IC_50_ (8.3 μmol/L) of M30-E05 treatment over time (6, 24, and 48 h). Vinculin and VDAC were used as loading controls. Data are presented as the mean ± SD from three independent experiments (*n* = 3). Data were shown as mean ± SD and statistical significance was assessed using ANOVA with Sidak's correction for multiple comparisons. Significance levels are indicated as follows: not significant (ns), *P* < 0.0332 (∗), *P* < 0.0021 (∗∗), *P* < 0.0002 (∗∗∗), and *P* < 0.0001 (∗∗∗∗). (C) ATP levels were semi-quantified over time in HOS cells pre-treated with either 0.002% DMSO as a negative control or with IC_50_ (8.3 μmol/L) M30-E05 for 6, 24, and 48 h, using the ATPlite assay. Data were shown as mean ± SD and statistical analysis was performed using ANOVA with Sidak's correction for multiple comparisons, with significance levels indicated as follows: ns, not significant; *P* < 0.0332 (∗); *P* < 0.0021 (∗∗); *P* < 0.0002 (∗∗∗); *P* < 0.0001 (∗∗∗∗). (D) Seahorse XFe96 Glycolytic Rate Test graph displaying the proton efflux rate (glycoPER) value (pmol H^+^/min/1000 cells) over time in HOS cells pre-treated with either 0.002% DMSO as a negative control or with IC_50_ (8.3 μmol/L) M30-E05 for 6, 24, and 48 h (left). The test was conducted using the following compounds: Rotenone/Antimycin A (0.5 μmol/L) and 2-deoxy-d-glucose (50 mmol/L). Basal glycolysis values were extrapolated from the kinetic graph. glycoPER measurements were normalized to cell counts determined by nuclei DAPI staining. Data represent the mean ± SD with at least six technical replicates. Statistical analysis was performed using ANOVA with Sidak's correction for multiple comparisons, with significance levels indicated as follows: ns, not significant; *P* < 0.0332 (∗); *P* < 0.0021 (∗∗); *P* < 0.0002 (∗∗∗); *P* < 0.0001 (∗∗∗∗). (E) tcaCALC metabolic modelling of HOS cells following administration of [U-^13^C]glucose with either vehicle (DMSO) (*n* = 3) or M30-E05 administered at the IC_50_ dose (*n* = 3). Left panel indicates the model that provided the best fit of the glutamate ^13^C-isotopomer data where the parameters include the fraction of acetyl CoA derived from pyruvate *via* PDH, anaplerotic pyruvate carboxylase flux relative to that of citrate synthase (Y_PC_) and anaplerotic fluxes from unlabeled non-pyruvate substrates relative to that of citrate synthase (Y_S_). Right panel indicates the estimated fluxes of pyruvate dehydrogenase (PDH), Y_PC_ and Y_S_ for the two conditions. Statistical analysis was performed using multiple Unpaired *t*-test with Welch correction (*α* = 0.95), with false discovery rate (FDR) correction for multiple comparisons. (F) Seahorse XFe96 Mito Stress Test graph displaying the oxygen consumption rate (OCR) (pmol/min/1.000 cells) over time in HOS cells pre-treated with either 0.002% DMSO as a negative control or with IC_50_ (8.3 μmol/L) M30-E05 for 6, 24, and 48 h (left). The test was conducted using the following compounds: Oligomycin (2.5 μmol/L), FCCP (1 μmol/L), and rotenone/antimycin A (0.5 μmol/L). Basal respiration values were extrapolated from the kinetic graph. OCR measurements were normalized to cell counts determined by nuclei DAPI staining. Data represent the mean ± SD of three independent experiments, each with at least six technical replicates. Statistical analysis was performed using ANOVA with Sidak's correction for multiple comparisons, with significance levels indicated as follows: ns, not significant; *P* < 0.0332 (∗); *P* < 0.0021 (∗∗); *P* < 0.0002 (∗∗∗); *P* < 0.0001 (∗∗∗∗). (G) Representative transmission electron microscopy images of HOS cells treated with either 0.002% DMSO as a negative control or with IC_50_ (8.3 μmol/L) M30-E05 for 48 h, showing that the compound induced cristolysis and changes in mitochondrial ultrastructure. m: mitochondrion. (H) Left panel: VIP (variable importance in projection) score plot depicting metabolites that significantly differentiate between 0.002% DMSO as a negative control and IC_50_ (8.3 μmol/L) M30-E05-treated HOS cell lines following 72 h of treatment. Two vertical box plots on the right illustrate metabolic variations in 0.002% DMSO *versus* M30-E05-treated HOS cells. Right panel: Enrichment analysis of metabolites that differ significantly between 0.002% DMSO as a negative control and IC_50_ (8.3 mmol/L) M30-E05-treated HOS cells following 72 h of treatment. The enrichment ratio represents the observed number of metabolites in a specific metabolic pathway divided by the expected number. Metabolic pathways are ranked by *P*-value, with the most significant pathways at the top.

Moreover, the co-immunoprecipitation experiment revealed the disruption of AIF/CHCHD4 interaction upon M30-E05 treatment from 1 h and increasing over time until 6 h of treatment (Fig. 4A). Coherent with this result, the immunoblot-detectable protein expression of AIF and CHCHD4 revealed an early depletion of both proteins within 6 h of treatment with the IC_50_ (8.29 μmol/L) of M30-E05 in HOS cells (Fig. 4B). Expression of VDAC, a mitochondrial outer membrane protein independent of AIF/CHCHD4, remained unchanged under treatment conditions, indicating that the mitochondrial mass is unaffected. Moreover, the protein expression of two substrates of the AIF/CHCHD4 pathway, MICU1 and COX17 also decreased, indicating that the interruption of AIF/CHCHD4 complex might perturb mitochondrial respiratory chain biogenesis and mitochondrial calcium homeostasis.

Next, to decipher how M30-E05 impacts energetic metabolism, total cellular ATP levels were measured, which surprisingly increased starting from 6 h (Fig. 4C). This observation was explained by the increase in glycoPER value (Fig. 4D), accompanied by the decrease in OCR value starting at 6 h post-treatment, with a maximal effect at 48 h (Fig. 4F). Together, M30-E05 induces a metabolic shift toward glycolysis, elevating ATP production despite reduced mitochondrial oxidative activity. To better understand the contribution of the tricarboxylic acid cycle (TCA) to mitochondrial respiration in response to M30-E05 treatment, we thus evaluated its activity by using ^13^C NMR tracing combined with mass-isotopomeric flux analysis. Based on the ^13^C NMR tracing data and tcaCALC modeling, we identified the model with the smallest sum of squared residual errors between experimental and simulated glutamate ^13^C-isotopomer data, along with the most favorable Akaike Information Criterion (AIC) across all datasets (Fig. 4D). This model provided estimates of the fraction of acetyl-CoA derived from pyruvate *via* pyruvate dehydrogenase (PDH) and distinguished anaplerotic fluxes through pyruvate carboxylase (Y_PC_) from those of non-pyruvate substrates such as glutamine (Y_S_). According to the results, PDH fluxes approached 1.0, indicating that pyruvate was the predominant source of acetyl-CoA to the TCA cycle. Anaplerotic fluxes were also active and primarily dominated by Y_PC_, whose flux was 3‒4 times that of other anaplerotic precursors such as glutamine (Fig. 4E). Treatment with M30-E05 did not significantly modify this flux profile in these cell cultures, nor did it alter the flux profiles of HOS-R/DOXO and HOS-R/MTX cells (Supporting Information Fig. S4A and S4B). These findings indicate that while M30-E05 caused significant decreases in OCR–a proxy for absolute oxidative TCA cycle flux, did not modify substrate selection for acetyl-CoA and anaplerotic pathways, and did not alter the relative rates of anaplerotic to oxidative TCA cycle fluxes. In line with these functional defects, transmission electron microscopy showed morphological alterations of mitochondria, including a rounder shape, increased matrix density and cristae loss in M30-E05-treated HOS cells (Fig. 4G). Finally, GC‒MS/MS metabolomic analysis demonstrated profound changes in HOS cellular metabolome, which highlighted the significant impact of M30-E05 on mitochondrial *β*-oxidation of short- and long-chain fatty acids, as well as metabolic pathways affecting amino acids including arginine and proline (Fig. 4H). Similar metabolic reprogramming in energy substrate use was also observed in two other resistant HOS-derived cell lines (HOS-R/DOXO, HOS-R/MTX) (Fig. S4C and S4D).

### 3.4 M30-E05 effects on secondary cultures of osteosarcoma PDX

**Figure 5:**
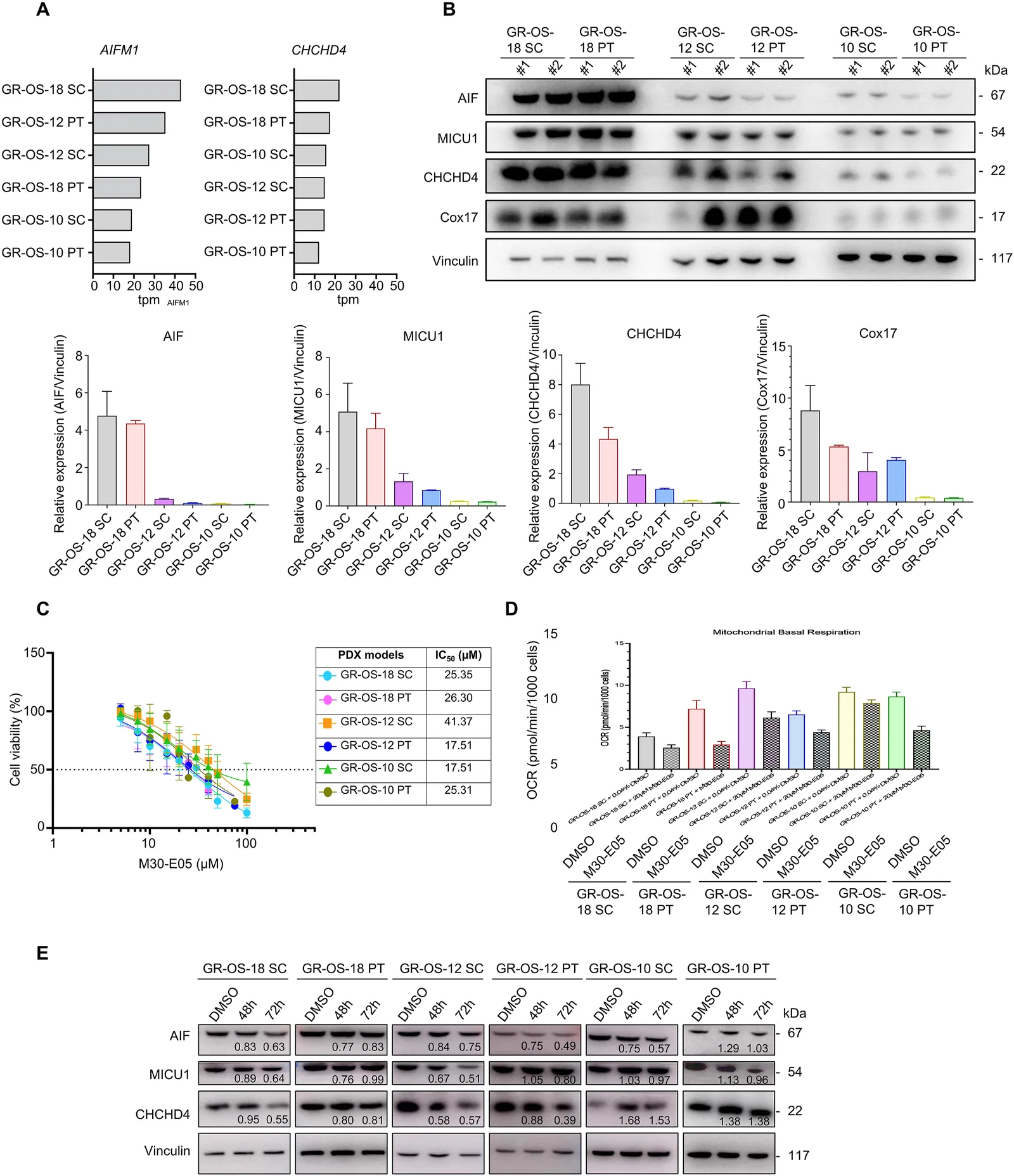
Characterization of AIF/CHCHD4 complex and cellular responses in secondary cultured *in vitro* patient-derived xenografts (PDX) following M30-E05 treatment. (A) Normalized gene expression levels of *AIFM1* and *CHCHD4* from RNA-seq data across six different PDX samples, ranked from highest to lowest expression. Tpm, transcripts per million. (B) Representative Western blot analysis showing the expression levels of the AIF, CHCHD4 and its substrates (MICU1) across six different secondary cultured *in vitro* PDX samples with 2 biological replicates (#1 and #2 for each PDX). Vinculin was used as a loading control. Experiments have been repeated twice. (C) Cell viability of 6 different secondary cultured *in vitro* PDX samples treated with different concentrations of M30-E05, was measured using an LDH assay. Dose‒response curves represent cell viability at increasing concentrations of M30-E05 after 72 h of treatment. Individual replicates are shown (*n* > 2). (D) Basal respiration displaying the OCR values (pmol/min/1.000 cells) in 6 different secondary cultured *in vitro* PDX samples pre-treated with either 0.04% DMSO as a negative control or with 20 μmol/L M30-E05 for 24 h. Seahorse XFe96 MitoStress Test was conducted using the following compounds: Oligomycin (2.5 μmol/L), FCCP (1 μmol/L), and rotenone/antimycin A (0.5 μmol/L). Basal respiration values were extrapolated from the kinetic graph. OCR measurements were normalized to cell counts determined by DAPI nuclei staining. Data represent the mean ± SD of one to two independent experiments, each with at least six technical replicates. (E) Representative Western blot showing the expression levels of the AIF, CHCHD4 and its substrate (MICU1) in response to either 0.04% DMSO as a negative control or 20 μmol/L M30-E05 treatment over time (48 and 72 h). Vinculin was used as a loading control.

PDX models reflect the complexity of heterogeneity of human cancers more accurately than cell lines. After a first passage through immunodeficient mice, PDX can be either transplanted again into mice or subjected to secondary culture *in vitro*. Usually, even osteosarcoma PDX that grow *ex vivo* cannot be cultured >3 passages without losing their fitness^21^. PDX from 3 patients that were xenografted in NSG mice either subcutaneously (SC) or orthotopically/paratibially (PT) all expressed AIF and CHCHD4 at the mRNA and protein levels (Fig. 5A and B) with a clear mitochondrial localization of AIF and CHCHD4 (Supporting Information Fig. S6A‒S6C). Transmission electronic microscopy observation revealed some variability in the morphology (round *vs* elongated) and ultrastructure of the mitochondria that is in line with the variability in basal OCR of the PDX and their grafting location, suggesting an effect of the tumor microenvironment (Fig. 5D, Supporting Information Fig. S5C). Secondary subcultures of these PDX models showed reduced viability upon M30-E05 treatment, with IC_50_ values ranging from 17 to 40 μmol/L (Fig. 5C), which is comparable to IC_50_ obtained with etoposide and CIS, two standard chemotherapeutic agents used in clinics (Fig. S5A and S5B). M30-E05 treatment also decreased basal OCR in all PDX models (Fig. 5D), accompanied by a net decrease in AIF, MICU1 and CHCHD4 protein levels in some PDX cultures (Fig. 5E).

### 3.5 Safety and antitumor activity of M30-E05 in subcutaneous osteosarcoma PDX *in vivo*

To evaluate the potential binding of M30-E05 to off-targets, the compound was tested at 10 mmol/L in 1% DMSO in binding and enzyme and uptake assays (Eurofins Discovery, Cerep, Celle levescault, France). Among 18 human targets tested, M30-E05 showed some moderate inhibitory or stimulatory effects on only 4 targets, namely 5-hydroxytryptamine receptor 2B (5-HT2B), dopamine transporter, Nav1.5 human sodium ion channel and phosphodiesterase 4D2 (PDE4D2). M30-E05 did not inhibit the potassium human Ether-à-go-go-Related Gene (hERG) channel, a major effector, whose inhibition can induce cardiotoxicity nor the tyrosine kinase Lck. The Lck is a member of Src kinase family (SKF) and is important for the activation of T-cell receptor (TCR) signaling in both naive T cells and effector T cells involved in anticancer immunosurveillance (data not shown).

**Figure 6:**
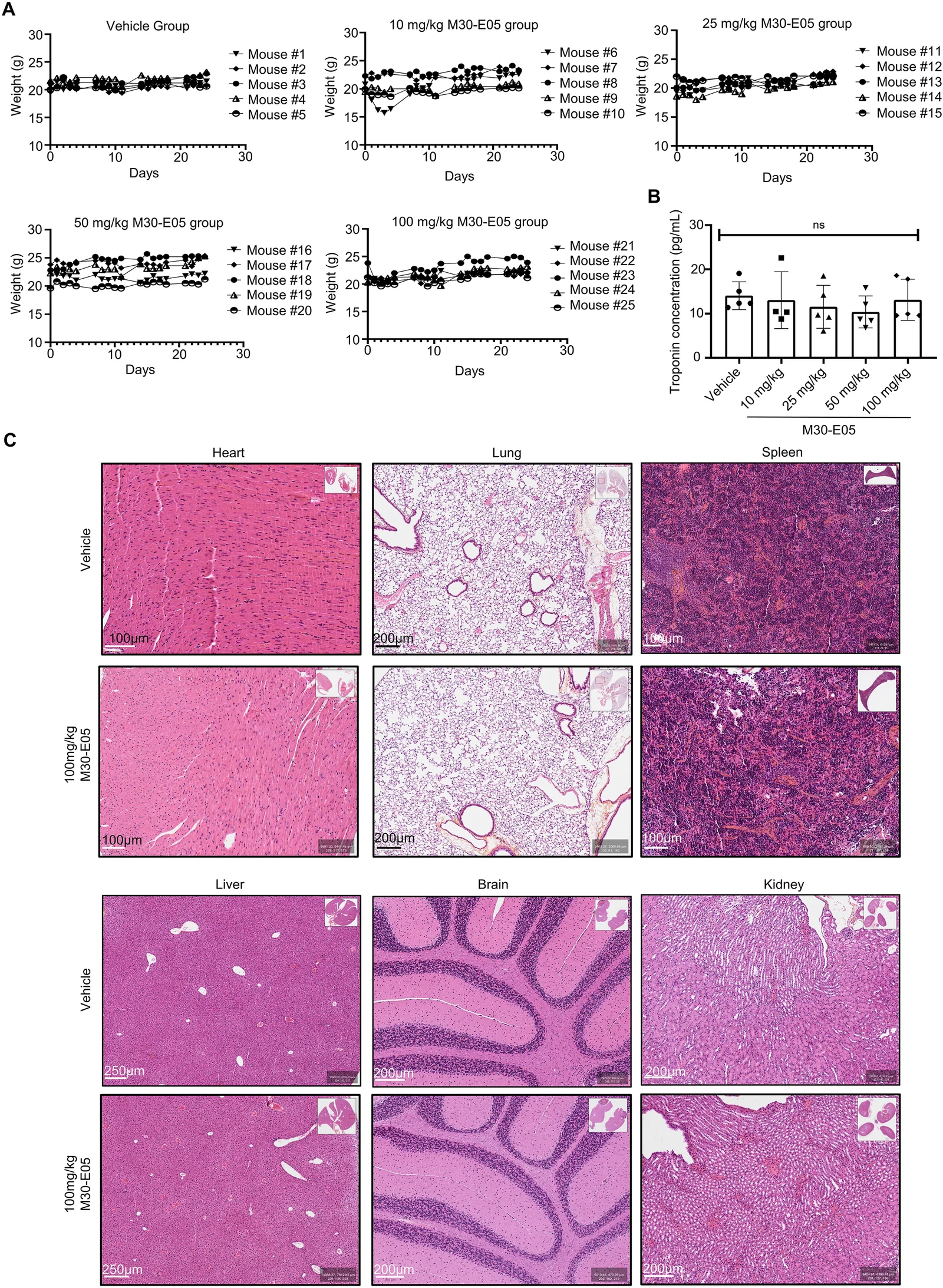
M30-E05 does not display any visible toxicity in non-engrafted NOD.Cg-PrkdcscidIL2rgtm1Wjl/SzJ (NSG) mice. (A) Body weight curves of NSG mice orally administered with 0.5% methylcellulose (Vehicle), 10, 25, 50, or 100 mg/kg M30-E05, five days per week for 4 weeks. Body weight of 5 mice per group was recorded on treatment days. Each curve represents an individual mouse. (B) Serum cardiac troponin levels were quantified using a mouse cardiac troponin-I (TNNI3) ELISA Kit. Data represent the mean ± SD. Statistical analysis was performed using one-way ANOVA with Sidak's multiple comparisons test. Significance levels are indicated as follows: ns, not significant; *P* < 0.0332 (∗); *P* < 0.0021 (∗∗); *P* < 0.0002 (∗∗∗); *P* < 0.0001 (∗∗∗∗). (C) Hematoxylin/eosin (H&E) staining of heart, lung, spleen, liver, brain, and kidney tissues from NSG mice orally administered 0.5% methylcellulose (Vehicle) or 100 mg/kg M30-E05, five days per week for 4 weeks.

Then, as a preclinical safety assessment, a dose-escalation study of M30-E05 was performed in immunodeficient NSG mice. M30-E05 was administered orally for 5 days per week over 4 weeks at doses ranging from 10 up to 100 mg/kg, using 0.5% methylcellulose as a control (vehicle) (Fig. 6A). Throughout this period, the behavior, physical appearance, body weight (Fig. 6A) and circulating cardiotoxic marker‒TNNI3 levels were not affected (Fig. 6B). Moreover, Hematoxylin/Eosin staining analysis of major organs (heart, lung, liver, kidney, spleen, brain) revealed no morphological abnormalities (Fig. 6C).

**Figure 7:**
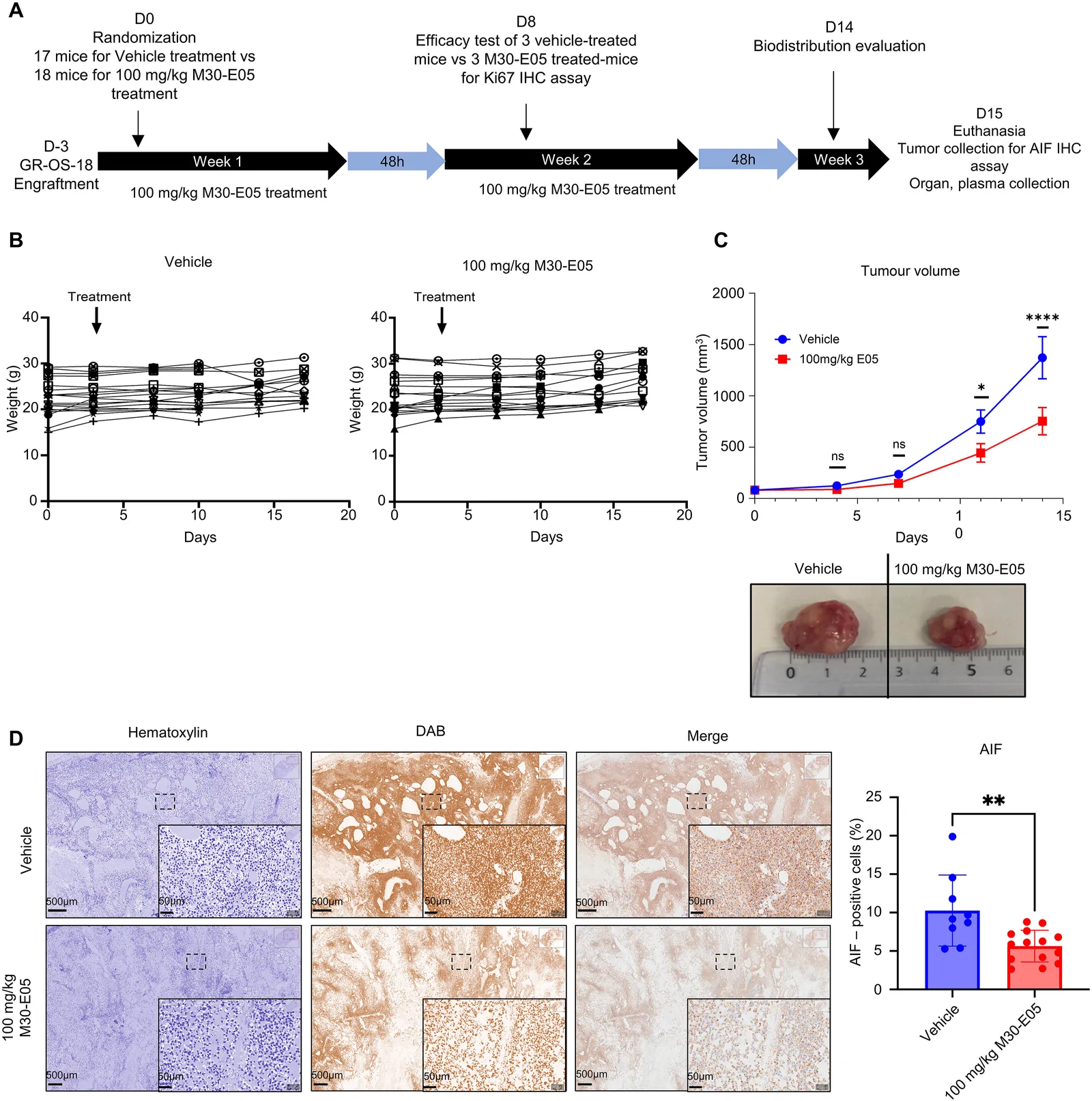
M30-E05 inhibits tumor growth in GR-OS-18 engrafted NSG mice. (A) Experimental design. Mice were engrafted subcutaneously with GR-OS-18 PDX cells. Three days later, mice were orally administrated with 0.5% methylcellulose (Vehicle) or 100 mg/kg M30-E05 five days per week for 2 weeks. The timing of sample collection for efficacy study, biodistribution analysis and sacrifice is indicated. IHC, immunochemistry. (B) Body weight curves of GR-OS-18 engrafted NSG mice administered 0.5% methylcellulose (Vehicle) or 100 mg/kg M30-E05, five days per week for 2 weeks. Body weight (g) was recorded twice per week. Each curve represents an individual mouse. Vehicle group, *n* = 17 mice and 100 mg/kg M30-E05 treated group, *n* = 18 mice. (C) Tumor volume curves of engrafted mice treated with 0.5% methylcellulose (Vehicle) or 100 mg/kg M30-E05. Vehicle group, *n* = 17 mice and 100 mg/kg M30-E05 treated group, *n* = 18 mice. Representative dissected tumors at the end of the experiment derived from vehicle and 100 mg/kg M30-E05-treated mice were shown. Data were shown as mean ± SEM and statistical significance was determined by Two-way ANOVA (mixed model). Significance levels are indicated as follows: ns, not significant; *P* < 0.0332 (∗); *P* < 0.0021 (∗∗); *P* < 0.0002 (∗∗∗); *P* < 0.0001 (∗∗∗∗). (D) IHC analysis of tumor sections for AIF expression from GR-OS-18 engrafted NSG mice administered 0.5% methylcellulose (Vehicle) or 100 mg/kg M30-E05, five days per week for 2 weeks. Vehicle group, *n* = 9 mice and 100 mg/kg M30-E05 treated group, *n* = 14 mice. Data were shown as mean ± SD, and statistical significance was determined by the Mann-Whitney test. Significance levels are indicated as follows: ns, not significant; *P* < 0.0332 (∗); *P* < 0.0021 (∗∗); *P* < 0.0002 (∗∗∗); *P* < 0.0001 (∗∗∗∗).

As M30-E05 demonstrated no toxicity, we next evaluated the anti-tumor efficacy of this compound in the PDX GR-OS-18, which expressed the highest levels of AIF and CHCHD4 among all subcutaneously grown PDXs (Fig. 5B). GR-OS-18 fragments successfully developed into tumors (volume of 80‒150 mm^3^) in NSG mice 3 days after subcutaneous engraftment. Mice were randomly assigned to M30-E05 treatment at 100 mg/kg, administered *via* gavage 5 days per week for 14 days (Fig. 7A). Vehicle-only treated mice served as controls. While no effects on body weight were observed (Fig. 7B), M30-E05 induced a significant reduction of tumor growth by 41% after 2 weeks of treatment (Fig. 7C). Immunohistological staining revealed an M30-E05-induced decrease in Ki-67 expression, the mitotic index biomarker (Supporting Information Fig. S7A) and a reduction of AIF protein expression, suggesting an on-target effect of the drug *in vivo* (Fig. 7D).

Mass spectrometric detection of M30-E05 was performed 2 and 4 h following oral administration to mice. The compound was detected only in the bones and not in the spleen, liver, kidney, lung, plasma, heart, or brain (Fig. S7B). These findings align with the known pharmacokinetics of flavonoid, M30-E05 being rapidly metabolized following administration and reaching specific organs such as the bones.

## 4 Discussion

Metabolic reprogramming is a hallmark of many cancers; thus, developing compounds targeting this vital process might render a promising approach for the treatment of resistant cancer patients. With the aim of discovering small molecules capable of modulating the metabolic flexibility of malignant cells, we have identified a novel chemically modified flavonoid named M30-E05 among a library of 131 flavonoids from the French national library and the BIOCIS library. For the first time, this compound kills cells through the disruption of the mitochondrial AIF/CHCHD4 complex, as shown by co-immunoprecipitation and apoptosis induction *in vitro*. Moreover, combining M30-E05 with DOXO re-sensitized HOS-R/DOXO cells, with a high ZIP synergy score (50.488). Synergism was also observed with MTX in HOS-R/DOXO (17.697) and DOXO in HOS-R/MTX (11.903), while only additive effects were seen in parental HOS cells with CIS, DOXO, and MTX. These results highlight M30-E05's potential to enhance chemotherapy efficacy in resistant osteosarcoma. Remarkably, M30-E05 is orally bioavailable and mediates significant tumor growth reduction in mice bearing PDX from a refractory osteosarcoma patient.

M30-E05 is a flavonoid, a class of natural compounds well-known for their potent anti-inflammatory and neuroprotective properties that also bear potential as anticancer agents. As recent examples, the flavonoids *Cirsiliol*^37^ and *Baicalin*^38^, ^39^, ^40^ have been shown to induce apoptosis in osteosarcoma cells, as well as other cancer types and promote osteogenic differentiation *in vitro*, respectively. Using similar or lower doses than these compounds, we show that M30-E05 can be orally administered, followed by its redistribution to internal organs such as bones, without accumulating in the heart and the brain. Therefore, the risk of cerebral and cardiac toxicity is minimized, which gives the compound a significant advantage compared to drugs that inhibit oxidative phosphorylation. For instance, a selective complex I inhibitor has recently failed in two Phase I trials for advanced solid tumors and acute myeloid leukemia^41^.

*In silico*, we found that M30-E05 preferentially binds to the NAD-binding pocket of the AIF monomer, suggesting that the molecule may competitively inhibit NAD binding, thereby preventing AIF dimerization and CHCHD4 interaction. Mutation of key residues in the binding site of AIF may confirm their contribution to the M30-E05/AIF interaction. To guide the choice of which amino acid residues to mutate, we used the previous MD simulation of the AIF-M30-E05 complex and performed MM/PBSA calculations to identify the protein residues that make the most important interactions with the ligand (Fig. S1B). Among the dozen identified amino acids, PHE 310, LEU 311, and PHE 482 have been suggested for priority mutation to experimentally confirm the models (Fig. S1C). Consistent with the previous study, we also considered the G308E mutant that has been shown to prevent CHCHD4 binding^19^. Then, it will be possible to investigate how the mutations impact the binding to the AIF/CHCHD4 complex by isothermal titration calorimetry and dynamic scanning fluorescence techniques.

Therefore, our compound exhibits a distinct mechanism compared to the recently identified 4-aminoquinolines, which stabilize AIF dimer and stimulate its interaction with CHCHD4^42^. Among the hits identified, M30-E05, which exhibited the most promising properties *in vitro* and *in cellulo*, was selected over 15 hits for further preclinical evaluation. Our study demonstrates that the cellular and molecular mechanisms of action of M30-E05 involve several key steps. First, M30-E05 disrupts the AIF/CHCHD4 complex, leading to an early disruption of AIF/CHCHD4 interaction, followed by the reduction in the expression levels of both AIF and CHCHD4, as well as that of their substrates COX17 and MICU1, the mitochondrial import of which depends on AIF/CHCHD4. Interestingly, similar to other substrates, CHCHD4 is itself a substrate of the disulfide relay system, its reduction upon M30-E05 treatment is consistent with the consequence of AIF/CHCHD4 disruption and degradation *via* the quality control pathway, as previously described, mediated by YME1L1 protease^19^. Moreover, unchanged mRNA levels of *AIFM1* suggest a possible translational or post-translational event that occurred upon the treatment of M30-E05 to decrease mitochondrial AIF level (Fig. S2F). Thus, the degradative pathway of AIF remains undefined. Using a proteasome inhibitor (MG-132) did not restore the M30-E05-induced reduction of AIF protein expression, suggesting further investigation to fully evidence the degradative pathway of AIF, involved in the M30-E05 mechanism of action (Fig. S2G).

Consequently, these changes point to a disruption of mitochondrial protein import that then directly impacts the biogenesis of respiratory chain complexes and suggests the induction of a mitochondrial stress response. Accordingly, cells treated with M30-E05 exhibited fragmentation of their mitochondrial network, mitochondria relocalization close to the nucleus, and organelle dysfunction. This was evidenced by a significant reduction in mitochondrial oxygen consumption at 6 h after treatment, accompanied by an increase in glycolysis and a strong shift in energy substrate utilization to lipids and amino acids without any TCA cycle alteration. Given these major changes, it is not surprising that M30-E05 compromised cell viability, thus inducing caspase activation and apoptosis.

Leveraging access to RNA-seq data from patients at different stages of tumor development (diagnosis, therapy resistance, and relapse) as well as PDX models and both parental and resistant cell lines, we focused on osteosarcoma to preclinically evaluate the therapeutic potential of our drug candidate, M30-E05. Among pediatric cancers, osteosarcoma, which predominantly affects children, adolescent and young adults^43^, is characterized by high malignancy and a strong propensity for early metastasis, primarily to the lungs^44^^,^^45^. Current treatment options are limited to chemotherapy and surgical resection^46^. However, despite significant progress in pediatric oncology and advancements in precision medicine, no improvement in the survival rates of patients with osteosarcoma has been achieved over the past four decades. Consequently, a substantial proportion of patients with osteosarcoma remain uncured due to chemotherapy resistance or relapse^47^. Importantly, almost no mutations of *AIFM1* were found in any osteosarcoma patients, suggesting that AIF would not exhibit changes in amino acid sequence that could modify the accessibility of the protein to a small molecule and the bioactivity of the compound.

Interestingly, M30-E05 is not a drug efflux pump substrate, and its mechanism of action can overcome drug resistance. RNA-seq analysis of PDX models and osteosarcoma cell lines revealed that mitochondrial function and energetic metabolism are reprogrammed and might be linked to the degree of chemoresistance, metastasis, and tumor aggressiveness^48^^,^^49^. Accordingly, the morphology of the mitochondrial network, mitochondrial respiration, and expression of AIF, CHCHD4, and some of their substrates also changed in these models. Importantly, M30-E05 demonstrated the ability to kill a variety of osteosarcoma cancer cell lines and PDX models, irrespective of their multi-chemoresistant status, with IC_50_ values ∼20 μmol/L for most PDX models. At comparable doses, M30-E05 also exhibited cytotoxic activity against other cancer cell lines, including ovarian, lung, and leukemia, while showing limited toxicity on HEK293 cells and primary human PBMC. This suggests that M30-E05 holds potential for broader application in drug-resistant patients and for some pediatric as well as adult cancers, given their metabolism is dependent of AIF and CHDH4 expression.

*In vivo* effects of M30-E05 were evaluated in immunodeficient mice. The compound was formulated in 0.5% methylcellulose and administered *via* oral gavage five days per week for 4 weeks. Consistent with its chemical properties, M30-E05 was rapidly metabolized, well-tolerated, and found to be non-toxic to main organs at doses up to 100 mg/kg. This would not be due to a failure of M30-E05 to bind murine AIF, since the docking experiments confirmed that the compound could bind similarly to murine and human AIF. At 100 mg/kg, M30-E05 induced a significant tumor growth inhibition of 41%. Interestingly, tumor growth inhibition was accompanied by a decrease in AIF expression, as demonstrated by IHC analysis. If confirmed, this could position AIF as a potential biomarker of treatment responses. Of note, M30-E05 effects varied with the grafting site in mice, suggesting a potential role of the tumoral microenvironment that remains to be investigated.

The efficacy range of M30-E05 is a critical parameter for its drug development. The compound showed cytotoxic activity in the range of 2.5 to 12 μmol/L across osteosarcoma and adult cancer cell lines, which, while promising, may be considered relatively high from a clinical perspective. Among the tested cell lines, ovarian cancer and leukemia cells demonstrated the greatest sensitivity, consistent with their metabolic reprogramming profiles^50^^,^^51^. Furthermore, analysis of patient survival by *AIFM1* and *CHCHD4* mRNA expression levels on R2 Genomics Analysis and Visualization Platform revealed a correlation between high expression and poor patient prognosis in neuroblastoma, prostate adenocarcinoma, lung adenocarcinoma, chromophobe renal cell carcinoma, and lymphoma, highlighting the potential of M30-E05 as a novel pharmacological agent for the treatment of these cancers.

To improve M30-E05 efficacy, several strategies could be pursued: (i) developing improved formulations (*e.g.*, nanoparticles or antibody‒drug conjugates), (ii) designing more potent analogs, (iii) identifying more sensitive cancer types or subtypes for targeted therapy development, or (iv) combining with other treatments such as radiotherapy and/or immunotherapy.

In conclusion, M30-E05, a novel chemically modified flavonoid, acts through an original biological mechanism targeting cancer-specific metabolic reprogramming *via* AIF/CHCHD4 complex disruption. This compound might become a valuable therapeutic option for refractory and relapsed cancer patients, either as monotherapy or in combination treatments.

## Author contributions

Hong Toan Lai: Conceptualization, Methodology, Investigation, Data curation, Formal analysis, Validation, Visualization, Writing - original draft, Project administration. Daniela Dias-Pedroso: Conceptualization, Methodology, Investigation, Validation, Visualization, Writing - original draft. Maria Eugénia Marques Da Costa: Conceptualization, Methodology, Investigation, Writing - review and editing. Romain Fernandes: Investigation, Writing - review and editing. Tran Ngoc Anh Nguyen: Conceptualization, Methodology, Investigation, Data curation, Validation, Visualization, Writing - original draft. Olivia Bawa: Resources, Formal analysis, Writing - review and editing. Pierre Khneisser: Resources, Formal analysis, Writing - review and editing. João Gabriel Silva: Visualization, Writing - review and editing. Yassine Khaliji: Investigation, Writing - review and editing. Michelangelo Campanella: Resources, Writing - review and editing. John Griffith Jones: Resources, Visualization, Writing - review and editing. Svetlana Dokudovskaya: Writing - review and editing. Guido Kroemer: Writing - review and editing. Antonin Marchais: Methodology, Resources, Writing - review and editing. Nathalie Gaspar: Methodology, Resources, Writing - review and editing. Birgit Geoerger: Conceptualization, Methodology, Funding acquisition, Resources, Writing - review and editing. Oumar Diane: Methodology, Investigation, Visualization, Writing - review and editing. Liuba Mazzanti: Methodology, Investigation, Visualization, Writing - review and editing. Tâp Ha Duong: Conceptualization, Methodology, Investigation, Data curation, Software, Formal analysis, Visualization, Writing - review and editing. Guy Lewin: Methodology, Resources, Writing - review and editing. Laurent Ferrié: Methodology, Resources, Writing - review and editing. Bruno Figadère: Conceptualization, Methodology, Resources, Writing - review and editing. Catherine Brenner: Conceptualization, Funding acquisition, Writing - review and editing, Supervision, Project administration.

## Conflicts of interest

GK has been holding research contracts with Daiichi Sankyo, Eleor, Kaleido, Lytix Pharma, PharmaMar, Osasuna Therapeutics, Samsara Therapeutics, Sanofi, Sutro, Tollys, and Vascage. GK is on the Board of Directors of the Bristol Myers Squibb Foundation France. GK is a scientific co-founder of everImmune, Osasuna Therapeutics, Samsara Therapeutics and Therafast Bio. GK is in the scientific advisory boards of Centenara Labs (formerly Rejuveron Life Sciences), Hevolution, and Institut Servier. GK is the inventor of patents covering therapeutic targeting of aging, cancer, cystic fibrosis and metabolic disorders. Among these, patents were licensed to Bayer (WO2014020041-A1, WO2014020043-A1), Bristoll-Myers Squibb (WO2008057863-A1), Osasuna Therapeutics (WO2019057742A1), PharmaMar (WO2022049270A1 and WO2022048775-A1), Raptor Pharmaceuticals (EP2664326-A1), Samsara Therapeutics (GB202017553D0), and Therafast Bio (EP3684471A1). GK'wife, Laurence Zitvogel, has held research contracts with Glaxo Smyth Kline, Incyte, Lytix, Kaleido, Innovate Pharma, Daiichi Sankyo, Pilege, Merus, Transgene, 9 m, Tusk and Roche, was on the on the Board of Directors of Transgene, is a cofounder of everImmune, and holds patents covering the treatment of cancer and the therapeutic manipulation of the microbiota. Among these, patents were licensed to everImmune (US20210346438A1, US20200360449A1) and Transgene (US20200376052A1). GK's brother, Romano Kroemer, was an employee of Sanofi and now consults for Boehringer-Ingelheim. The funders had no role in the design of the study; in the writing of the manuscript, or in the decision to publish the results. Nathalie Gaspar has got consultant activity for EISAI and IPSEN, and participate to advisory board for ABBVIE, MERCKS, Y-MABS, Daiichi Sankyo/AstraZeneca.

## Appendix A Supporting information

The following is the Supporting Information to this article:
