# Supplementary material for: Engineered flavonoid disrupts mitochondrial AIF/CHCHD4 complex for targeted cancer therapy
Source: Acta Pharm Sin B. 2026 Jun 15;16(9):6145–67. doi: 10.1016/j.apsb.2026.06.022 (PMC13589956; doi:10.1016/j.apsb.2026.06.022)
Supplement: Supplementary file 1 [file mmc1.pdf]

Supporting Information for

Original article

Engineered flavonoid disrupts mitochondrial AIF/CHCHD4 complex for targeted cancer therapy

Hong Toan Lai[a], Daniela Dias-Pedroso[a], Maria Eugénia Marques Da Costa[b], Romain Fernandes[a], Tran Ngoc Anh Nguyen[a], Olivia Bawa[c], Pierre Khneisser[d, e], João Gabriel Silva[f], Yassine Khaliji[a], Michelangelo Campanella[a], John Griffith Jones[f], Svetlana Dokudovskaya[a], Guido Kroemer[g,h,i], Antonin Marchais[b], Nathalie Gaspar[b,j], Birgit Geoerger[b], Oumar Diane[k], Liuba Mazzanti[k], Tâp Ha Duong[k], Guy Lewin[k], Laurent Ferrié[k], Bruno Figadère[k], Catherine Brenner[a,*]

[a]*CNRS, Institut Gustave Roussy, Aspects métaboliques et systémiques de l'oncogénèse pour de nouvelles approches thérapeutiques, Université Paris-Saclay, Villejuif 94805, France*

[b]*INSERM UMR 1015, Gustave Roussy Cancer Campus, Villejuif 94805, France*

[c]*INSERM US23, CNRS UAR 3655, AMMICa, Experimental and Translational Pathology Platform, Gustave Roussy, Villejuif 94805, France*

[d]*Department of Pathology, Gustave Roussy Cancer Campus, Villejuif 94805, France*

[e]*Université Paris-Saclay, Institut Gustave Roussy, Inserm, Immunologie anti-tumorale et immunothérapie des cancers, Villejuif 94805, France*

[f]*Centre for Innovative Biomedicine and Biotechnology, and III-UC - Institute of Interdisciplinary Research, University of Coimbra, Coimbra 3004-504, Portugal*

[g]*Centre de Recherche des Cordeliers, Equipe labellisée par la Ligue contre le cancer, Université Paris Cité, Sorbonne Université, Inserm U1138, Institut Universitaire de France, Paris 75006, France*

[h] *Metabolomics and Cell Biology Platforms, Institut Gustave Roussy, Villejuif 94805, France*

[i] *Institut du Cancer Paris CARPEM, Department of Biology, Hôpital Européen Georges Pompidou, AP-HP, Paris 75015, France*

[j] *Department of Oncology for child and adolescent, Gustave Roussy, Villejuif 94805, France*

[k]*BioCIS, CNRS, Université Paris-Saclay, Orsay 91400, France*

Received 30 September 2025; received in revised form 28 November 2025; accepted 19 May 2026

*Corresponding author.

E-mail address: catherine.brenner@gustaveroussy.fr (Catherine Brenner).

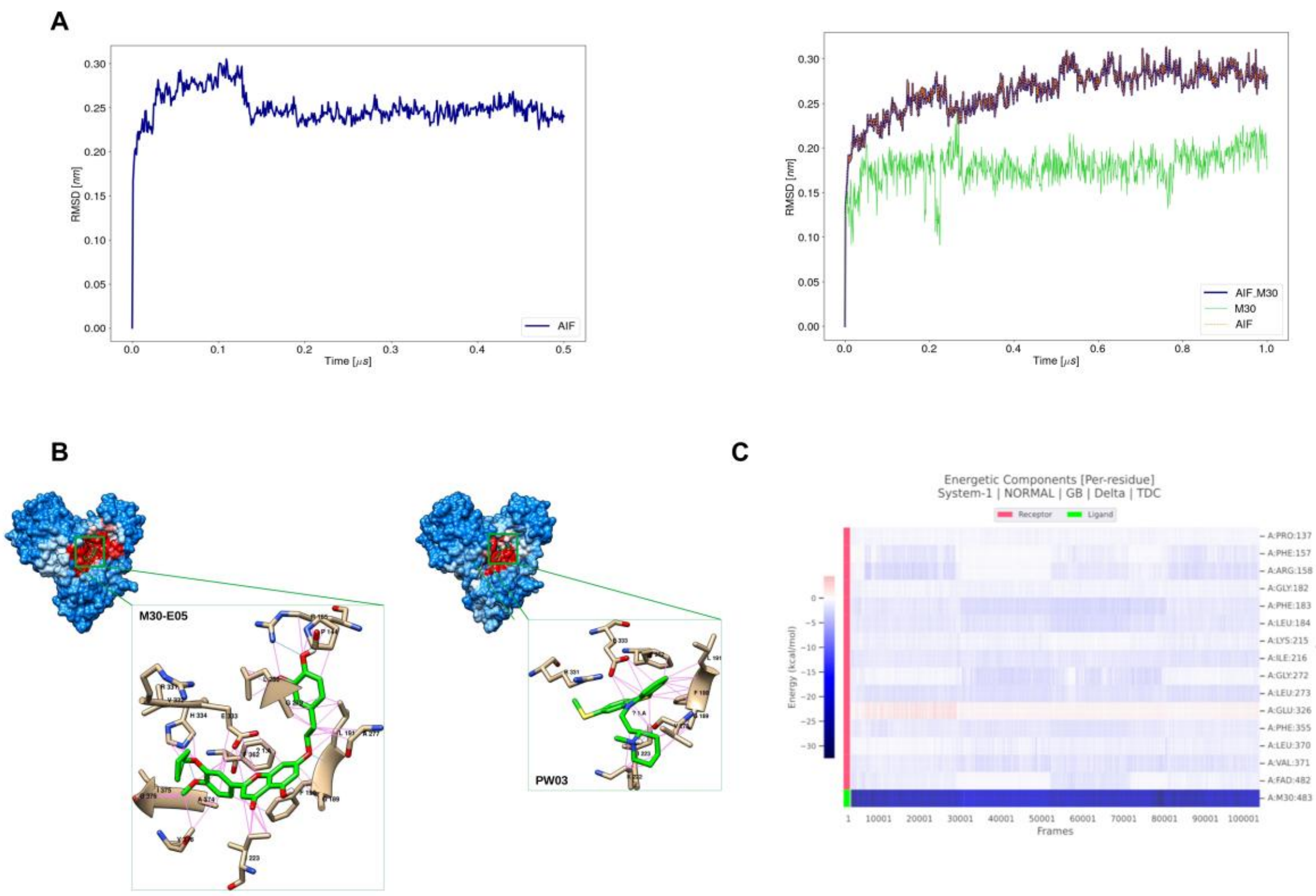

Figure S1. Stability of the unbound AIF and AIF-M30-E05 complex during MD simulations, and AIF residues involved in binding M30-E05. A, Left panel: Time evolution of the root mean square deviation (RMSD) of AIF three-dimensional structure with respect to its initial conformation, along AIF molecular dynamic (MD) simulation trajectory. Right panel: RMSD as a function of time for AIF (blue solid line), M30-E05 (green solid line), and AIF-M30-E05 complex (orange dashed line), along the trajectory of AIF-M30-E05 complex MD simulation. **B,** AIF amino acid residues that interact with M30-E05 (left) or PW03 compound (right) for their respective best docking binding mode to the protein. **C,** Binding free energy decomposition per residue evaluated along the AIF-M30-E05 complex MD simulations. Only AIF residues within a 0.6 nm cutoff distance from the ligand are shown.

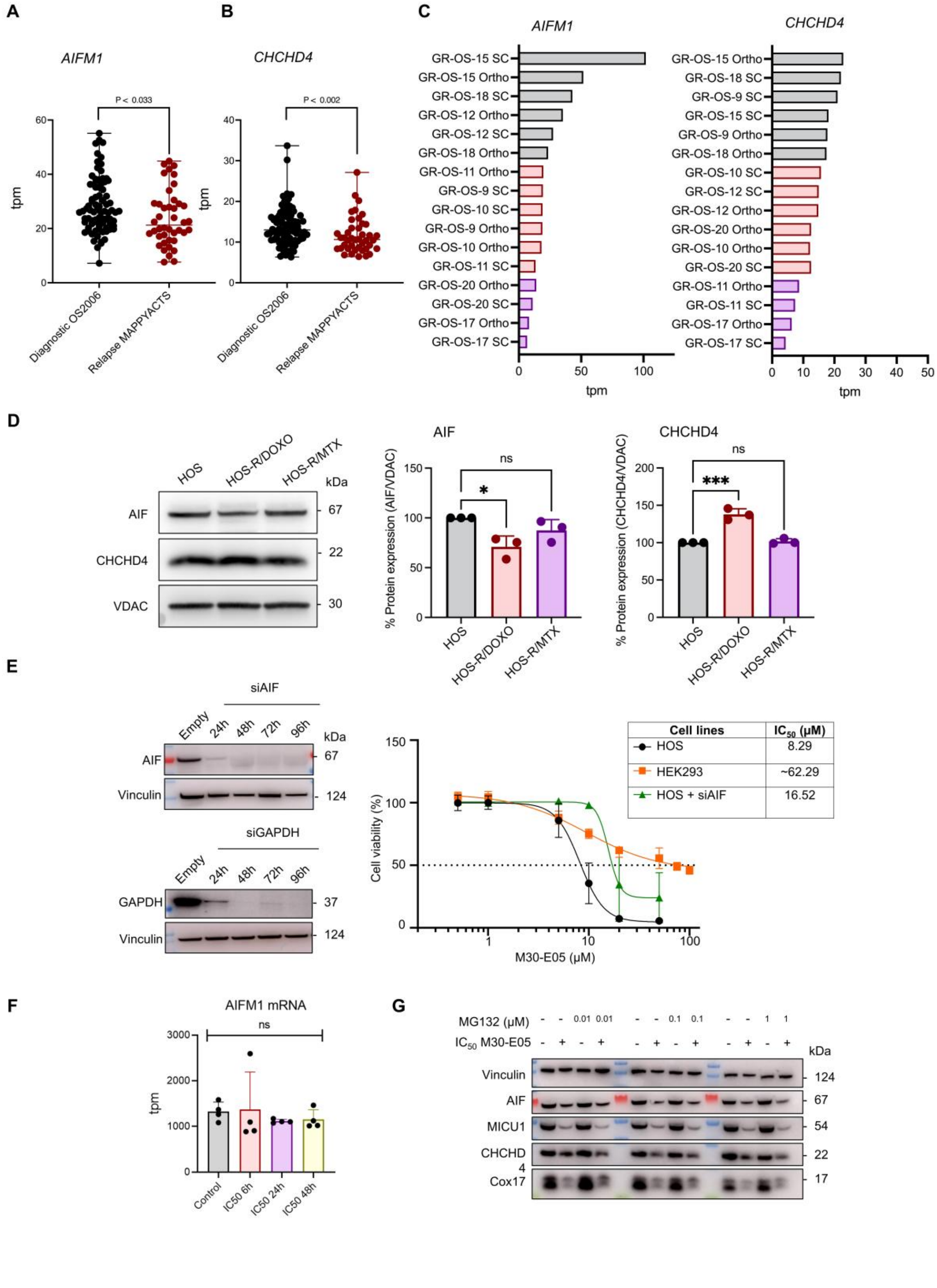

Figure S2. **AIF/CHCHD4 expression across patients, PDXs, and cell lines, M30-E05 responses**

in AIF-silenced HOS and non-cancerous cells and the independence of proteasomal degradation. A, B, *AIFM1* and *CHCHD4* mRNA expression was determined in patients at diagnosis (OS2006) vs relapse (MAPPYACTS) and **C,** in 8 PDX xenografted subcutaneously (SC) and paratibially (PT) in tpm (transcript per million). Data were shown as mean ± SD and statistical significance was determined by Mann-Whitney test for each time point. Significance levels are indicated as follows: ns, not significant; *p < 0.0332; **p < 0.0021; ***p < 0.0002; **p < 0.0001. **D,** Expression of AIF and CHCHD4 in HOS cells, HOS-R/DOXO and HO-R/MTX cells were analyzed by Western-blot with VDAC as loading control and protein expression was quantified in ratio to VDAC. A representative western-blot is presented while the experiment was repeated 3 times. Data were shown as mean ± SD. VDAC was used as a loading control. Statistical analysis was performed using one-way ANOVA with Sidak's multiple comparisons test. Significance levels are indicated as follows: ns, not significant; p < 0.0332 (*); p < 0.0021 (**); p < 0.0002 (***); p < 0.0001 (****). **E**, Representative Western blot analysis of AIF-siRNA (siAIF) treatment over time (top panel), with siGAPDH as a positive control (bottom panel). Based on this, AIF was silenced 48 h before exposure to increasing concentrations of M30-E05. Vinculin was used as a loading control. Cell viability is measured using an LDH assay. Dose-response curves show cell viability at increasing M30-E05 concentrations after 72 h of treatment. HEK293 (immortalized human embryonic kidney cells) were used as non-cancerous cell line. Individual experiments are shown (n > 2). Data were shown as mean ± SD. IC_{50}, inhibitory concentration yielding to 50% cell viability. 0.002% DMSO was used as the solvent of M30-E05 and as a control for calculation of percentage of viability. **F**, Transcriptomic (RNA-seq) quantification of *AIFM1* mRNA expression in M30-E05-treated HOS cells for 6, 24, or 48 h. Bars show mean ± SD; dots represent biological replicates. One-way ANOVA with Šidák's multiple-comparisons test was performed; ns, not significant (p > 0.05). **G**, Representative immunoblot of HOS cells pretreated for 1h with MG-132 or vehicle (0.00002% DMSO), followed by 24 h exposure to IC_{50} of M30-E05.

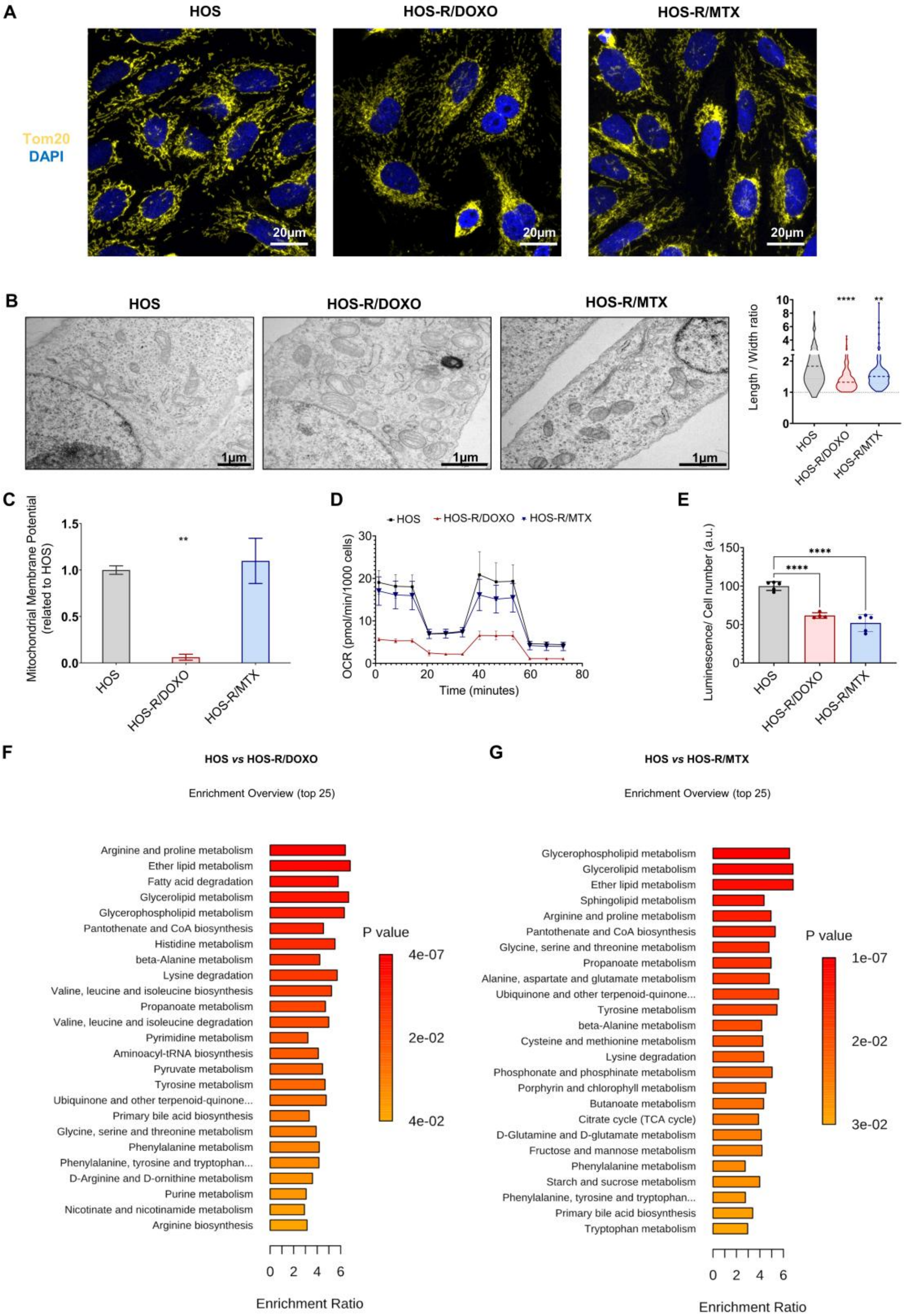

Figure S3. Mitochondrial network metabolic activity is altered by drug-induced resistance in

HOS cells. A, Representative confocal images of HOS, HOS-R/DOXO and HOS-R/MTX's mitochondrial network. Mitochondria are labelled with TOM20 antibody and nuclei were stained with DAPI. Four independent experiments were performed, and Z-stack projections of each condition were evaluated. **B,** Representative transmission electron microscopy images of HOS, HOS-R/DOXO and HOS-R/MTX's cells, showing differences in mitochondria shape and cristae density. Ratios between the length and width of mitochondria of each condition were measured (n>100). Data is represented as violon plots, and the median of each condition is shown by the horizontal dashed line. A ration equal to 1 indicates that mitochondria have a round shape. Statistical significance was assessed using ANOVA with Dunnett's multiple comparison test correction. Significance levels are indicated as follows: $p < 0.01$ (**), and $p < 0.0001$ (****) *vs* HOS. **C,** Mitochondrial membrane potential assessed by flow cytometry using 100nM TMRM. fluorescent probe labelling for 20 min. At least 4 independent experiments were performed ($n \geq 4$). Data represent the mean±SD. Statistical significance was assessed using ANOVA with Dunnett's multiple comparison test correction. Significance levels are indicated as follows: < 0.01 (**) *vs* HOS. D**,** Seahorse XFe96 Mito Stress Test graph displaying the OCR values (pmol/min/1.000 cells) over time in HOS, HOS-R/DOXO and HOS-R/MTX cells. The test was conducted using the following compounds: Oligomycin (2.5 µM), FCCP (1 µM), and Rotenone/Antimycin A (0.5 µM). Three independent experiments were performed. **E,** ATP levels were semi-quantified over time in HOS, HOS-R/DOXO and HOS-R/MTX cells, using the ATPlite assay ($n \geq 4$). Data represent the mean±SD. Statistical analysis was performed using ANOVA with Dunnett's multiple comparison test correction. Significance levels are indicated as follows: $p < 0.0001$ (****) vs HOS. **F, G,** Enrichment analysis of metabolites that differ significantly between HOS and HOS-R/DOXO **(F)** and between HOS and HOS-R/MTX **(G)** cell lines. The enrichment ratio represents the observed number of metabolites in a specific metabolic pathway divided by the expected number. Metabolic pathways are ranked by p-value, with the most significant pathways at the top.

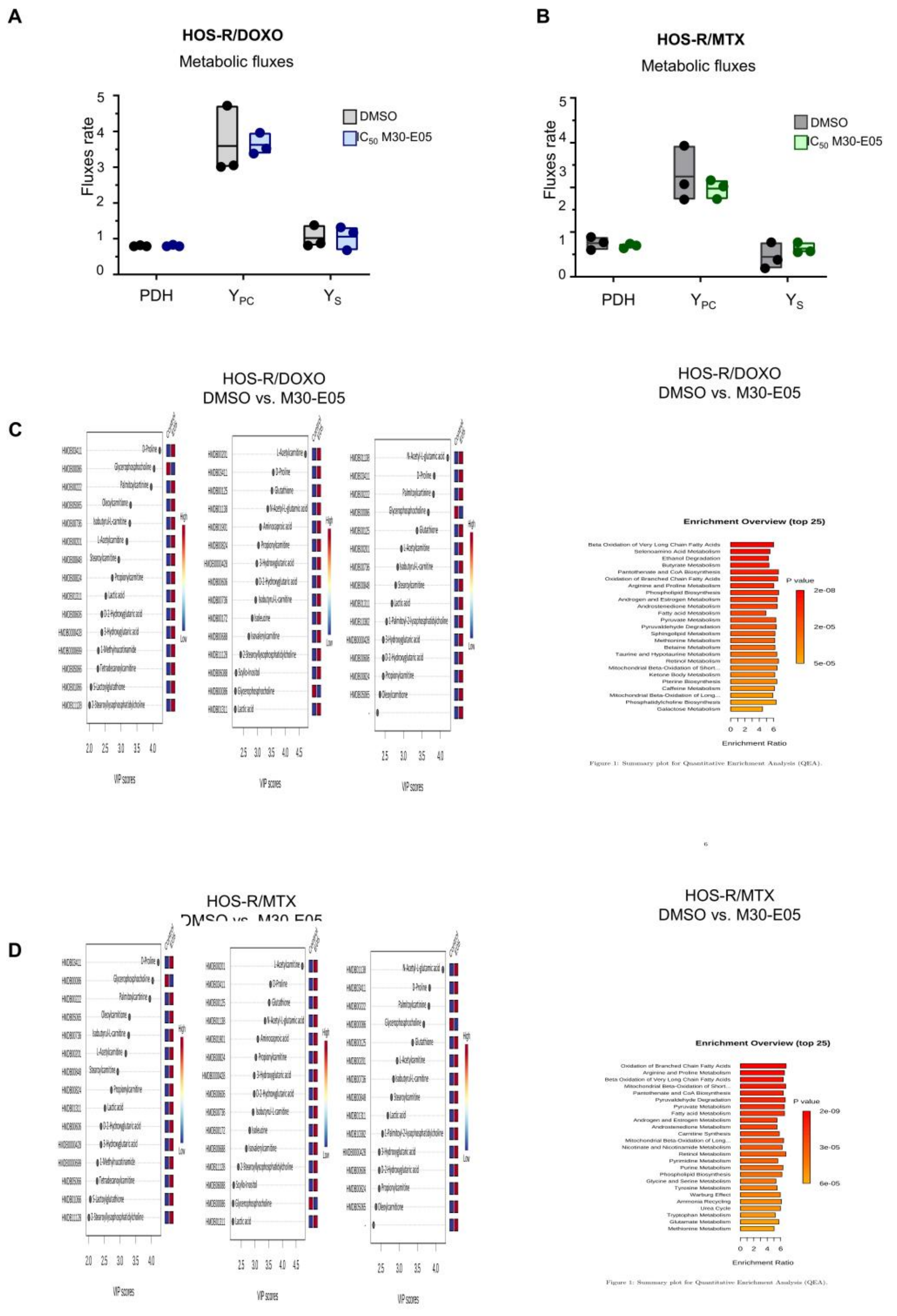

Figure S4. tcaCALC metabolic modelling of the fraction of acetyl-CoA derived from pyruvate *via* PDH, anaplerotic pyruvate carboxylase flux relative to that of citrate synthase (Y_{PC}) and anaplerotic fluxes from unlabeled non-pyruvate substrates relative to that of citrate synthase (Y_S). **A, B,** Fraction of acetyl-CoA for **(A)** HOS-R/DOXO and **(B)** HOS-R/MTX cells, with either vehicle (DMSO) (n=3) or M30-E05 administered at the IC50 concentration (n=3). Statistical analysis was performed using multiple unpaired t-tests with Welch correction ($\alpha = 0.95$), with FDR correction

for multiple comparisons. **C, D,** Left panel: VIP score plot depicting metabolites that significantly differentiate between 0.002% DMSO as a negative control and IC_{50} (8.3 μM) M30-E05-treated osteosarcoma cell lines following 72 h of treatment. Two vertical box plots on the right illustrate metabolic variations in 0.002% DMSO *versus* M30-E05-treated **(C)** HOS-R/DOXO, **(D)** HOS-R/MTX, respectively. Right panel: Enrichment analysis of metabolites that differ significantly between 0.002% DMSO as a negative control and IC_{50} (8.3 μM) M30-E05-treated cells in **(C)** HOS-R/DOXO, and **(D)** HOS-R/MTX cell lines following 72 h of treatment. The enrichment ratio represents the observed number of metabolites in a specific metabolic pathway divided by the expected number. Metabolic pathways are ranked by p-value, with the most significant pathways at the top.

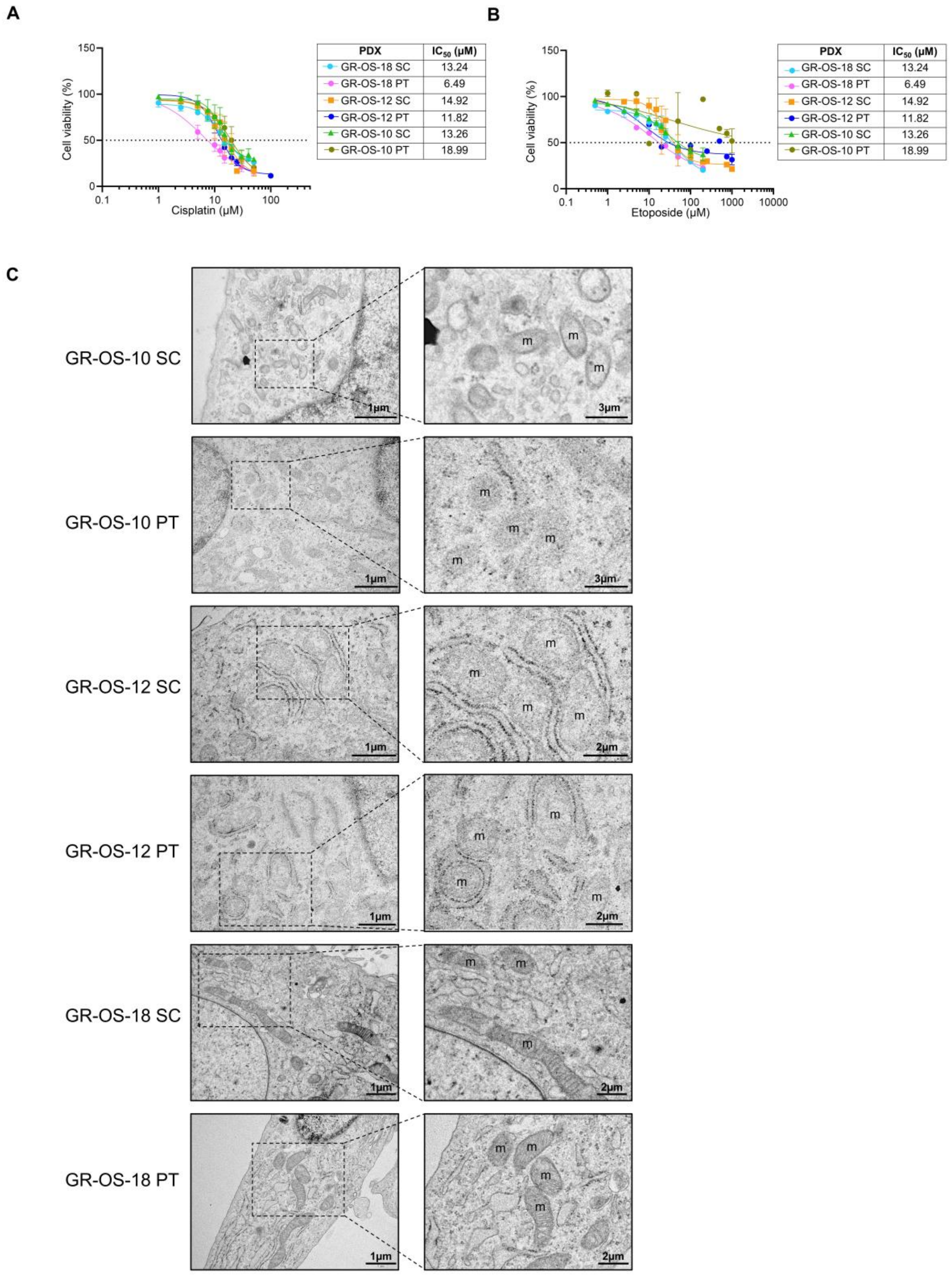

Figure S5. Reponses to standard treatments of osteosarcoma and mitochondrial ultrastructure in secondary cultured PDX samples. A, B, Cell viability of 6 different secondary cultured in vitro PDX samples treated with **(A)** Cisplatin and **(B)** Etoposide – osteosarcoma standard clinical treatments, measured using an LDH assay. Dose-response curves represent cell viability at increasing

concentrations of M30-E05 after 72 h of treatment. Individual experiments are shown (n > 2). Data were shown as mean ± SD. IC_{50}, inhibitory concentration yielding to 50% cell viability. 0.002% PBS was used as a control for calculation of percentage of viability. **(C)** Representative transmission electron microscopy images of 6 different secondary cultured *in vitro* PDX samples in basal condition, showing different unique mitochondrial ultrastructure. m: mitochondrion.

A

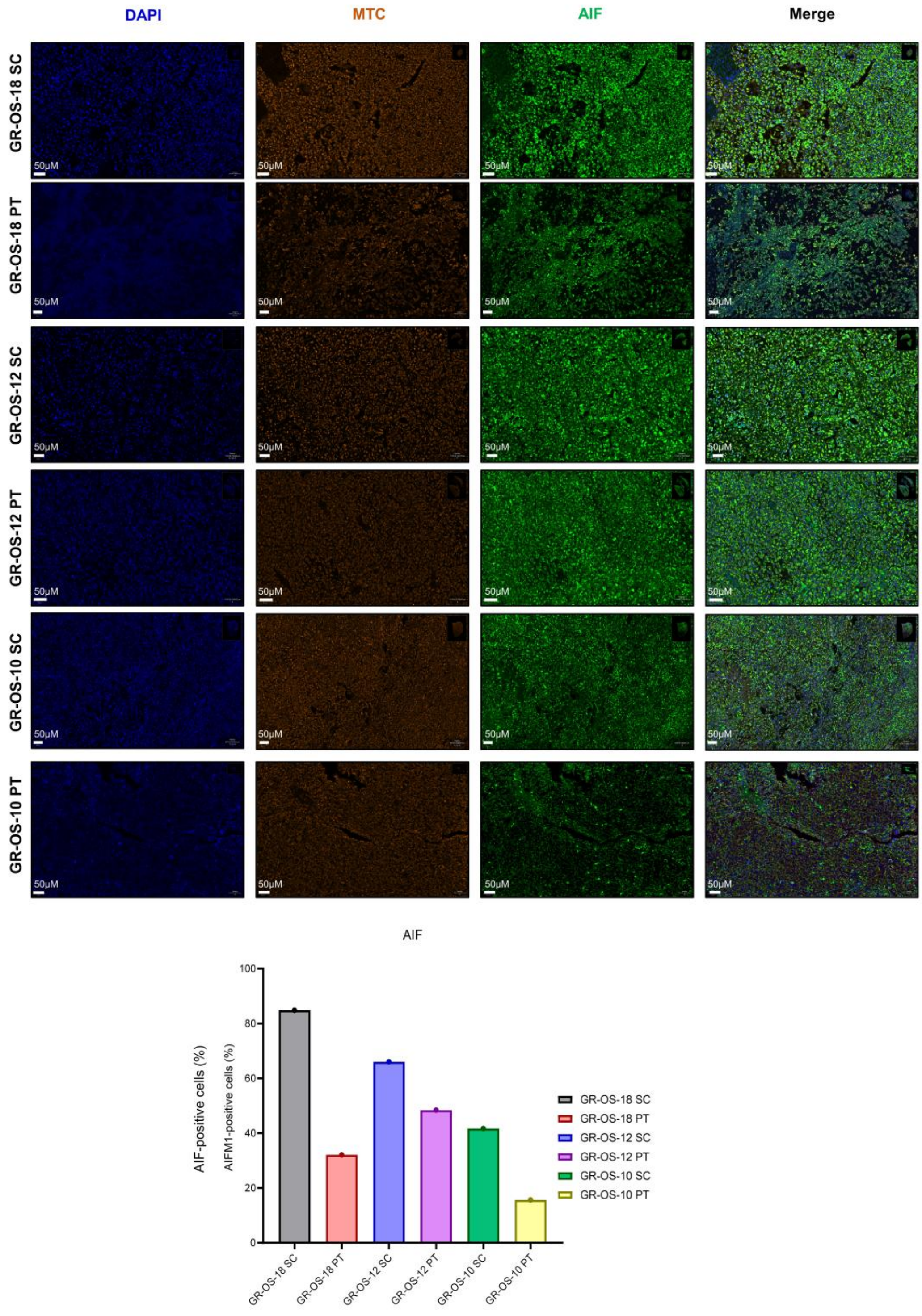

Figure S6. Immunofluorescence analysis of AIF, CHCHD4 and MICU1 expression in PDX

tumor sections. Immunofluorescence analysis of tumor sections for **a,** AIF; **b,** CHCHD4; **c,** MICU1 expression from all 6 PDX models engrafted NSG mice. Blue fluorescence was stained to locate nucleus by Hoechst 33342, orange fluorescence was stained to mitochondria by MTC antibody, green fluorescence was stained to our target (AIF, CHCHD4 or MICU1) by their specific antibody. Digital analysis was performed using QuPath software. Multiplex immunofluorescence analysis was obtained from a single experiment.

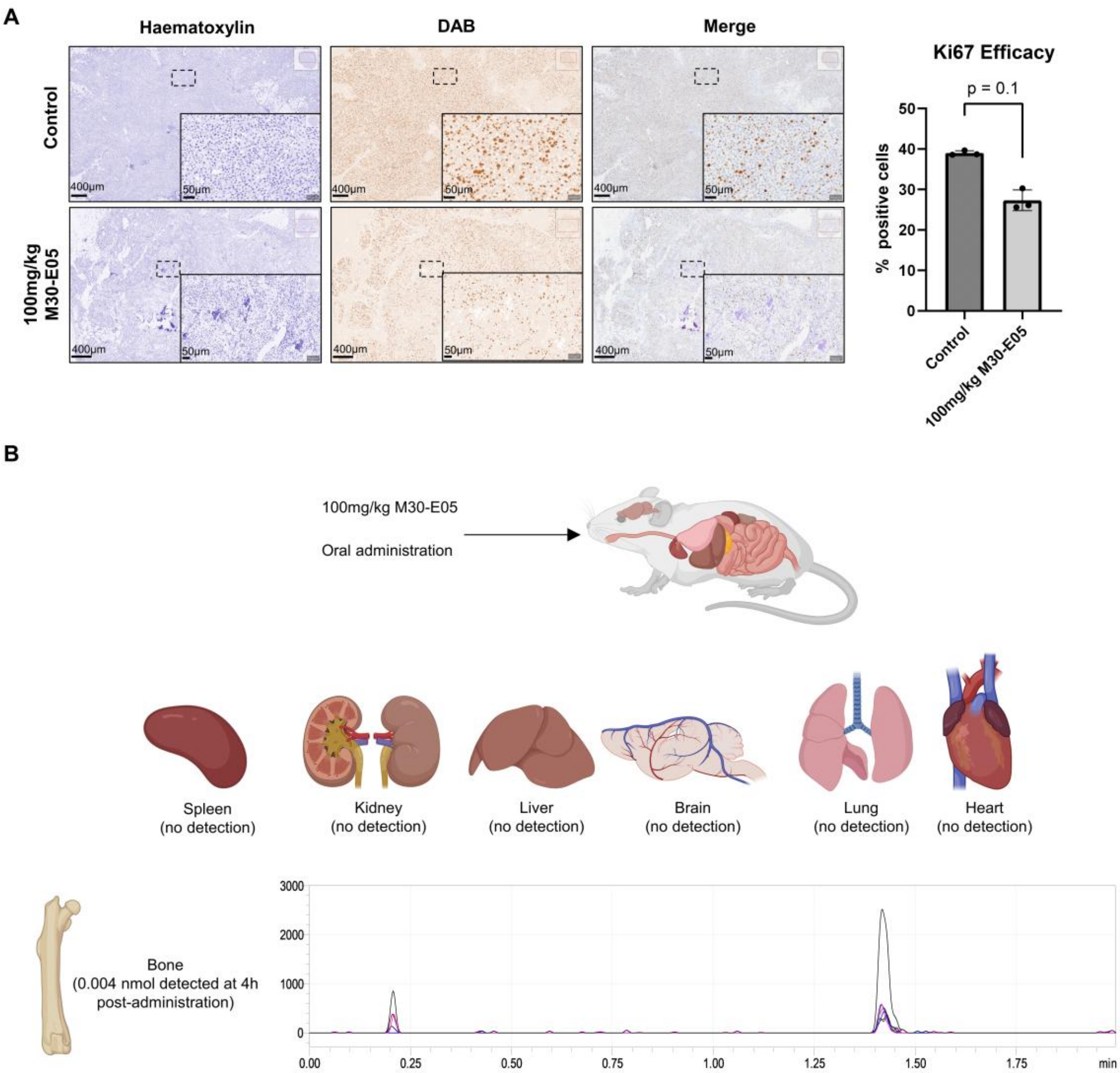

Figure S7. Ki67 expression in GR-OS-18 tumor sections following treatment with M30-E05 and Biodistribution of M30-E05 in several organs. (A) IHC analysis of tumor sections for Ki67 expression from 6 GR-OS-18 engrafted NSG mice administered 0.5% methylcellulose (Vehicle) (n =3) or 100 mg/kg M30-E05 (n = 3), five days per week for 2 weeks. 3 Vehicle-treated mice and 3 100 mg/kg M30-E05 treated-mice were euthanized at day 8 of treatment. Digital analysis was performed using QuPath software. Data were shown as mean ± SD and statistical significance was

determined by Mann-Whitney test. Significance levels are indicated as follows: ns, not significant; $p < 0.0332$ (*); $p < 0.0021$ (**); $p < 0.0002$ (***); $p < 0.0001$ (****). **(B)** Biodistribution of M30-E05 in mouse brain, lung, heart, spleen, kidney, bone and liver after oral administration of 100 mg/kg M30-E05 at 2 h and 4 h post-administration. The organs were sampled for biodistribution analysis with liquid chromatography-tandem mass spectrometry (LC-MS/MS).

Synthesis of M30-E05 : Protocols and Products Characterizations

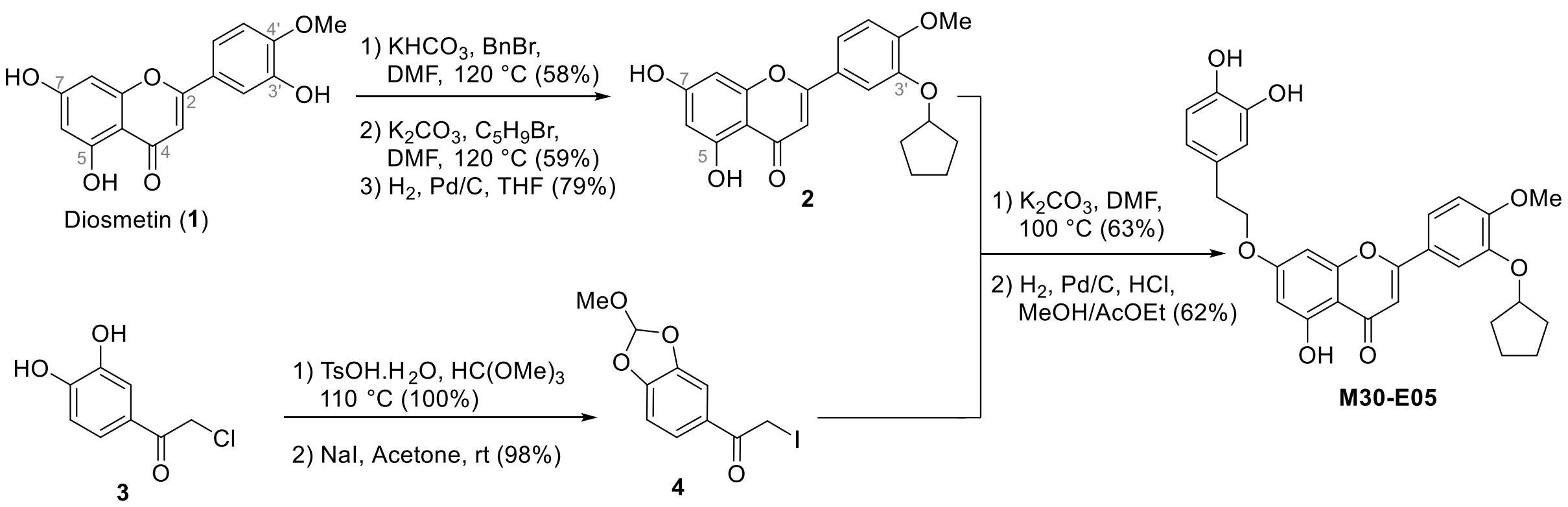

3’-O-cyclopentyl-diosmetin **2**

To a solution of diosmetin (21.0 g, 70 mmol, 1 equiv) in DMF (200 mL) was added $KHCO_3$ (7g, 70 mmol, 1 equiv). The reaction mixture was heated at 120 °C and benzyl bromide (12.5 mL, 105 mmol, 1.5 equiv) was added and the reaction mixture was heated for 150 min at that temperature. TLC monitoring indicated that starting material was remaining, but the reaction was stopped to keep selectivity. After cooling the reaction to rt, 1:1 AcOEt: Toluene mixture (200 mL) was added and the reaction mixture was filtered over celite, washing with the celite pad with 1:1 AcOEt: Toluene mixture. After concentration to dryness on a rotavapor, hot EtOH (200 mL) was added and the reaction mixture was let crystallizing over week-end at rt. The crystals were recovered by suction, washed with EtOH, Et_2O and pentane. After drying, 7-O-benzyl-diosmetin was recovered as yellow crystals (16.0 g, 58%), contaminated with about 8% of starting material.

Mp : 204-206 °C. **^{1}H NMR** (300 MHz, THF-*d*8) δ 12.97 (s, 1H), 8.28 (s, 1H), 7.52 – 7.42 (m, 4H), 7.42 – 7.25 (m, 4H), 7.02 (d, *J* = 8.3 Hz, 1H), 6.66 (s, 1H), 6.40 (d, *J* = 2.0 Hz, 1H), 5.19 (s, 2H), 3.92 (s, 3H). **^{13}C NMR** (75 MHz, THF) δ 183.2, 165.7, 165.1, 163.6, 158.8, 152.0, 148.4, 137.7, 129.4, 129.3, 128.9, 128.5, 125.2, 119.3, 114.1, 112.1, 105.1, 99.5, 93.9, 71.2, 56.4. **IR** (cm^{-1}) : 3346, 1655, 1602, 1495, 1431, 1356, 1250, 1157, 1115, 1022, 829, 763. **HRMS-ESI** (*m /z*) : calcd for $[M+H]^+$ $C_{23}H_{19}O_6$, 390.1182 ; Found : 390.1179.

To a solution of 7-O-benzyl-diosmetin (16.0 g, 41 mmol) in DMF (150 mL) was added K_2CO_3 (7.4 g, 53.3 mmol, 1.3 equiv). The reaction mixture was heated at 120 °C, cyclopentyl bromide (6.2 mL, 61.5 mmol, 1.5 equiv.) was added and the reaction mixture was stirred at that temperature for 5 h, until complete conversion by TLC monitoring. The reaction mixture was cooled down to rt, and was quenched by water (200 mL) and diluted with AcOEt (400 mL). The organic layer was washed 3 times with 1N HCl, and then with brine. The organic layer was dried over $MgSO_4$, filtered,

concentrated and the residue was recrystallized with hot MeOH to give 3'-O-cyclopentyl-7-O-benzyl-diosmetin as yellow/brown crystals (11.0 g, 59%).

Mp : 200-202 °C **^{1}H NMR** (300 MHz, Chloroform-*d*) δ 12.82 (s, 1H), 7.48 (dd, *J* = 8.6, 2.2 Hz, 1H), 7.45 – 7.35 (m, 5H), 7.33 (d, *J* = 2.3 Hz, 1H), 6.95 (d, *J* = 8.6 Hz, 1H), 6.60 – 6.51 (m, 2H), 6.44 (d, *J* = 2.3 Hz, 1H), 5.13 (s, 2H), 4.85 (tt, *J* = 6.3, 3.3 Hz, 1H), 3.92 (s, 3H), 2.07 – 1.77 (m, 6H), 1.74 – 1.55 (m, 2H). **^{13}C NMR** (75 MHz, $CDCl_3$) δ 182.5, 164.6, 164.3, 162.3, 157.7, 153.5, 148.0, 135.9, 128.9, 128.5, 127.6, 123.7, 120.0, 112.4, 111.7, 105.8, 104.6, 98.8, 93.7, 80.9, 70.6, 56.2, 32.9, 24.2. **IR** (cm^{-1}) : 2951, 1655, 11585, 1497, 1354, 1252, 1157, 1020, 831, 766, 704, 627. **HRMS-ESI** (*m /z*) : calcd for $[M+H]^+$ $C_{28}H_{27}O_6$, 459.1808 ; Found : 459.1805.

To a solution of 3'-O-cyclopentyl-7-O-benzyl-diosmetin (11.0 g, 24.0 mmol, 1 equiv) in THF (200 mL) in a Parr reactor was added Pd/C 10% (1.0 g). The reaction mixture was stirred under H_2 atmosphere (10 bars) for 24 h under vigorous stirring at rt. The reaction mixture was then filtered on celite and concentrated. The residue was triturated with hot DCM (50 mL) and was let cooling in the freezer. The solid was then recovered by suction, washed with cold DCM, then with Et_2O. The solid was then dried under vacuum and pure 3'-O-cyclopentyl-diosmetin (**2**) was recovered as a light brown powder (7.0 g, 79 %).

Mp : 207-209 °C. **^{1}H NMR** (300 MHz, DMSO-d_6) δ 12.92 (s, 1H), 10.80 (brs, 1H), 7.65 (dd, *J* = 8.6, 2.2 Hz, 1H), 7.53 (d, *J* = 2.2 Hz, 1H), 7.11 (d, *J* = 8.6 Hz, 1H), 6.91 (s, 1H), 6.51 (d, *J* = 2.2 Hz, 1H), 6.20 (d, *J* = 2.2 Hz, 1H), 4.98 (tt, *J* = 5.8, 2.6 Hz, 1H), 3.84 (s, 3H), 2.03 – 1.84 (m, 2H), 1.81 – 1.65 (m, 4H), 1.65 – 1.49 (m, 2H).**^{13}C NMR** (75 MHz, DMSO) δ 181.7, 164.2, 163.3, 161.4, 157.3, 153.0, 147.2, 122.9, 119.9, 112.2, 112.1, 103.7, 98.8, 94.0, 79.8, 55.7, 32.1, 23.6. **IR** (cm^{-1}) : 2932, 1651, 1557, 1495, 1435, 1367, 1331, 1250, 1169, 1036, 835, 725, 640. **HRMS-ESI** (*m /z*) : calcd for $[M+H]^+$ $C_{21}H_{21}O_6$, 369.1338 ; Found : 369.1334.

2-iodo-1-(2-methoxybenzo[d][1,3]dioxol-5-yl)ethan-1-one **4**

To a solution of chloromethyl-3,4-dihydroxyphenyl) ketone (10.0 g, 53.8 mmol) in trimethyl orthoformate (100 mL) was added PTSA monohydrate (500 mg) and the mixture was refluxed 2 days. The reaction mixture was the cooled down to rt, diluted with Et_2O (200 mL) washed with K_2CO_3 10% solution (3x100 mL), dried over $MgSO_4$, filtered, and concentrated to afford pure orthoester-protected catechol (16.8 g, 100%).

Mp : 140-142 °C. **^{1}H NMR** (300 MHz, Chloroform-*d*) *δ* 7.59 (dd, *J* = 8.2, 1.8 Hz, 1H), 7.49 (d, *J* = 1.8 Hz, 1H), 6.93 (d, *J* = 8.1 Hz, 1H), 6.92 (s, 1H), 4.61 (s, 2H), 3.41 (s, 3H). **^{13}C NMR** (75 MHz, Chloroform-*d*) *δ* 189.5, 150.9, 147.0, 129.1, 124.9, 120.2, 108.2, 108.0, 50.4, 45.7. **HRMS-ESI** (*m /z*) : calcd for $[M+H]^+$ $C_{10}H_{10}ClO_4$, 229.0268 ; Found : 229.0258. **IR** (cm^{-1}) : 3313, 1687, 1600, 1497, 1445, 1337, 1250, 1198, 1107, 989, 928, 806, 746.

To a solution of previous protected catechol (12.8 g, 53.8 mmol) in acetone (100 mL), was added NaI (12.1 g, 80.7 mmol, 1.5 equiv) and the reaction mixture was stirred at rt for 3 h. The reaction mixture was quenched with water and diluted with ether. The aqueous layer was extracted twice with Et_2O, and the combined organic layers were washed twice with 10% $NaHSO_3$ solution, dried over $MgSO_4$, filtered, concentrated, and the residue was obtained as a light brown oil, which crystallizes slowly on standing (16.87 g, 98%).

Mp : 73-75 °C. **^{1}H NMR** (300 MHz, Chloroform-*d*) δ 7.63 (dd, *J* = 8.2, 1.8 Hz, 1H), 7.51 (d, *J* = 1.8 Hz, 1H), 6.93 (s, 1H), 6.92 (d, *J* = 8.1 Hz, 1H), 4.29 (s, 2H), 3.42 (s, 3H). **^{13}C NMR** (75 MHz, Chloroform-*d*) *δ* 191.3, 150.8, 146.9, 128.3, 125.5, 120.2, 108.6, 108.0, 50.4, 1.5. **IR** (cm^{-1}) : 1660,

1599, 1491, 1435, 1392, 1263, 1196, 1144, 1022, 910, 879, 837, 769. **HRMS-ESI** (*m /z*) : calcd for $[M+H]^+$ $C_{10}H_{10}IO_4$, 320.9624 ; Found : 320.9614.

M30-E05

To a solution of 3'-O-cyclopentyl-diosmetin (**2**) (7.0 g, 19.0 mmol) in DMF (90 mL) was added K_2CO_3 (2.6 g, 19.0 mmol, 1 equiv). The reaction mixture was heated at 100 °C, iodide **4** (6.1 g, 19.0 mmol) in DMF (10 mL) was then added. The reaction was stirred 2 h at 100 °C, TLC monitoring showing the consumption of both starting materials. The reaction mixture was thus cooled down to rt, quenched with water (200 mL), extracted with DCM (3x 150 mL), and the combined organic layers were washed with brine (3x150 mL). The organic phase was dried over $MgSO_4$, filtered, concentrated and the residue was purified grossly on a silica gel chromatography (DCM: AcOEt, 90:10). The orange residue was then triturated with boiling MeOH (100 mL), and the mixture was cooled down to rt. The beige solid was filtered on a sintered glass, washed with a small amount of cold MeOH, and dried under vacuum to afford 7-O-(2-(2-methoxybenzo[d][1,3]dioxol-5-yl)-3'-O-cyclopentyl-diosmetin as a yellowish powder (6.72 g, 63%).

Mp : 161-163 °C. **^{1}H NMR** (300 MHz, Chloroform-*d*) δ 12.83 (s, 1H), 7.65 (dd, J = 8.2, 1.7 Hz, 1H), 7.54 (d, J = 1.7 Hz,1H), 7.47 (dd, J = 8.5, 2.2 Hz, 1H), 7.31 (d, J = 2.2 Hz, 1H), 6.98 (d, J = 8.1 Hz, 1H), 6.95 (s, 1H), 6.94 (d, J = 8.5 Hz, 1H), 6.55 (s, 1H), 6.53 (d, J = 2.3 Hz, 1H), 6.34 (d, J = 2.2 Hz, 1H), 5.30 (s, 2H), 4.85 (tt, J = 6.2, 2.9 Hz, 2H), 3.91 (s, 3H), 3.43 (s, 3H), 2.06 – 1.77 (m, 6H), 1.72 – 1.57 (m, 2H). **^{13}C NMR** (75 MHz, Chloroform-*d*) δ 191.4, 182.5, 164.48, 163.7, 162.4, 157.7, 153.5, 151.1, 148.0, 147.1, 129.0, 124.5, 123.6, 120.2, 120.1, 112.4, 111.7, 108.2, 107.9, 106.2, 104.7, 98.4, 93.8, 80.9, 70.6, 56.2, 50.4, 32.9, 24.2. **IR** (cm^{-1}) : 2955, 1693, 1654, 1597, 1497, 1437, 1354, 1253, 1177, 1093, 904, 768. **HRMS-ESI** (*m /z*) : calcd for $[M+H]^+$ $C_{31}H_{29}O_{10}$, 561.1761 ; Found : 561.1747.

To a suspension of 7-O-(2-(2-methoxybenzo[d][1,3]dioxol-5-yl)-3'-O-cyclopentyl-diosmetin (3.0 g, 5.35 mmol, 1 equiv) in AcOEt:THF:MeOH mixture (30:40:30 mL) in a Parr reactor was added $Pd(OH)_2$ 20% on carbon (500 mg) and HCl 35% (100 µL) was added. The whole mixture was stirred (1200 rpm) at rt under H_2 atmosphere (10 bars) for 15 h. The mixture was then filtered on Celite, and concentrated on a rotavapor. The residue was triturated in with hot MeOH (20 mL), cooled down and Et_2O was added (20 mL), continuing the trituration at rt. The beige powder was then isolated by filtration to afford M30-E05 (1.32 g) and the filtrate was concentrated and purified grossly on a silica gel chromatography (DCM:Acetone, 9:1). The purified product was triturated again in a 1:1 MeOH:Et_2O mixture to give additional M30-E05 (350 mg). The combined fraction gives M30-E05 as a light beige powder (1.67 g, 62%).

Mp : 205-207 °C. **^{1}H NMR** (300 MHz, DMSO-*d*$_6$) δ 12.92 (s, 1H), 8.72(s, 1H), 8.67 (s, 1H), 7.67 (dd, J = 8.7, 1.2 Hz, 1H), 7.54 (d, J = 1.2 Hz, 1H), 7.11 (d, J = 8.7 Hz, 1H), 6.96 (s, 1H), 6.78 (d, J = 1.7 Hz, 1H), 6.78 (d, J = 1.2 Hz, 1H), 6.67 (d, J = 8.0 Hz, 1H), 6.56 (dd, J = 8.0, 1.2 Hz, 1H), 6.34 (d, J = 1.7 Hz, 1H), 5.00 (tt, J = 6.2, 1.7 Hz, 1H), 4.23 (t, J = 6.9 Hz, 2H), 3.84 (s, 3H), 2.88 (t, J = 6.9 Hz, 2H), 2.04 – 1.87 (m, 2H), 1.86 – 1.66 (m, 4H), 1.66 – 1.49 (m, 2H).**^{13}C NMR** (75 MHz, DMSO-*d*$_6$) δ 182.0, 164.4, 163.6, 161.2, 157.3, 153.1, 147.2, 145.1, 143.8, 128.5, 122.7, 120.1, 119.6, 116.4, 115.5, 112.1, 112.0, 104.7, 103.9, 98.3, 93.2, 79.8, 69.5, 67.0, 55.8, 32.2, 23.7. **IR** (cm^{-1}) : 3402, 3161, 2953, 1654, 1600, 1499, 1438, 1251, 1165, 1024, 849, 808, 765. **HRMS-ESI** (*m /z*) : calcd for $[M+H]^+$ $C_{29}H_{29}O_8$, 505.1862 ; Found : 505.1849.

Copies of ^{1}H and ^{13}C NMRs of M30-E05 and Chemical Intermediates

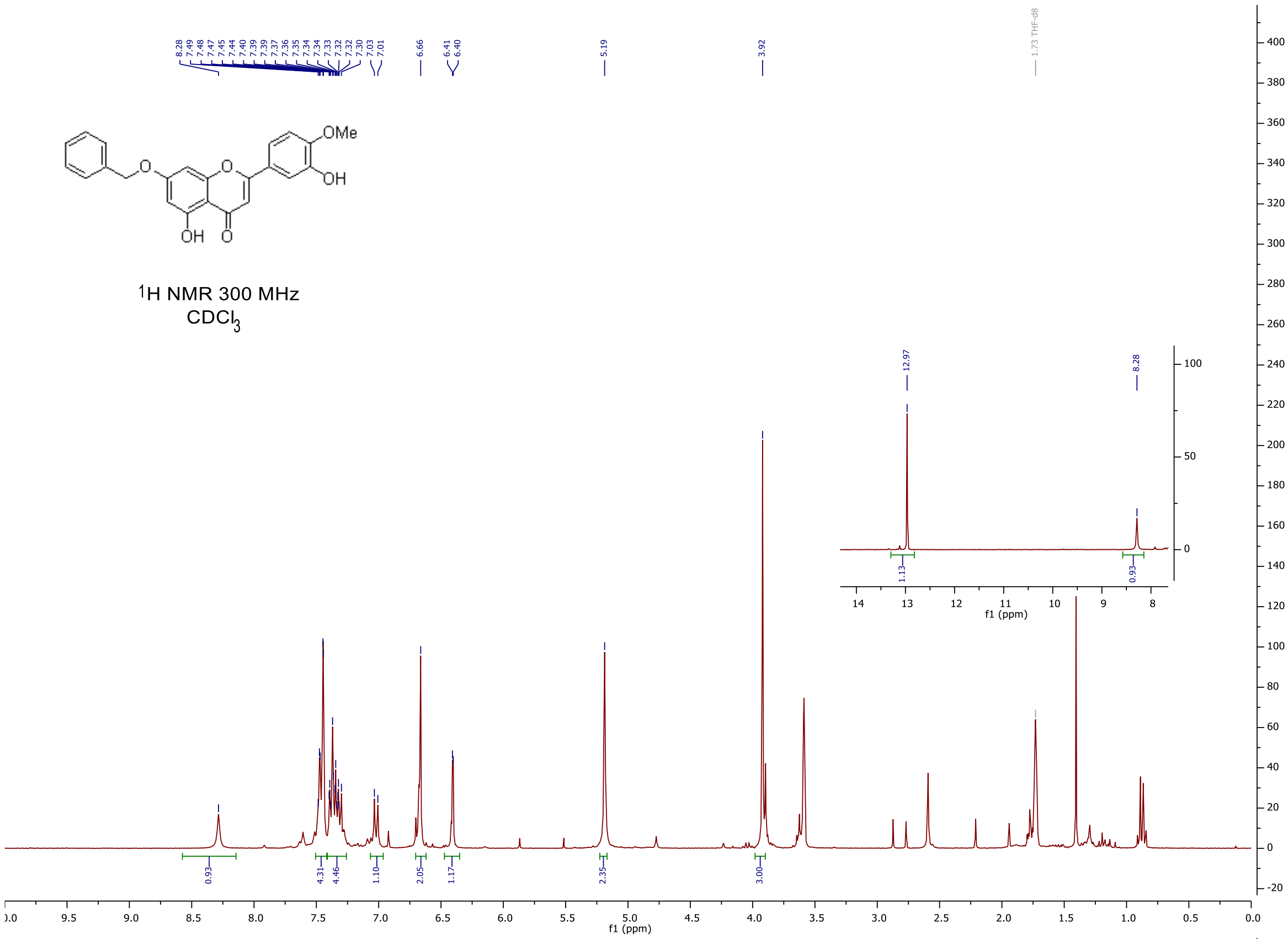

OMe
O
O
OH
OH
O
1H NMR 300 MHz
CDCl3
8.28
7.49
7.48
7.47
7.45
7.44
7.40
7.39
7.39
7.37
7.36
7.35
7.34
7.34
7.33
7.32
7.32
7.30
7.03
7.01
6.66
6.41
6.40
5.19
3.92
1.73 THF-d8
12.97
8.28
f1 (ppm)

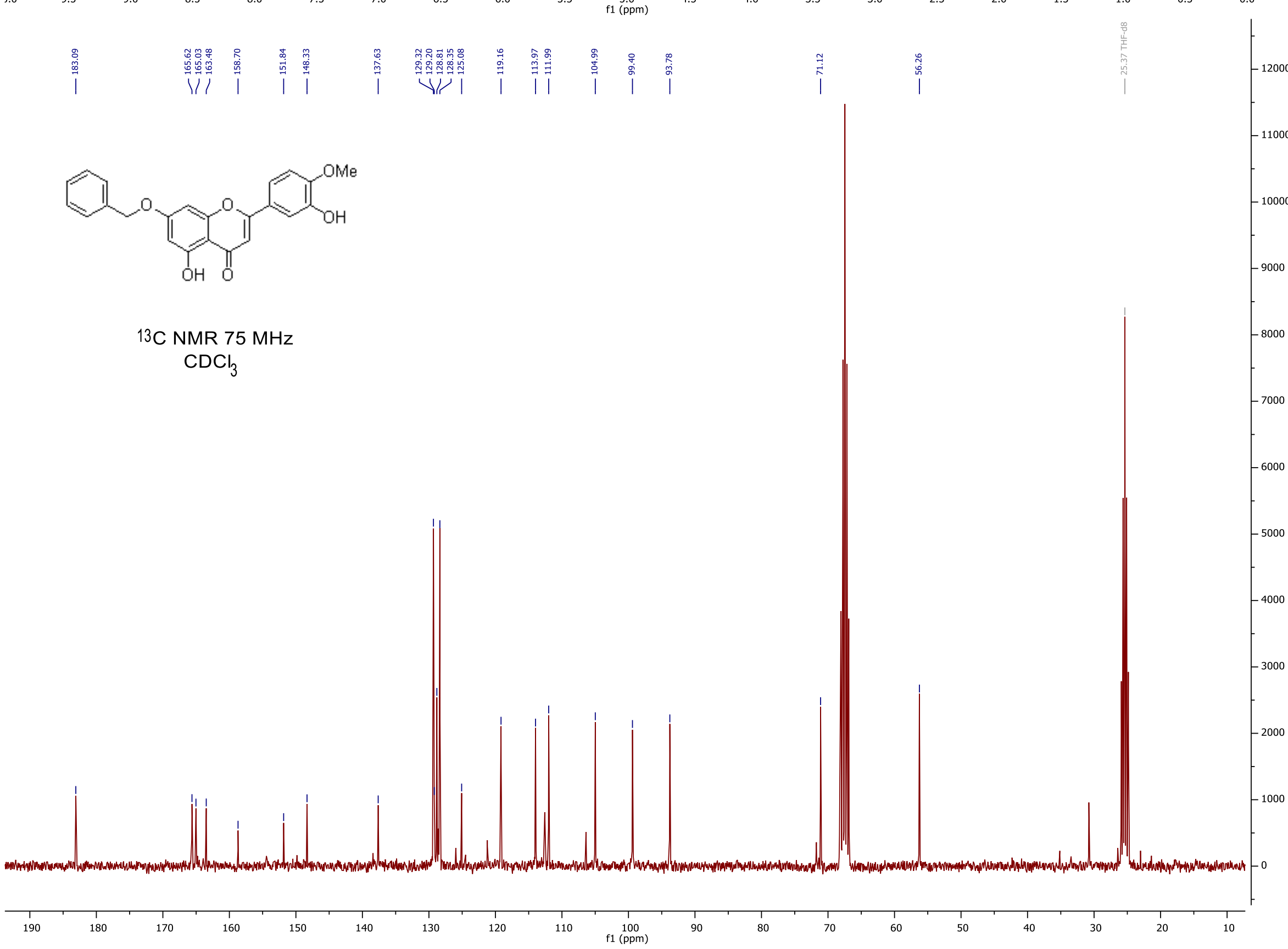

OMe
O
O
OH
OH
O
13C NMR 75 MHz
CDCl3
183.09
165.62
165.03
163.48
158.70
151.84
148.33
137.63
129.32
129.20
128.81
128.35
125.08
119.16
113.97
111.99
104.99
99.40
93.78
71.12
56.26
25.37 THF-d8
f1 (ppm)

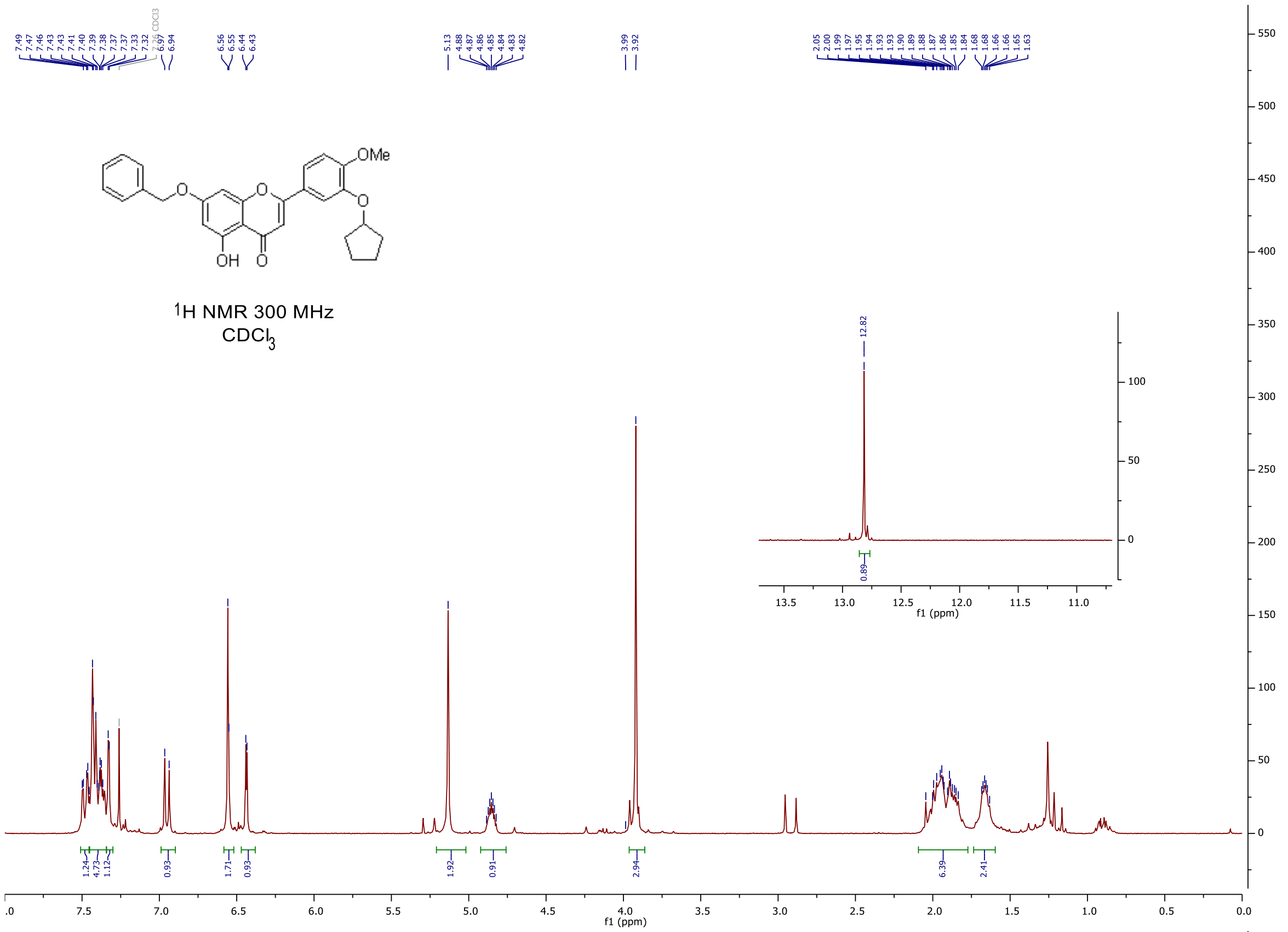
OMe
O
OH
1H NMR 300 MHz
CDCl3
7.49 7.47 7.46 7.43 7.43 7.41 7.40 7.39 7.38 7.37 7.37 7.33 7.32 6.97 6.94
6.56 6.55 6.44 6.43
5.13 4.88 4.87 4.86 4.85 4.84 4.83 4.82
3.99 3.92
2.05 2.00 1.99 1.97 1.95 1.94 1.93 1.93 1.90 1.89 1.88 1.87 1.86 1.85 1.84 1.68 1.68 1.66 1.66 1.65 1.63
12.82
f1 (ppm)
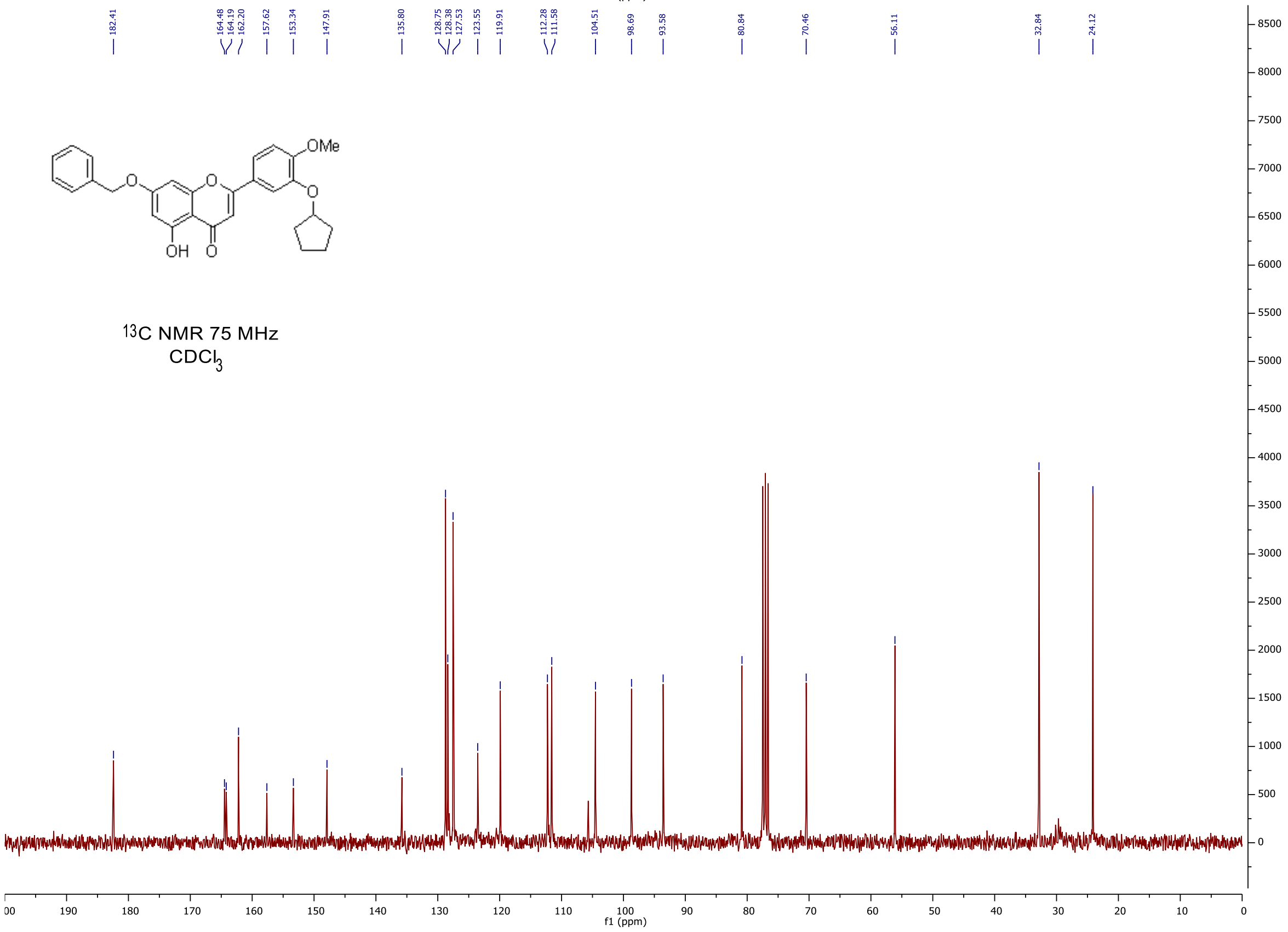
OMe
O
OH
13C NMR 75 MHz
CDCl3
182.41
164.48 164.19 162.20 157.62 153.34 147.91
135.80 128.75 128.38 127.53 123.55 119.91
112.28 111.58 104.51 98.69 93.58
80.84 70.46 56.11 32.84 24.12
f1 (ppm)

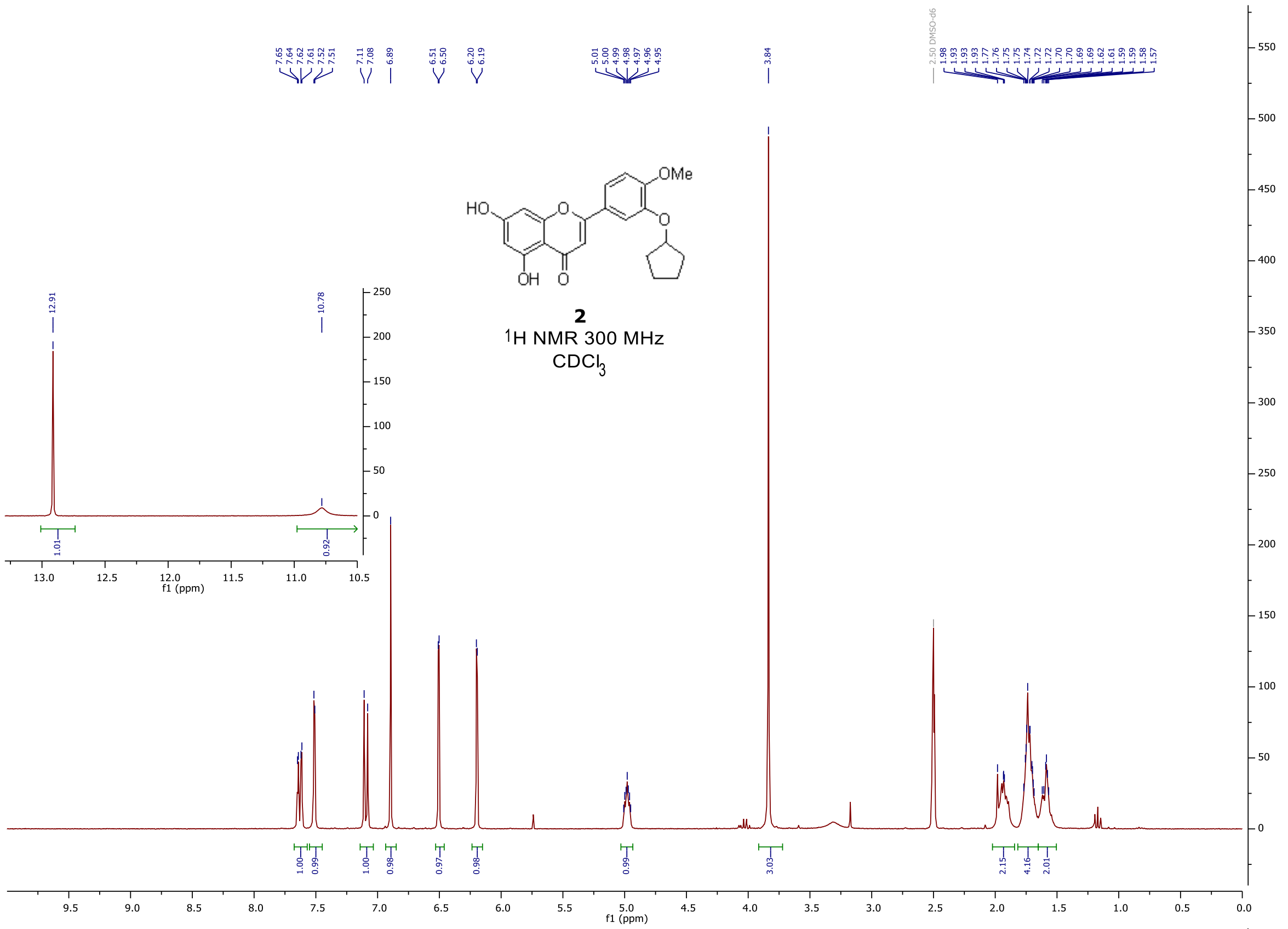
OMe
HO
OH
O
2
1H NMR 300 MHz
CDCl3
f1 (ppm)

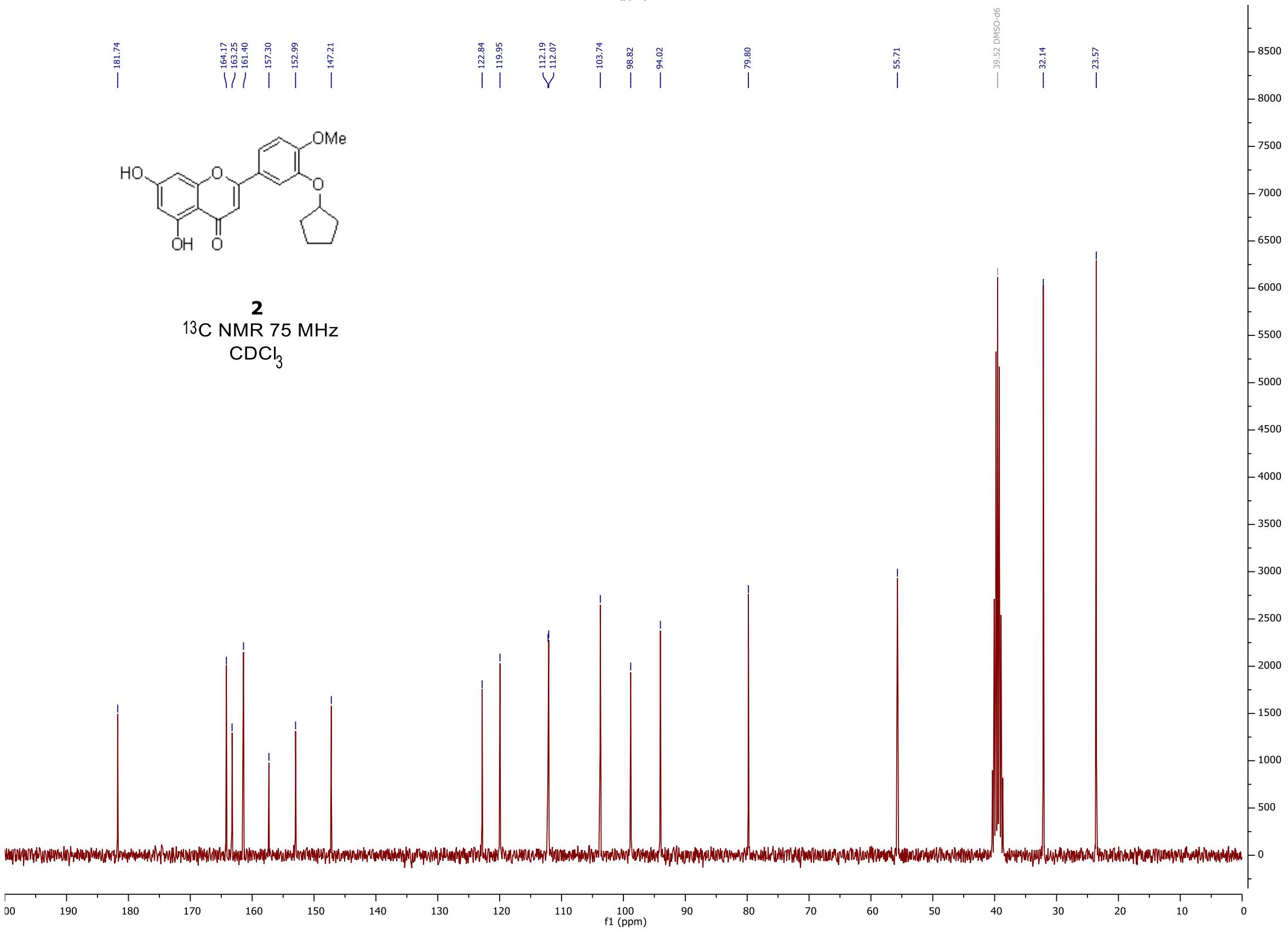
OMe
HO
OH
O
2
13C NMR 75 MHz
CDCl3
f1 (ppm)

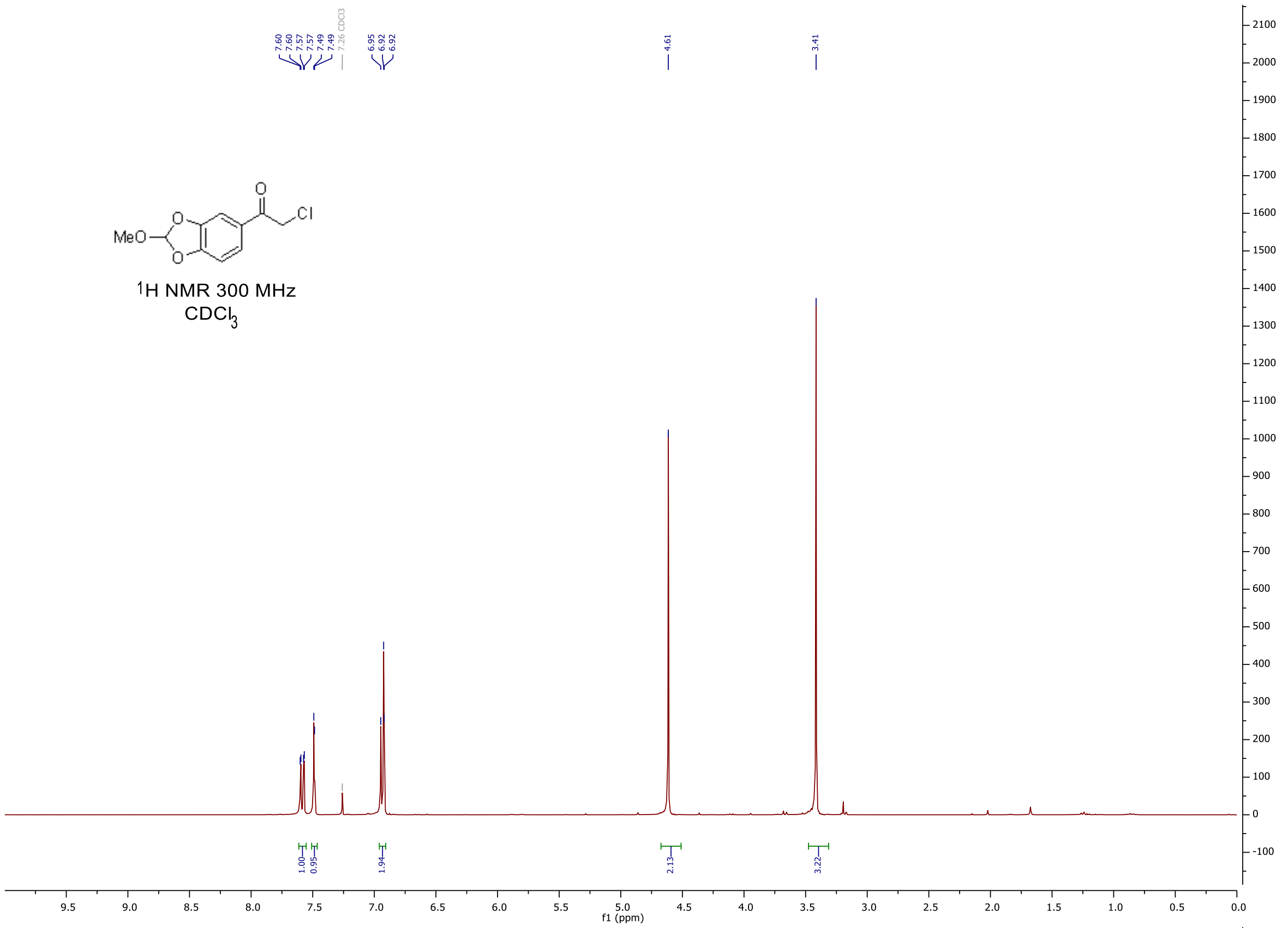
MeO
O
O
O
Cl
1H NMR 300 MHz
CDCl3
7.60
7.60
7.57
7.57
7.49
7.49
7.26 CDCl3
6.95
6.92
6.92
4.61
3.41
1.00
0.95
1.94
2.13
3.22
f1 (ppm)

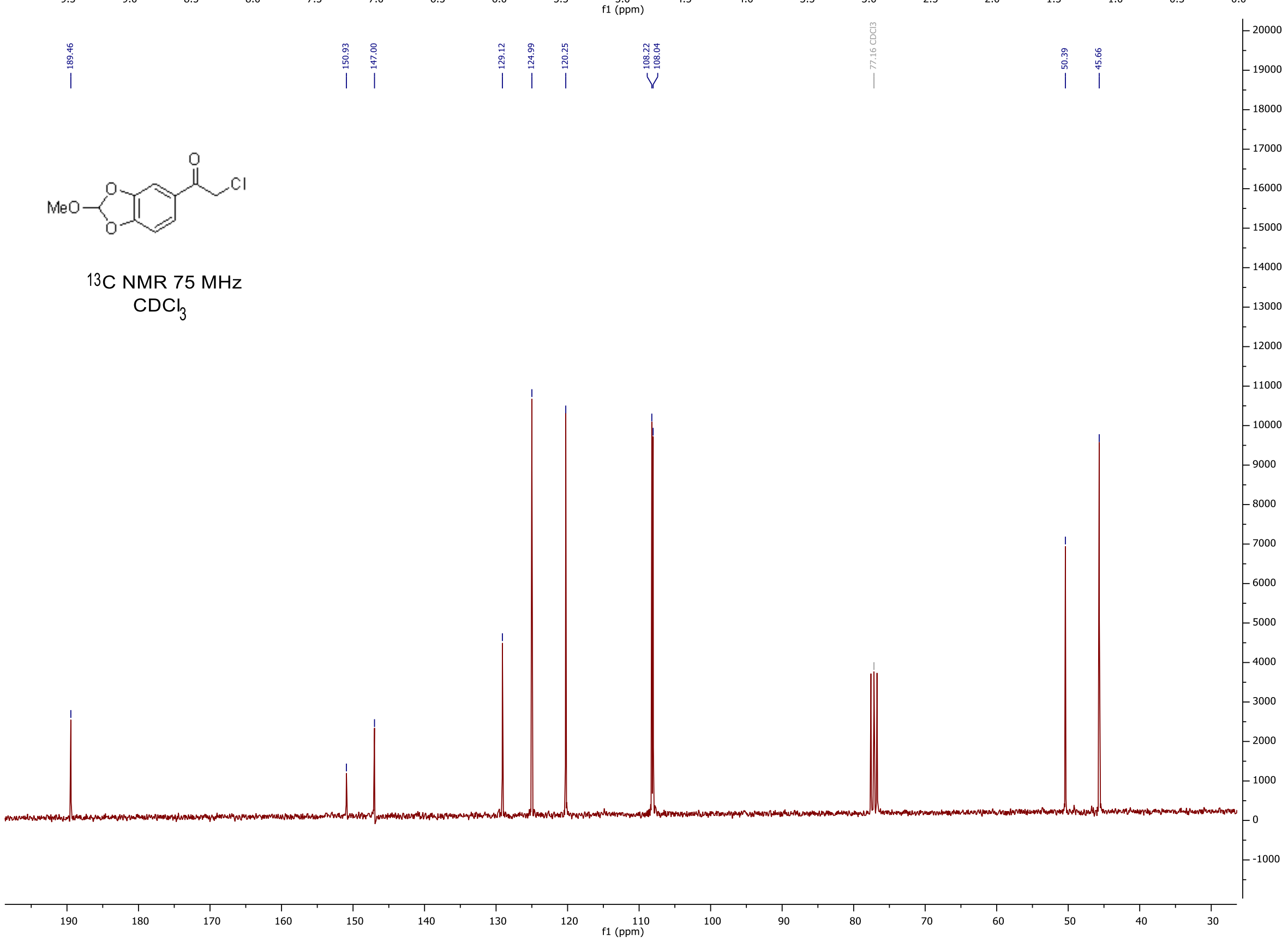
MeO
O
O
O
Cl
13C NMR 75 MHz
CDCl3
189.46
150.93
147.00
129.12
124.99
120.25
108.22
108.04
77.16 CDCl3
50.39
45.66
f1 (ppm)

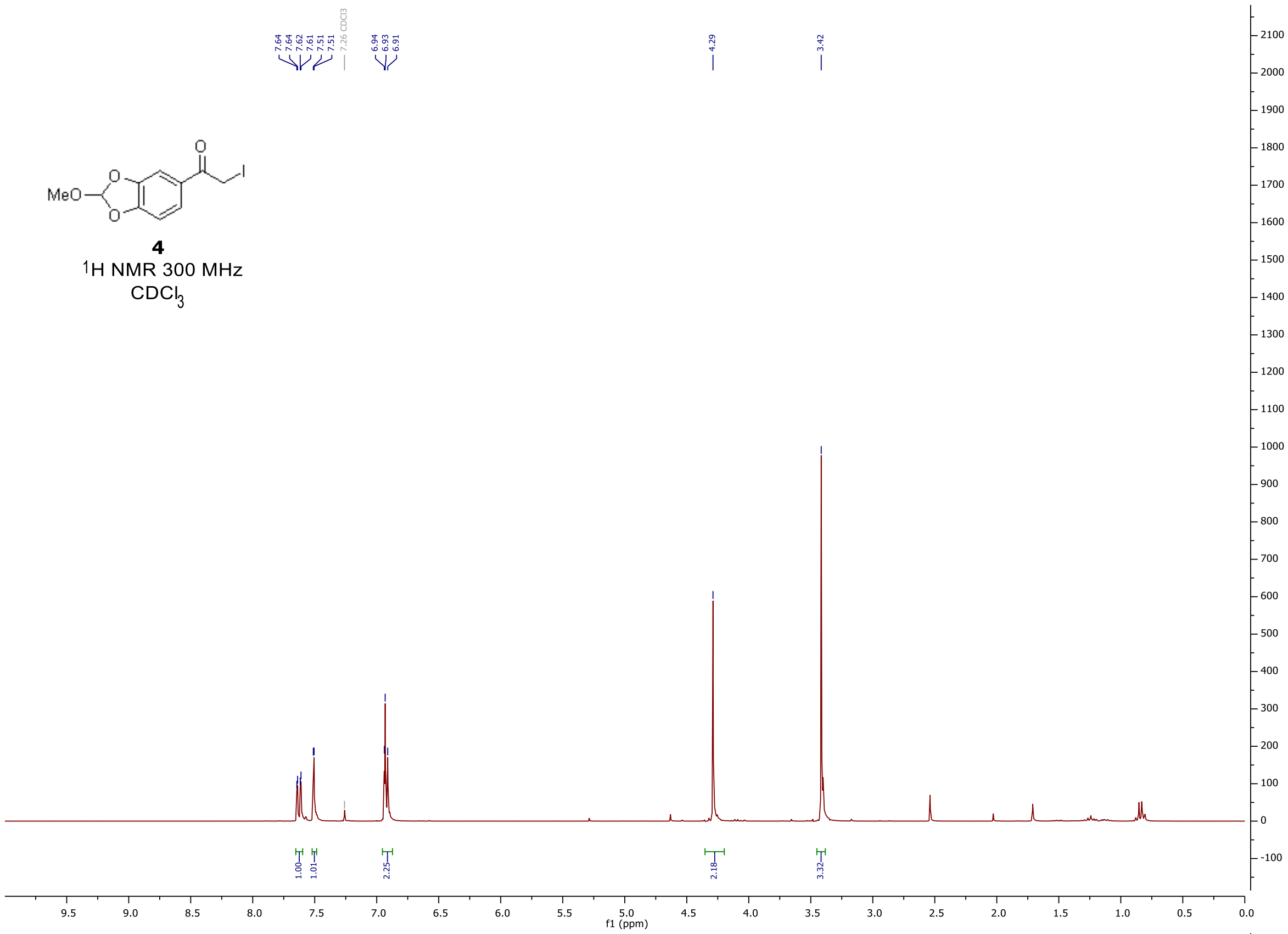
MeO
O
O
O
I
4
1H NMR 300 MHz
CDCl3
7.64
7.64
7.62
7.61
7.51
7.51
7.26 CDCl3
6.94
6.93
6.91
4.29
3.42
1.00
1.01
2.25
2.18
3.32
f1 (ppm)

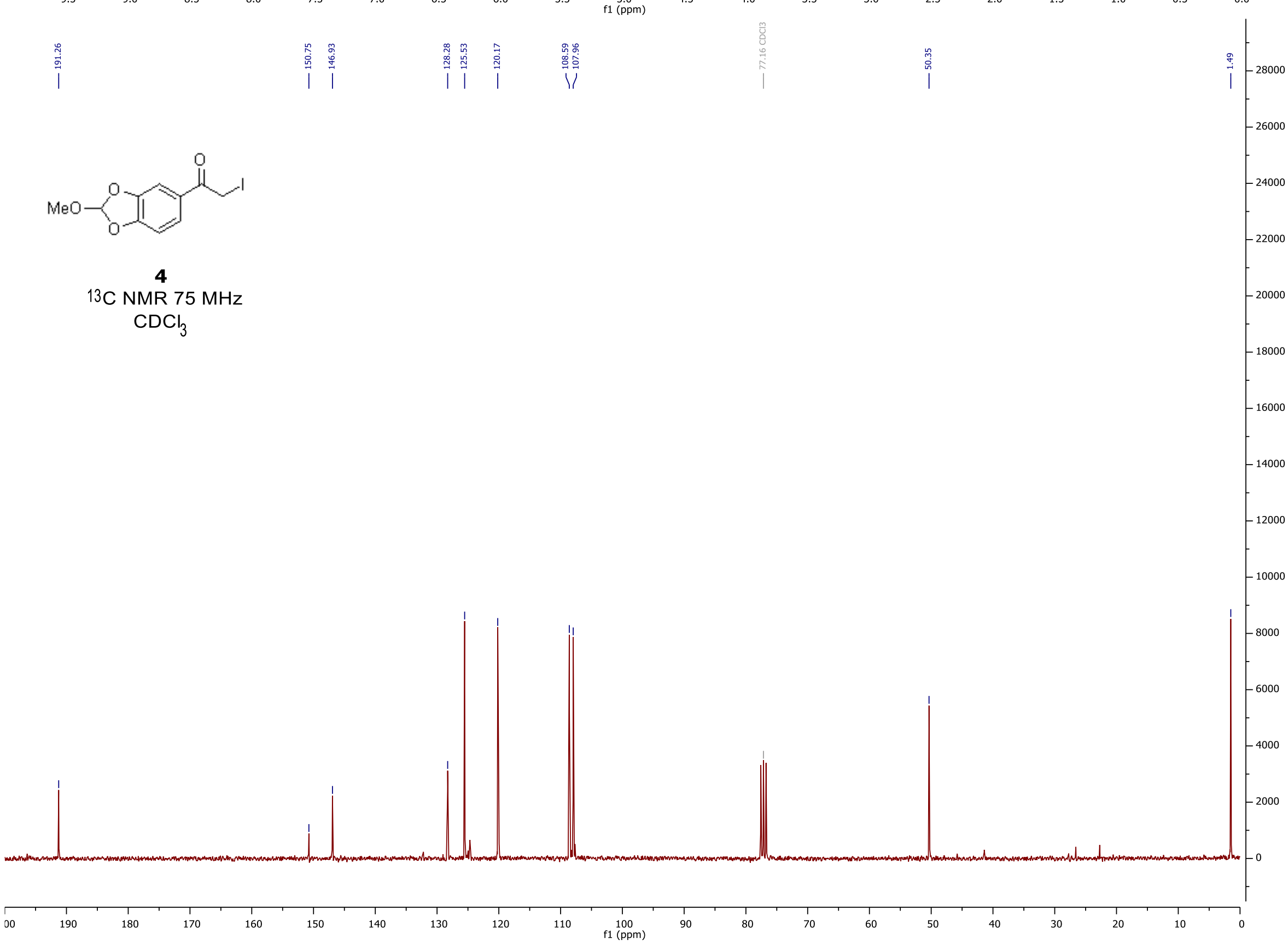
MeO
O
O
O
I
4
13C NMR 75 MHz
CDCl3
191.26
150.75
146.93
128.28
125.53
120.17
108.59
107.96
77.16 CDCl3
50.35
1.49
f1 (ppm)

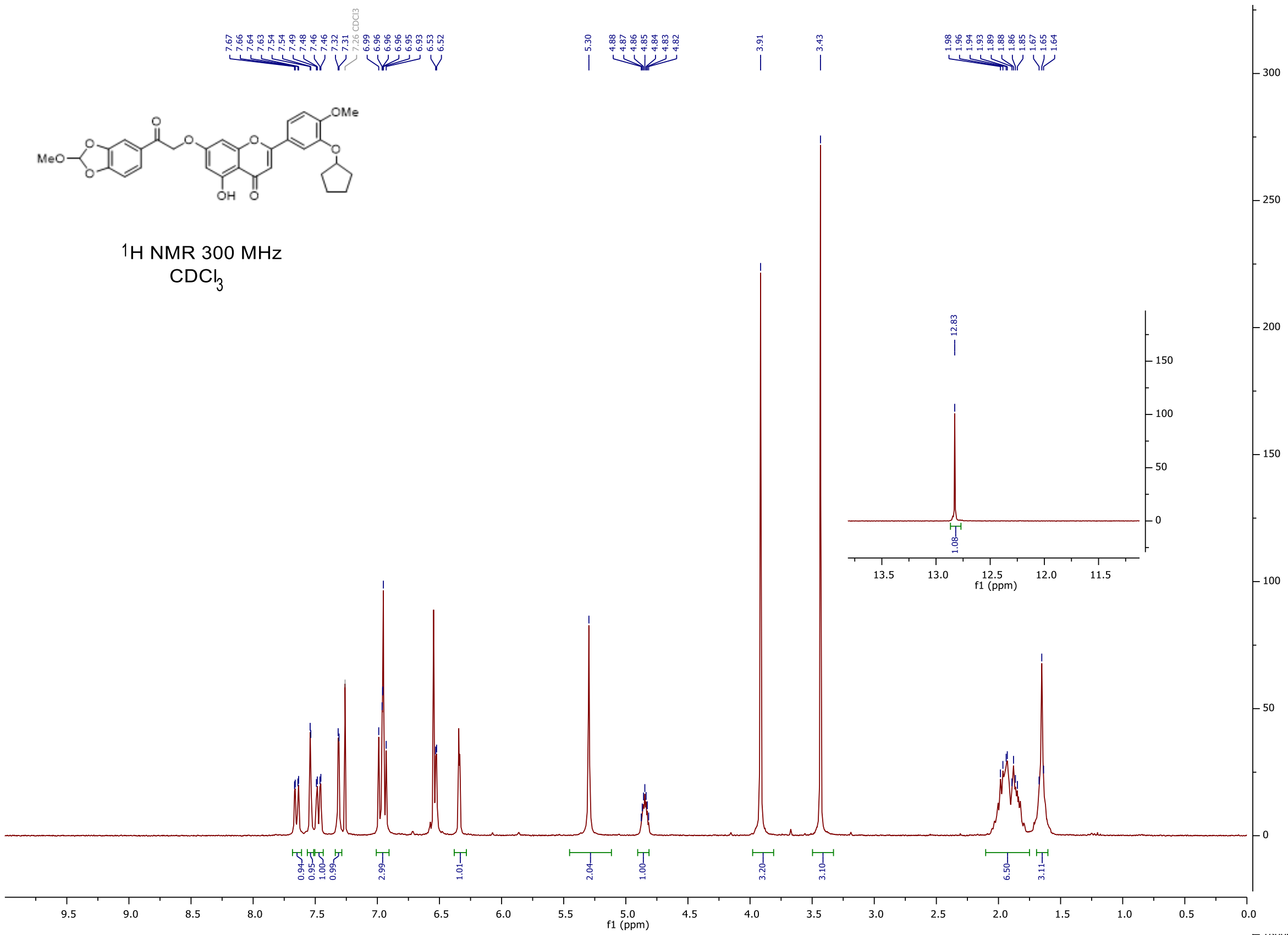
MeO
OMe
OH
^{1}H NMR 300 MHz
$CDCl_3$
f1 (ppm)

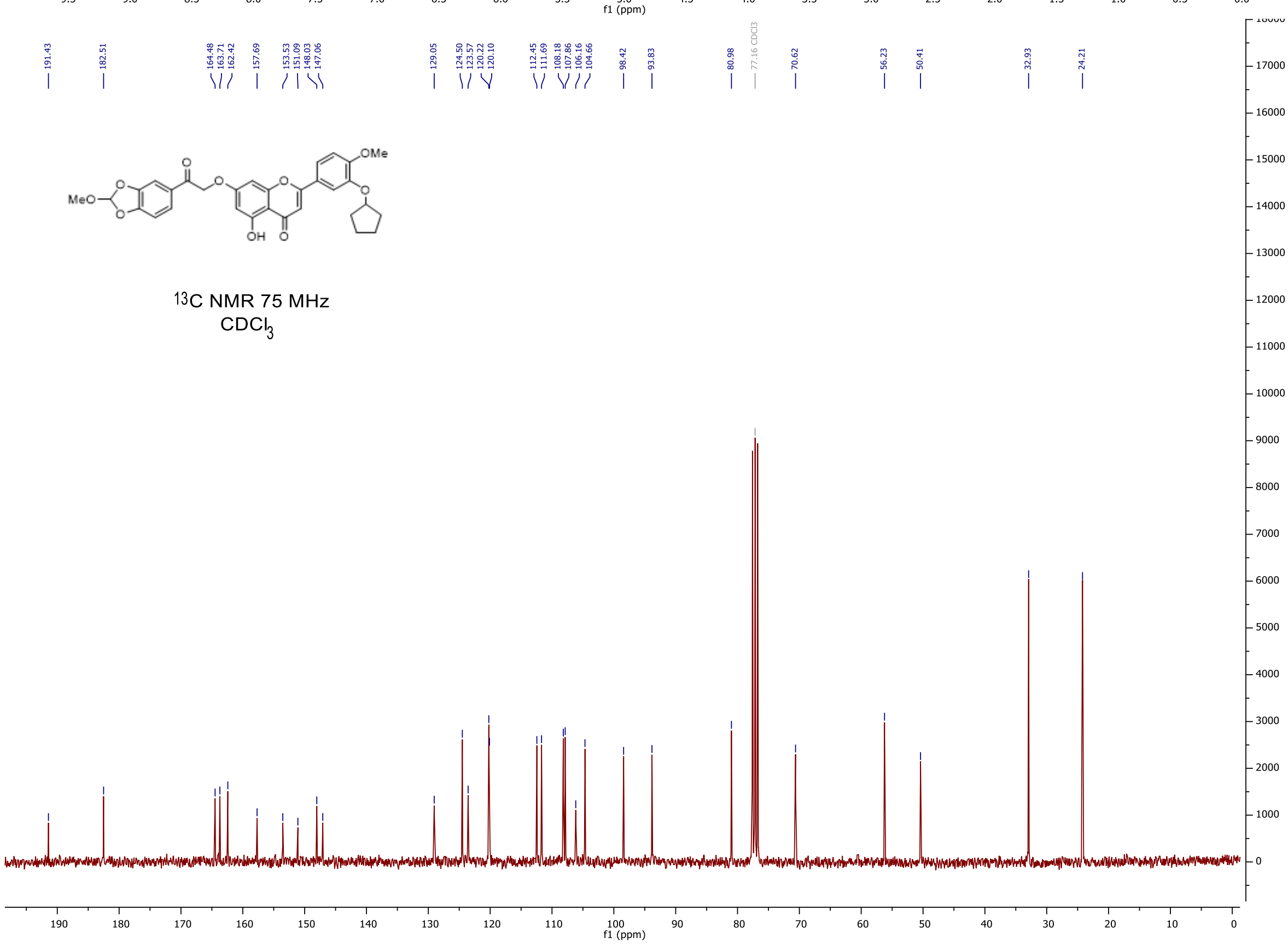
MeO
OMe
OH
^{13}C NMR 75 MHz
$CDCl_3$
f1 (ppm)

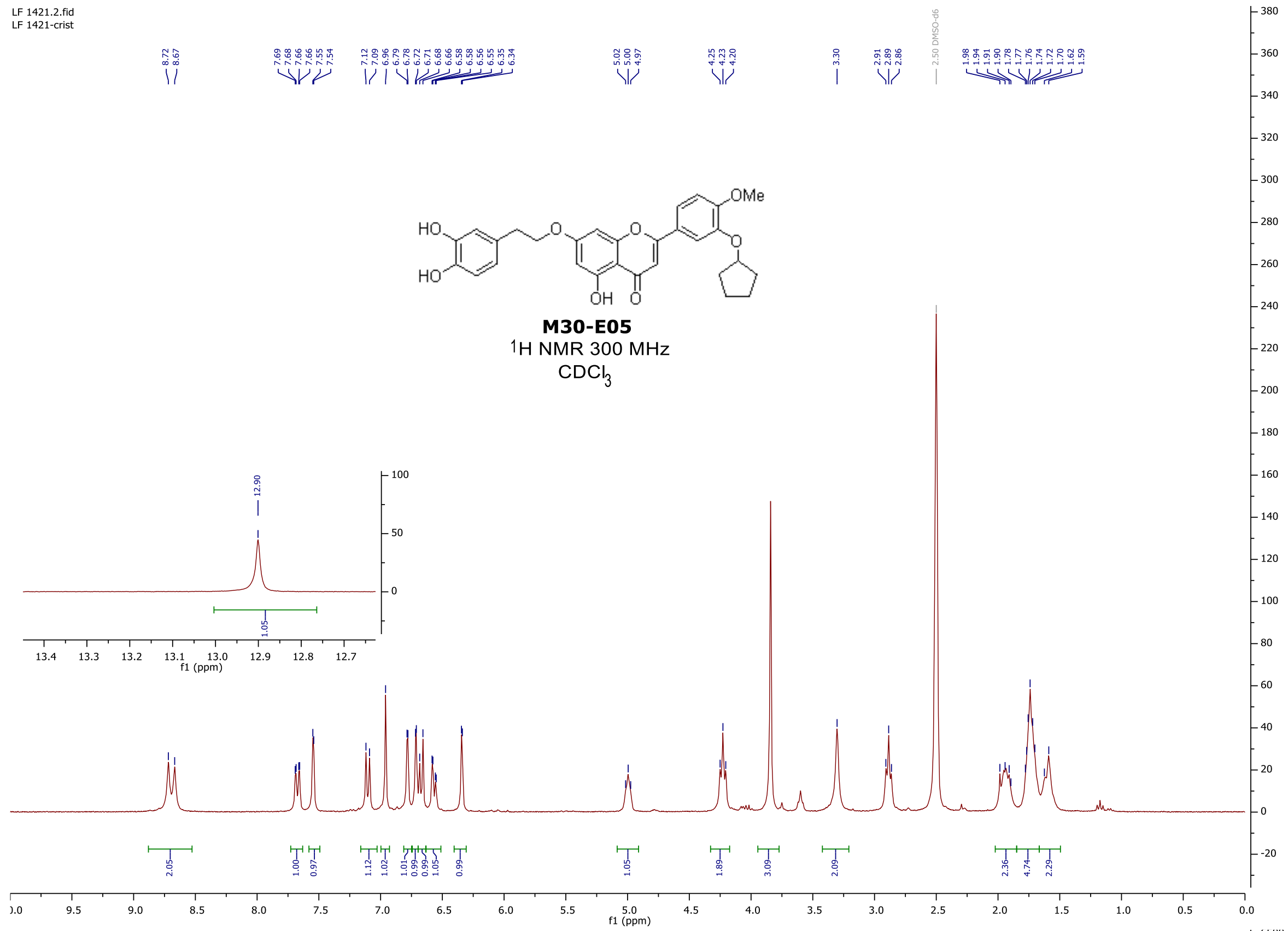
LF 1421.2.fid
LF 1421-crist
M30-E05
1H NMR 300 MHz
CDCl3
f1 (ppm)

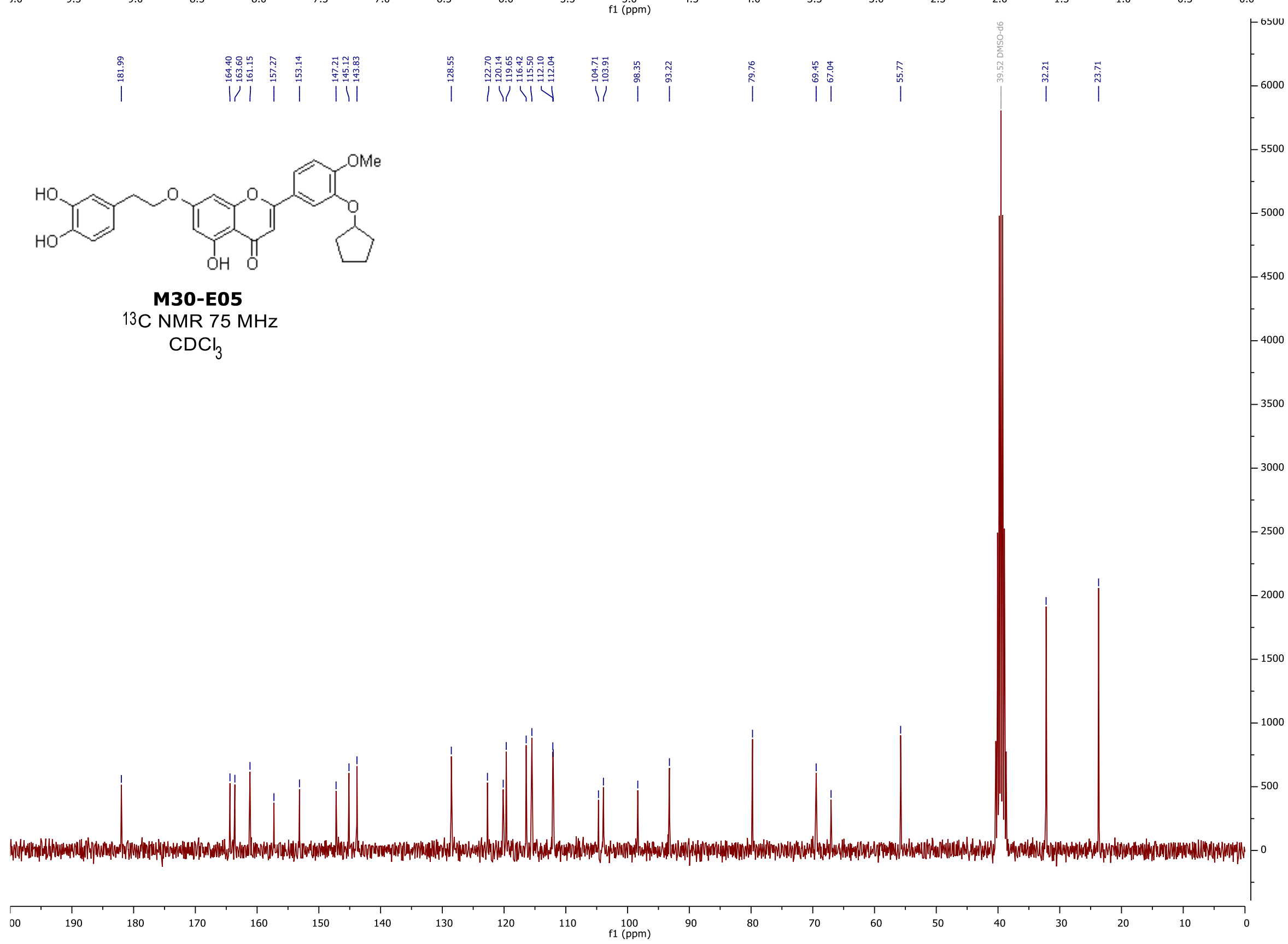
M30-E05
13C NMR 75 MHz
CDCl3
f1 (ppm)
